# Uncovering protein–protein interactions through a team-based undergraduate biochemistry course

**DOI:** 10.1371/journal.pbio.2003145

**Published:** 2017-11-01

**Authors:** David L. Cookmeyer, Emily S. Winesett, Bashkim Kokona, Adam R. Huff, Sabina Aliev, Noah B. Bloch, Joshua A. Bulos, Irene L. Evans, Christian R. Fagre, Kerilyn N. Godbe, Maryna Khromava, Daniel M. Konstantinovsky, Alexander E. Lafrance, Alexandra J. Lamacki, Robert C. Parry, Jeanne M. Quinn, Alana M. Thurston, Kathleen J. S. Tsai, Aurelio Mollo, Max J. Cryle, Robert Fairman, Louise K. Charkoudian

**Affiliations:** 1 Department of Chemistry, Haverford College, Haverford, Pennsylvania, United States of America; 2 Department of Biology, Haverford College, Haverford, Pennsylvania, United States of America; 3 The Monash Biomedical Discovery Institute, EMBL Australia, Monash University, Clayton, Victoria, Australia; 4 The Department of Biochemistry and Molecular Biology and ARC Centre of Excellence in Advanced Molecular Imaging, Monash University, Clayton, Victoria, Australia

## Abstract

How can we provide fertile ground for students to simultaneously explore a breadth of foundational knowledge, develop cross-disciplinary problem-solving skills, gain resiliency, and learn to work as a member of a team? One way is to integrate original research in the context of an undergraduate biochemistry course. In this Community Page, we discuss the development and execution of an interdisciplinary and cross-departmental undergraduate biochemistry laboratory course. We present a template for how a similar course can be replicated at other institutions and provide pedagogical and research results from a sample module in which we challenged our students to study the binding interface between 2 important biosynthetic proteins. Finally, we address the community and invite others to join us in making a larger impact on undergraduate education and the field of biochemistry by coordinating efforts to integrate research and teaching across campuses.

## Introduction

A call to action by the American Association of the Advancement of Science (AAAS) and the National Science Foundation (NSF) lists unifying teaching and research as the top challenge for faculty teaching biology [1]. According to an NSF-funded think tank, there is a particular need for the development of course-based undergraduate research experiences (CUREs) in protein biochemistry [2]. By integrating higher-level goals and philosophies of research, the teaching laboratory is transformed into fertile ground for students to develop cross-disciplinary technical skills, confront unexpected findings, hone troubleshooting skills, discuss ethics of research, and work as a member of a team. Close mentorship from instructors enables each student to work in their zone of proximal discomfort, thereby maximizing learning outcomes in a manner often not possible in other classroom and training environments. The benefits from such experiences serve future scientists and nonscientists alike.

Herein we present a template workflow for course design and execution of an interdisciplinary and cross-departmental undergraduate biochemical laboratory course focused on protein chemistry. Since this course is necessarily rooted in original research, we concomitantly share biochemical results that students uncovered and place these findings in the context of the field. Some of our findings from this laboratory have already been published in the primary research literature [3], and we chose to share other significant findings here to illustrate the dual pedagogical and research outcomes to a wider audience. In sharing our story, we hope to contribute to the growing body of CUREs described in the literature by providing a novel example focused on protein chemistry and emphasizing the benefits of additional learning goals prioritizing student growth in networking, teamwork, and communication skills.

## Many hands make light work

Solving the big problems of our age requires the ability to think critically and creatively, communicate clearly, and span the skill sets and scientific language differences of various disciplines. Therefore, our “Biochemistry Superlab” course is designed to integrate these objectives with field-specific goals (Box 1). This course builds on a history of research-based courses at Haverford College and at other institutions [4,5] and stems from the identification of an original research problem amenable to the “many hands make light work” approach. While students carry out independent experiments in pairs, we work as a unified team in pursuit of answers to specific hypotheses tied to a central question.

**Box 1. Defining the course to be a win, win, win for students, faculty, and the field. (^∧^ represents student-specific goals; ^#^ represents faculty-specific goals)**

**Define pedagogical goals of the course.^#^**

- Gain proficiency in relevant computer-based tools (ex: PyMOL).^∧^
- Develop competency in making materials (ex: plasmids, proteins, organic molecules).^∧^
- Conduct assays and analyze results.^∧^
- Learn to use state-of-the-art equipment to obtain data.^∧^
- Locate, read, and understand primary journal articles and scientific reviews.^∧^
- Navigate the scientific literature to inform practical laboratory techniques and contextualize research findings.^∧^
- Work safely and efficiently as a member of a research team.^∧^
- Understand ethical issues associated with research project.^∧^
- Troubleshoot unexpected experimental results.^∧^

**Create opportunities to communicate science.^#^**

- Communicate results and obstacles at weekly “group meetings.”^∧^
- Work as a team to define a “divide and conquer” approach to test group hypotheses.^∧,#^
- Create and maintain a class blog to celebrate the trials, tribulations, and discoveries of our research team.^∧,#^
- Write, review, and revise manuscripts and proposals.^∧,#^
- Summarize findings in oral presentation.^∧^
- Discuss ethical challenges relevant to the course theme.^∧,#^

**Build a network.^#^**

- Build a tiered mentoring system comprised of peers, teaching assistants, instructors, and collaborators.^#^
- Coteach the lab course with faculty from diverse backgrounds.^#^
- Invite guest lecturers from a range of backgrounds/institutions.^#^

**Contribute to the field.^∧,#^**

- Publish manuscripts with students earning co-authorship.^∧,#^
- Contribute to community resources.^∧,#^

**Example themes (key biochemistry concepts).**

- Probe a protein–protein or protein–substrate interaction using structural functional assays.
- Characterize secondary metabolites from a bacterial strain.
- Discover common cellular processes in developmental pathways and disease.

Our Biochemistry Superlab is offered annually to 16–20 students (typically biology and chemistry majors with a concentration in biochemistry) and is cotaught by faculty with research interests that span the chemistry–biology interface. While the course formally meets twice a week for a total of 2 1-hour discussion-style lectures and 2 3-hour laboratory sessions (S1 Manual), when appropriate, students take advantage of the flexibility afforded by 24-hour access to the laboratories to pursue their research goals. This scheduling flexibility is intended to maximize efficiency and not necessarily to add to the total number of hours spent in the lab per week. To ensure a safe and healthy working environment, students are given monitored electronic access to the laboratory and make use of a “buddy system” while using instruments deemed safe for use out of scheduled class time.

We scaffold the course with opportunities to build a broader scientific network, communicate science to experts and science “outsiders,” and expand the impact of our work beyond the tight-knit college environment (Box 1). About half the lecture slots are led by the faculty to provide students with the information necessary to execute and understand their experimental work (e.g., plasmid construction, protein expression/purification, biophysical characterization techniques), whereas the other half are dedicated to theme-based guest speakers, journal clubs, and “lab group meetings” that focus on troubleshooting and analyzing data as a team. Assessment and experimental vetting are also critical aspects of the course flow (S1 Assessment). The course design and execution is truly a team effort: In the case of this article, the 22 coauthors represent the students, faculty, staff, and collaborators who worked as a team to design, execute, analyze, and communicate the research project outlined in the case study below. We also worked as a team in writing this article and responding to reviewer comments.

## Case study: The sky’s the limit

**Fig 1:**
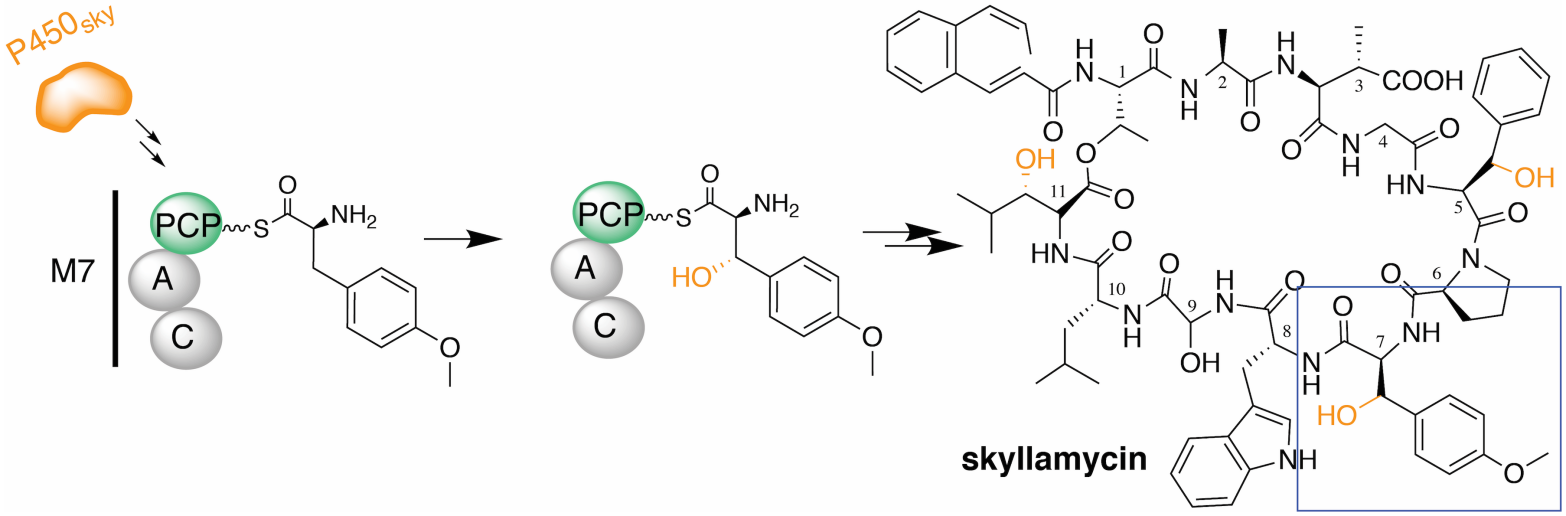
In a semester of Biochemistry Superlab, students investigated the protein–protein interactions involved in the β-hydroxylation of the natural product skyllamycin. The skyllamycin peptide is constructed by *Streptomyces* bacteria via a NRPS involving 11 biosynthetic modules (“M”), composed of catalytic domains such as the A, PCP, and C domains. The *in trans* cytochrome P450 (P450_sky_, orange) interacts with PCP-bound amino acids on modules 5, 7, and 11 to install β-hydroxyl groups (highlighted in orange on the structure of skyllamycin, right). As a class, we tackled the central question: What is the biochemical basis for the selectivity of the interaction of PCP from module 7 with P450_sky_ to install the hydroxyl group on the L-(OMe)-Tyr (incorporated at the boxed position of skyllamycin)? A, adenylation; C, condensation; NRPS, non-ribosomal peptide synthetase; PCP, peptidyl carrier protein.

Probing protein–protein interactions involved in antibiotic biosynthesis is an excellent course theme for teaching relevant interdisciplinary approaches and promoting discussions involving molecular interactions from a chemical and biological perspective. In a recent Biochemistry Superlab module, we started the semester by learning about the role of an *in trans* bacterial cytochrome P450 enzyme (P450_sky_) that catalyzes the β-hydroxylation of amino acid precursors during the biosynthesis of the natural product skyllamycin (Fig 1; S1 Manual). Previous studies suggest that the oxidation of L-amino acid substrates occurs while the amino acid is covalently bound to peptidyl carrier proteins (PCPs) [6,7] and that the protein interactions between PCPs and P450_sky_ determine whether or not oxidation occurs on any particular residue [3,6].

**Fig 2:**
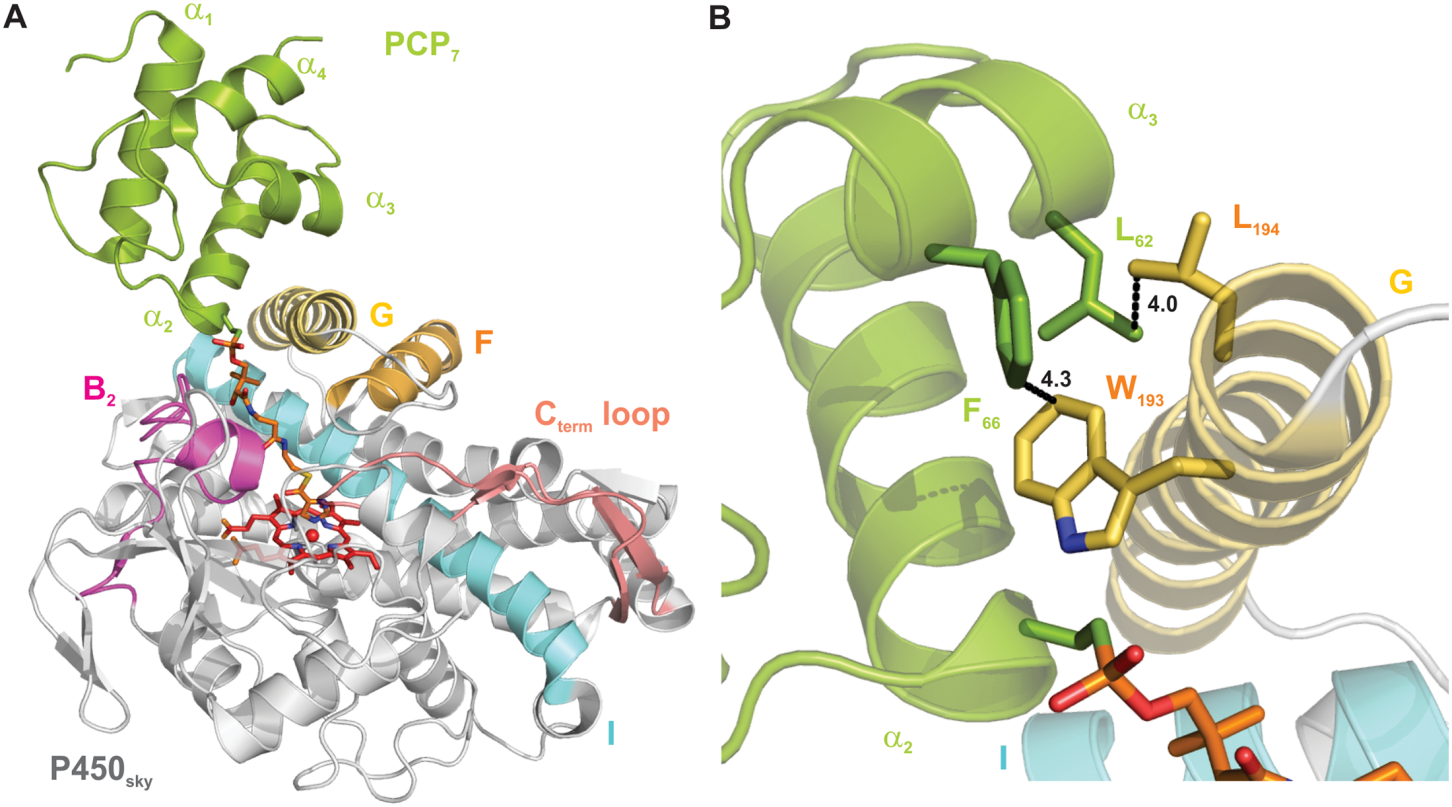
Structures of the skyllamycin NRPS PCP domain (PCP7_sky_, green) bound to a hydroxylating cytochrome P450 (P450_sky_, multicolored). Students visualized this structure in PyMOL (A) and evaluated the roles of 4 amino acid residues at the P450_sky_–PCP7_sky_ interface (B). See Box 2 for details. NRPS, non-ribosomal peptide synthetase; PCP, peptidyl carrier protein.

Students worked in pairs to study the published crystal structure of a stabilized P450_sky_–PCP7_sky_ complex (Fig 2A) in which the PCP from module 7 (“PCP7_sky_”) is tethered to P450_sky_ via a covalent inhibitor [6,7]. As a class, we developed a central question: “What biochemical interactions facilitate P450_sky_–PCP7_sky_ interactions?” Each student pair generated and presented 3 hypotheses in a “group meeting”–style lecture, and then, as a team, we identified our top 8 to test (1 per pair of students; Box 2 and Fig 2B). The most common theme consisted of probing the role of specific residues at the P450_sky_–PCP7_sky_ interface by studying protein mutants. While this article focuses on results from the most popular theme, other groups worked on distinct hypotheses that required approaches more chemical and physical in nature (Box 2).

**Box 2. Student-generated hypotheses on the biochemical forces that guide functional P450_sky_–PCP7_sky_ interactions.**

- Hydrophobic and ionic interactions guide P450_sky_–PCP7_sky_ functional interactions.
  - Mutate amino acid residues involved in possible hydrophobic effects (P450_sky_: W193, L194, L200, and L239; PCP7_sky_: A45, L62, and F66) and evaluate binding by UV-vis and sedimentation velocity experiments with an analytical ultracentrifuge (SV-AUC). See main text for details.
  - Mutate relevant amino acid residues involved in ionic interactions at interface of PCP7_sky_ and P450_sky_ (PCP7_sky_: T46, R63, K47) and evaluate binding by UV-vis and SV-AUC.
- Specificity of P450_sky_ binding to PCPs is achieved because the phosphopantetheine (Ppant) arm of functional PCP partners (e.g., PCP7_sky_) gains access to the P450_sky_ heme, whereas nonfunctional partners (e.g., PCP10_sky_) do not.
  - Use SV-AUC to determine if P450_sky_ forms a complex with PCP7_sky_ and PCP10_sky_. Cyanylate the PCPs and leverage site-specific vibrational spectroscopy and UV-vis to evaluate the position of the Ppant arm of PCP7_sky_ and PCP10_sky_ in the presence of P450_sky_ [9].
- Structure of PCP7_sky_ plays an important role in the overall architecture of the protein’s tertiary structure and therefore binding affinity of P450_sky_.
  - Create PCP7_sky_ P69K mutant and evaluate tertiary structure using CD and binding to P450_sky_ by UV-vis and SV-AUC.
- The sterics/electronics of the Ppant arm or PCP-bound substrate play an important role in the selectivity of β-hydroxylation reactions catalyzed by P450_sky_.
  - Substitute native L-(OMe-Tyr) substrate for L-OMe-hydroxyphenylglycine, load onto PCP7_sky_, and evaluate binding to P450_sky_ by UV-vis and SV-AUC.
  - Synthesize Ppant arm analogs of shorter and longer length, load onto PCP7_sky_, and evaluate binding to P450_sky_ by UV-vis and SV-AUC.

Students wrote individual research proposals in the format of an “NSF Graduate Research Proposal” in which they outlined the significance of the problem based on a review of the relevant literature, their proposed experimental work, anticipated obstacles, and impact on the field. Then, as a team (students, faculty, and teaching assistants), we developed methods that leveraged the skill sets and high-level equipment available to us at our institution and that of our collaborators in order to carry out the aims of the proposals. For example, we developed an assay to determine the binding constants of P450_sky_ with PCPs using sedimentation velocity experiments with an analytical ultracentrifuge (SV-AUC), resulting in our first publication derived in part from work in this teaching laboratory [3]. Students proposed to apply this assay to quantify the effects of mutating specific residues on P450_sky_ and/or PCP7_sky_. We also employed site-specific vibrational spectroscopy and liquid chromatography mass spectrometry so that students could gain hands-on experience with additional sophisticated instruments.

**Fig 3:**
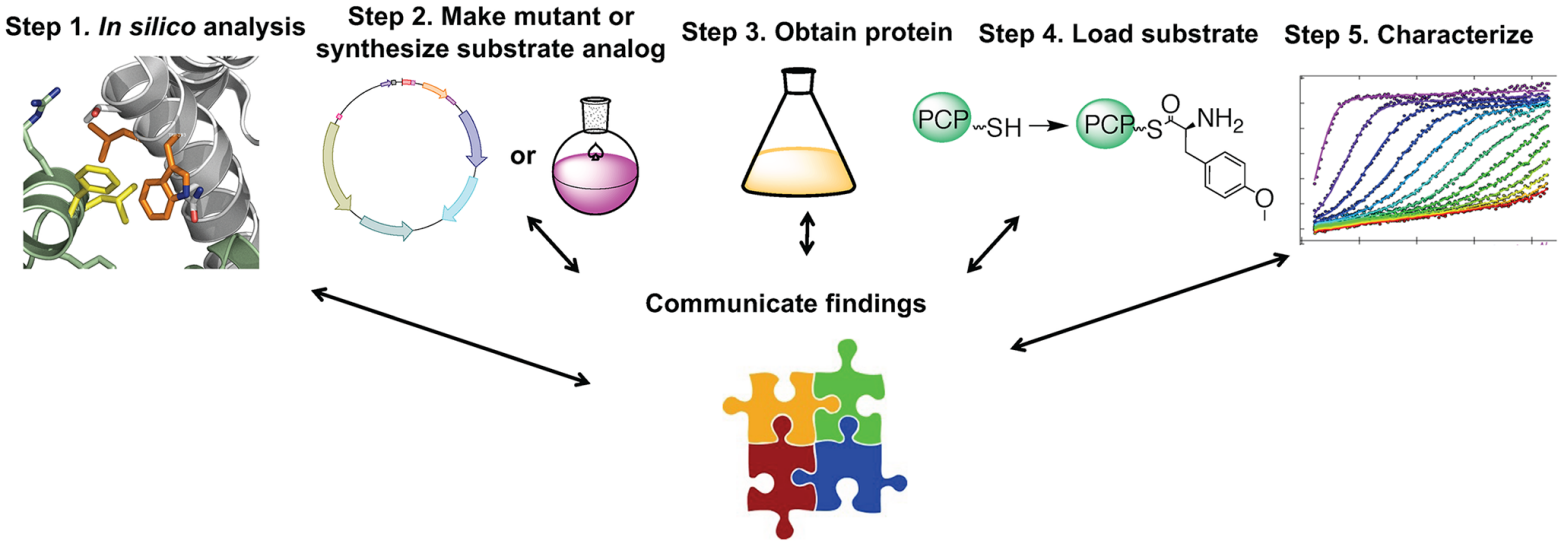
General workflow for students investigating the noncovalent interactions involved in P450_sky_-catalyzed β-hydroxylation of L-(OMe)-Tyr. This involves computational analysis (Step 1), molecular biology or synthetic chemistry (Step 2), protein purification (Step 3), chemoenzymatic assays (Step 4), and biochemical and biophysical experiments (Step 5). This workflow is a template for realizing an integrated science curriculum, as described and assessed by the Interdisciplinary Learning Consortium [9]. PCP, peptidyl carrier protein.

We next made a small library of protein mutants based on residues hypothesized to play a role in P450_sky_–PCP7_sky_ complex formation and interrogated the role of each identified residue using a workflow at the chemistry–biology interface (Figs 2 and 3, S1 Manual, and S1 Methods). Student pairs created, expressed, and purified 1 of 4 alanine mutants: W193A and L194A on P450_sky_ and L62A and F66A on PCP7_sky_ (Fig 2B, S1 Fig). Next, we used circular dichroism spectropolarimetry (CD) and SV-AUC to evaluate the effect of the mutation(s) on protein secondary structure and stability (S2, S3, S4, S7 and S8 Figs). We discussed the advantages and disadvantages of using CD to study protein stability, as measured using thermal or chemical denaturation, and the utility of comparing the changes in the free energy of individual proteins to the observed changes in binding constants with P450_sky_ in order to gain a thorough understanding of the effects of a given mutation (S1 Table). After establishing that all mutants exist in the monomeric state in solution, we evaluated the ability of PCP7_sky_ to bind P450_sky_ and access its heme core using SV-AUC. For these binding experiments, students used chemoenzymatic reactions to load either the native substrate (L-(OMe)-Tyr, see box in Fig 1) or an established synthetic imidazoyl inhibitor-type compound onto the reactive site of the PCP [3,7].

**Fig 4:**
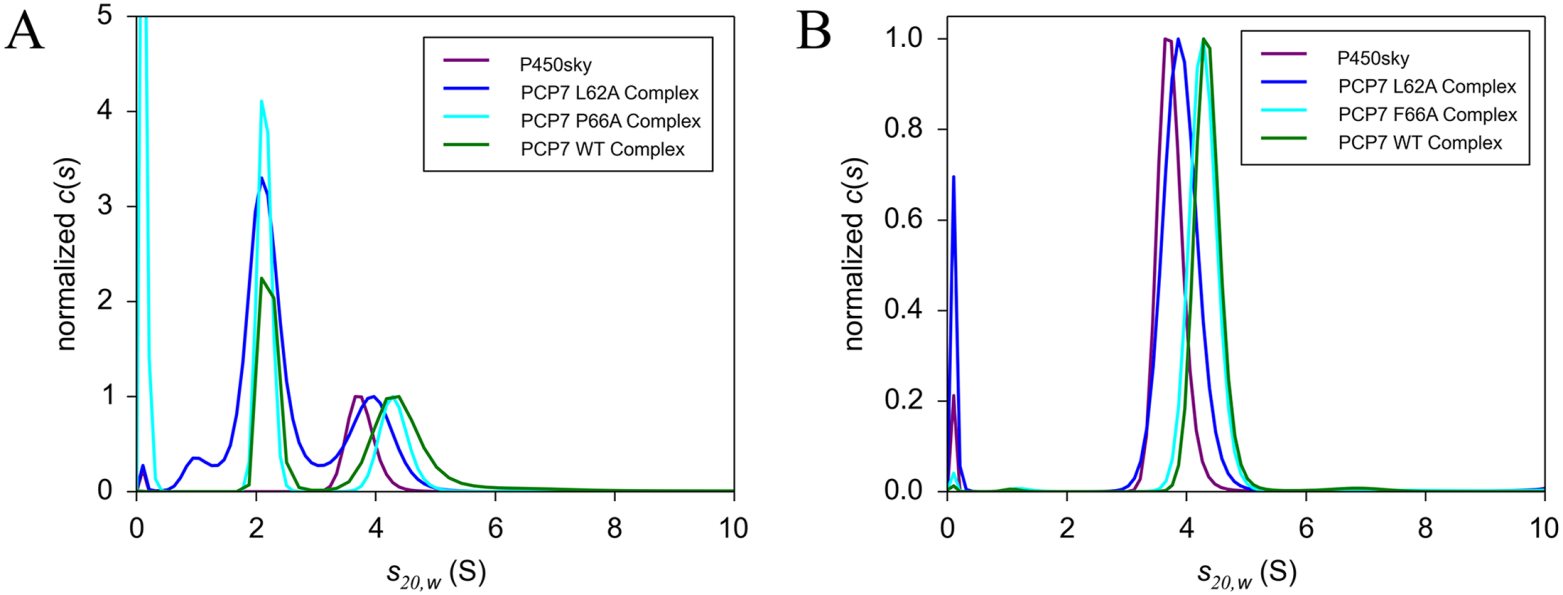
SV-AUC data collected and analyzed by students to obtain dissociation constants for P450_sky_ and mutants of P450_sky_ interacting with inhibitor-bound PCP7_sky_ (L-imidazoyl-PCP7_sky_). Comparisons of the *c(s)* distributions are shown for 10 μM P450_sky_ wild type alone and in complex with 60 μM L-imidazoyl-PCP7_sky_ L62A, L-imidazoyl-PCP7_sky_ F66A, and L-imidazoyl-PCP7_sky_ wild type. A) 280 nm (protein), B) 418 nm (heme). In general, shifts to the right suggest that the reaction boundary favors tighter binding. SV-AUC, sedimentation velocity experiments with an analytical ultracentrifuge.

We found that the W193A and L194A P450_sky_ mutants bound more weakly to imidazoyl-PCP7_sky_, which was reflected in their higher K_D_ values (S1 Table, S5 and S6 Figs). Mutating the interfacing residues on PCP7_sky_ (L62A and F66A) similarly resulted in weaker binding between imidazoyl-PCP7_sky_ with P450_sky_, with the most dramatic change observed for the L62A complex, with a K_D_ of >300 μM (Fig 4, S1 Table, and S9 Fig). To bolster these results, we obtained binding constants for the interaction of L-(OMe)-Tyr-PCP7_sky_ with the appropriate double P450_sky_ mutant (See S1 Table and S10 and S11 Figs for details).

The data acquired in this module of Biochemistry Superlab validate the original crystal structure [6] and provide a starting point for future protein engineering studies. Upon completion of the course, 1 student continued to explore the possibility of turning a nonbinding PCP (for which the cognate amino acid is not hydroxylated) into a binding PCP by building chimeras as part of an independent research project. These studies are ongoing and showcase how a teaching lab can be harnessed to inform longer-term research projects.

We also encountered our fair share of pitfalls, such as proteins crashing out of solution, failed plasmid transformations, and instruments malfunctioning. Faculty and teaching assistants consciously gave room for students to struggle—making and recovering from mistakes—and then engaged in group meetings to discuss effective strategies for troubleshooting. From these experiences, students honed troubleshooting skills and learned the value of having a good backup plan. Indeed, the hardships and obstacles faced were some of the most valuable experiences, because they inspired students to dig into the primary literature, communicate ideas to one another, devise “divide and conquer” plans to gain clues, and work as a team to compile pieces of the puzzle to move the project forward. The unexpected outcomes challenged each member of the class to develop teamwork skills. As the semester progressed, it was increasingly common for students to share the workload by some offering to stay late in return for others offering to come in early, such that more work could be accomplished by the entire class without any individual student needing to log unnecessary hours.

The benefits that stem from the originality of the exercise described above are naturally preserved in the “Biochemistry Superlab” model because the pairs of faculty teaching the course, and thus the project themes, vary from year to year. To expand the impact of the project while maintaining a class size conducive to a team atmosphere, one could run concurrent sessions on the same or a complementary theme in the future, as is already done for the Superlabs offered in the Biology Department at Haverford College.

## Win, win, win

Designing and executing a laboratory experience such as the one we describe above does not come without challenges. While barriers to implementation (such as well-documented challenges in CUREs integrated across disciplines, including logistics, staffing, cost, time investment, and discomfort with unanticipated outcomes) [8] must be overcome, we found that the experience benefited instructors, the students, and the broader biochemical community (S1 Assessment).

### A win for faculty

Integrating original research across disciplines into the classroom offers faculty the opportunity to simultaneously contribute to all 3 components of academic work: teaching, research, and service. Not only were we able to advance our own scholarly work in the form of exciting preliminary data to incorporate into publications and grant applications, but this course inspired us to establish new collaborations and foster collegial relationships across departmental cultures. As faculty at a primarily undergraduate institution, we enjoyed our newfound synergy between our teaching and research goals, a benefit of developing and teaching CUREs that is well-documented [2,8]. Further, we found that working on original research in the context of a course required us to be flexible and think on our feet, as well as to think more broadly and creatively across disciplines, which in turn made us more fully and naturally engaged with our student collaborators.

### A win for students

The opportunity for undergraduates to benefit from original research via embedding students into research groups is often limited based on the number of positions available. By incorporating original research in the biochemical teaching laboratory, more students can gain the benefits that come from research earlier on in their academic career. We have taken multiple approaches at assessing certain student and course outcomes, including assessment of student learning through clearly articulated learning goals, student self-reporting through Haverford College course surveys, and student self-reporting through the Research on the Integrated Science Curriculum (RISC) analysis, provided by an external group that surveys a broad swathe of colleges and universities in their curriculum-based research laboratories [9]. Students reflected on their experience in Biochemistry Superlab by completing the RISC survey 2 years after completion of the course (1 year after graduating from Haverford). The results (http://ww3.haverford.edu/chemistry/Charkoudian/in_the_classroom/2017%20RISC%20Survey.pdf) point to impressive student gains in biochemical research skills, as well as confidence and tolerance to obstacles. A summary of the RISC survey and the original results (courtesy of David Lopatto and Leslie Jaworski of Grinnell College) can be found in S1 Assessment.

We draw 1 comment from our internal course evaluation from a student that mirrors the inspiration that led us to propose this manuscript for publication: “While my fellow Sky-mates (was that what we called ourselves?) will agree that the class was difficult and stressful at times, I think that it taught me resilience and patience in a way that no other class at Haverford did. In fact, if everything had gone perfectly smoothly for me in Superlab, I would have learned a lot less about tolerance to failure and obstacles in research, as well as about the importance of mental flexibility to adjust to the unexpected.”

In an era in which students increasingly seek comfort in “getting the right answer” and following a pre-paved road to “success,” Biochemistry Superlab challenges our students to step into their zones of proximal discomfort. Students learn to make choices in the face of uncertainty while mastering concepts and techniques at the chemistry–biology interface (Box 1). Many of our alumni state that these skills have translated well in their respective fields, even for those outside of the sciences.

### A win for the field

The impact of incorporating original research into the classroom extends well beyond the campus walls through contributions to publications and community resources. Moreover, course alumni pursuing graduate degrees in the sciences enter into their programs with clarity of purpose, experience using state-of-the-art equipment, and solid communication and problem-solving skills. In our view, when original research is incorporated into an undergraduate laboratory course, the classroom is transformed into a fertile incubator for training future critical thinkers.

## A call to action

As more faculty experiment with rooting their laboratory courses in original research, both in the context of primarily undergraduate institutions and research institutions [2,4], it is becoming increasingly important for us to share our stories. Our stories serve as fodder for funding agencies, conferences, and journals to support and highlight the successes and challenges associated with integrating research with science laboratory courses. Nonetheless, merging themes of teaching and research remain in their infancy, and several questions remain: Will journals begin to embrace hybrid pedagogy/research articles? The mission of open-access mega journals could represent a nice fit for such manuscripts. Will institutions embrace faculty fulfilling the expectations of their teacher–scholar roles through an integrated venue? Will faculty at different institutions run concurrent or sequential biochemistry laboratory courses in order to tackle larger research questions or to replicate one another’s findings? The answers to these questions will directly impact how faculty and undergraduates collaborate to contribute to the future of education and research in the biochemical sciences.

## Supporting information

S1 Fig SDS-PAGE analysis of P450_sky_ and PCP7_sky_ wild-type proteins and mutants used in this study shows a greater than 90% purity after affinity column purification. PCP7_sky_ wild-type and mutants show presence of some dimer. Lanes: (1 & 6): Protein ladder (Precision Plus Protein Standards; Bio-Rad catalog # 161–0373); (2) P450_sky_ wild type; (3) P450_sky_ mutant W193A; (4) P450_sky_ mutant L194A; (5) P450_sky_ double mutant W193A/L194A; (7) PCP7_sky_ wild type; (8) PCP7_sky_ mutant L62A; and (9) PCP7_sky_ mutant F66A. Note: For lanes 7–9, the approximately 50 kDa band represents the PCP dimer. A total of 1 μg of protein was loaded in each lane and the gel was run for 90 minutes at 120V. PCP, peptidyl carrier protein.
(TIF)

S2 Fig Circular dichroism spectra and residual plots for wild type and mutants of P450_sky_ and PCP7_sky_ indicate no overall changes in tertiary structure as a result of site-directed mutations. CD spectra (top) and residuals (bottom) of (A) P450_sky_ and (B) PCP7_sky_ wild-type and mutant proteins in 10 mM phosphate, pH 7.5. CD, circular dichroism spectropolarimetry; PCP, peptidyl carrier protein.
(TIF)

S3 Fig P450_sky_ wild type and mutants sedimented as monomers under assay conditions. (A) *c(s)* distributions for data collected at 280 nm to monitor aromatic side chains. (B) *c(s)* distributions for data collected at 418 nm to monitor heme group absorbance. All proteins were prepared at 10 μM concentration and dialyzed overnight at 4°C against Sfp buffer (50 mM Tris-HCl, pH 7.4 and 10 mM MgCl_2_). Runs were carried out at 42,000 rpm and 20°C. The absorbance boundaries, fits, and residuals are shown in S4 Fig.
(TIF)

S4 Fig Raw sedimentation velocity data, fits, and residuals for P450_sky_ wild type and mutants collected at 418 nm over time. The absorbance boundary fits and residuals are shown for (A) P450_sky_ wild type; (B) P450_sky_ W193A; (C) P450_sky_ L194A; and (D) P450_sky_ W193A/L194A. Details about the conditions are described in the legend for S3 Fig. The y-axes represent absorbance at 418 nm, while the x-axes represent the distance from the center of axis of rotation in cm. Going from left to right, each scan represents the absorbance boundary at a given time. In later scans, most of the material has cleared the meniscus, and thus no absorbance is observed.
(TIF)

S5 Fig P450_sky_ double mutant W193A/L194A shows no significant binding to L-imidazoyl-PCP7_sky_ (see green line). *c(s)* distributions are shown for 10 μM P450_sky_ wild type and mutants W193A, L194A, and W193A/L194A in the presence of 60 μM L-imidazoyl-PCP7_sky_. (A) *c(s)* distributions for data collected at 280 nm. (B) *c(s)* distributions for data collected at 418 nm. PCP7_sky_ mutant proteins were dialyzed extensively overnight against Sfp buffer to remove excessive substrate. The absorbance boundaries, fits, and residuals are shown in S6 Fig. The apparent sedimentation coefficient of P450_sky_ W193A/L194A in the presence of 60 μM L-imidazoyl-PCP7_sky_ is the same as P450_sky_ in absence of the binding partner (See S1 Table). Calculated dissociation constant values obtained from sedimentation velocity data show that, as expected, introduction of W193A and L194A mutations had the biggest impact on binding, followed by W193A and L194A mutations, showing successively tighter binding. PCP, peptidyl carrier protein.
(TIF)

S6 Fig Raw sedimentation velocity data, fits, and residuals for binding of L-imidazoyl-PCP7_sky_ to P450_sky_ wild-type and mutant proteins. (A) Wild-type; (B) L193A; (C) W194A; and (D) W193A/L194A; Data were collected at 418 nm over time with fits and residuals. P450_sky_-PCP7_sky_-inhibitor complex boundary data fitted to an A+B ↔ AB model implemented in Sedphat. Details about the conditions are described in S5 Fig legend. PCP, peptidyl carrier protein.
(TIF)

S7 Fig PCP7_sky_ F66A sediments as a monomer in solution under assay conditions. Absorbance boundary fits, residuals, and *c(s)* distributions for the imidazoyl-PCP7_sky_ F66A mutant. (A–C) The 280 nm absorbance boundary fits and residuals, with protein concentrations at 20, 40, and 60 μM; (D) the corresponding *c(s)* distributions. A 3-fold concentration range was tested, and the sedimentation coefficient profiles (D) show a single peak corresponding to the monomeric form of the protein. Only the area under the peak, not the peak position, changed with increasing concentration. All samples were dialyzed overnight at 4°C against Sfp buffer. The rotor was run at 50,000 rpm. PCP, peptidyl carrier protein.
(TIF)

S8 Fig PCP7_sky_ L62A sediments as a monomer in solution under assay conditions. Absorbance boundary fits, residuals, and *c(s)* distributions for the imidazoyl-PCP7_sky_ L62A mutant. (A–C) The 280 nm absorbance boundary fits and residuals, with protein concentrations at 20, 40, and 60 μM; (D) the corresponding c(s) distributions. A 3-fold concentration range was tested and the sedimentation coefficient profiles (D) show a single peak corresponding to the monomeric form of the protein. Only the area under the peak, not the peak position, changed with the increasing concentration. All samples were dialyzed overnight at 4°C against Sfp buffer. The rotor was run at 50,000 rpm. PCP, peptidyl carrier protein.
(TIF)

S9 Fig Raw sedimentation velocity data of wild-type and mutant L-imidazoyl-PCP7_sky_ proteins with wild-type P450_sky_. The absorbance boundary fits and residuals are shown for (A) P450_sky_ wild type alone; (B) in complex with L-imidazoyl-PCP7_sky_ L62A; (C) in complex with L-imidazoyl-PCP7_sky_ F66A; and (D) in complex with L-imidazoyl-PCP7_sky_ wild type. The y-axes represent absorbance at 418 nm, while the x-axes represent the distance from the center of axis of rotation in cm. Going from left to right, each scan represents the absorbance boundary at a given time. In later scans most of the material has cleared the meniscus, and thus no absorbance is observed. Data were fitted to an A + B ↔ AB model. Details about the conditions are described in Fig 4. PCP, peptidyl carrier protein.
(TIF)

S10 Fig P450_sky_ binding site mutations abolish binding to natural substrate L-(OMe)-Tyr-PCP7_sky_. *c(s)* distributions shown for 10 μM P450_sky_ wild type and mutants W193A, L194A, and W193A/L194A in the presence of 60 μM L-(OMe)-Tyr-PCP7_sky_. (A) 280 nm data; (B) 418 nm data. Modified PCP7_sky_ was dialyzed extensively overnight against Sfp buffer at 4°C to remove excessive substrate L-(OMe)-Tyr. Absorbance boundary fits, and residuals are shown in S11 Fig. A lower affinity of P450_sky_ wild type for its natural substrate L-(OMe)-Tyr-PCP7_sky_ compared to L-imidazoyl-PCP7_sky_ was previously observed using UV-vis spectroscopy and sedimentation velocity experiments. PCP, peptidyl carrier protein.
(TIF)

S11 Fig Raw sedimentation velocity data of L-(OMe)-Tyr-PCP7_sky_ in complex with P450_sky_. The absorbance boundary fits and residuals are shown for (A) P450_sky_ wild type; (B) P450_sky_ W193A; (C) P450_sky_ L194A; and (D) P450_sky_ W193A/L194A, in the presence of 60 μM L-(OMe)-Tyr-PCP7_sky_. The y-axes represent absorbance at 418 nm, while the x-axes represent the distance from the center of axis of rotation, in cm. Going from left to right, each scan represents the absorbance boundary at a given time. In later scans most of the material has cleared the meniscus, thus no absorbance is observed. Data were fitted to an A + B ↔ AB model. Details about the conditions are described in S10 Fig legend. PCP, peptidyl carrier protein.
(TIF)

S1 Table Summary of parameters* determined from sedimentation velocity experiments. (XLSX)

S1 Methods Detailed methods and procedures for all experiments. (DOCX)

S1 Manual Laboratory manual and protocols used by undergraduate students to design and execute experiments. (DOCX)

S1 Assessment Course evaluations and RISC assessment results. RISC, Research on the Integrated Science Curriculum.
(DOCX)

## Abbreviations

AAAS: American Association of the Advancement of Science
CD: circular dichroism spectropolarimetry
CUREs: course-based undergraduate research experiences
NSF: National Science Foundation
PCPs: peptidyl carrier proteins
Ppant: phosphopantetheine
RISC: Research on the Integrated Science Curriculum
SV-AUC: sedimentation velocity experiments with an analytical ultracentrifuge
