# Supplementary figures and images for: Uncovering protein–protein interactions through a team-based undergraduate biochemistry course
Source: PLoS Biol. 2017 Nov 1;15(11):e2003145. doi: 10.1371/journal.pbio.2003145 (PMC5683658; doi:10.1371/journal.pbio.2003145)

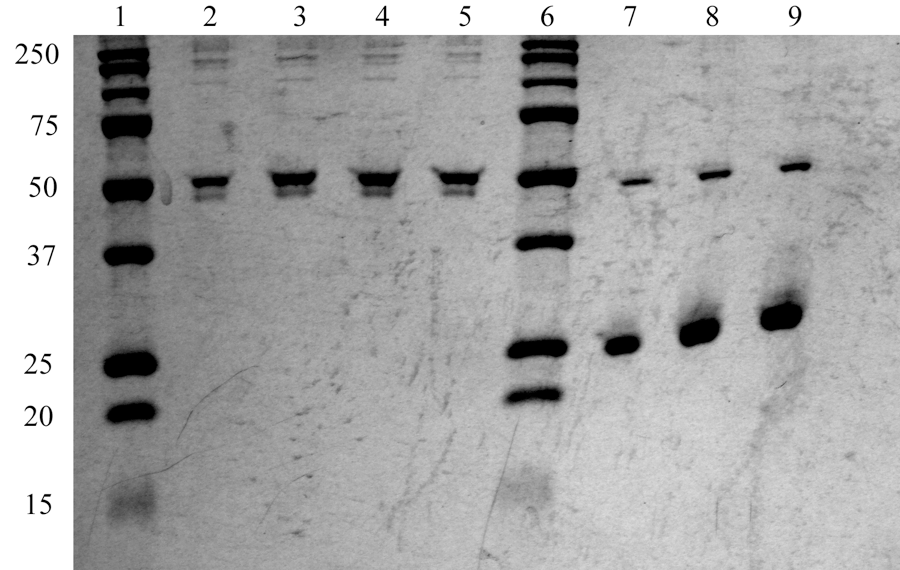

Supplement: S1 Fig — Lanes: (1 & 6): Protein ladder (Precision Plus Protein Standards; Bio-Rad catalog # 161–0373); (2) P450sky wild type; (3) P450sky mutant W193A; (4) P450sky mutant L194A; (5) P450sky double mutant W193A/L194A; (7) PCP7sky wild type; (8) PCP7sky mutant L62A; and (9) PCP7sky mutant F66A. Note: For lanes 7–9, the approximately 50 kDa band represents the PCP dimer. A total of 1 μg of protein was loaded in each lane and the gel was run for 90 minutes at 120V. PCP, peptidyl carrier protein. (TIF) [file pbio.2003145.s001.tif]

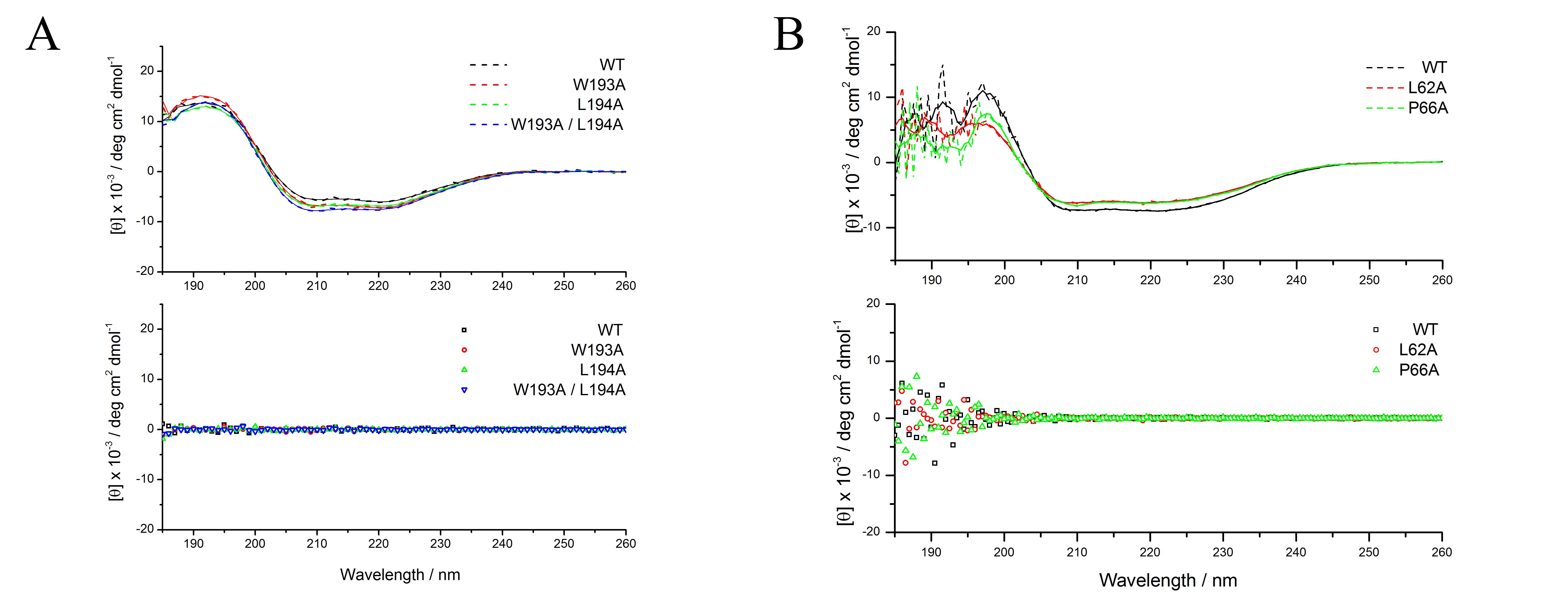

Supplement: S2 Fig — CD spectra (top) and residuals (bottom) of (A) P450sky and (B) PCP7sky wild-type and mutant proteins in 10 mM phosphate, pH 7.5. CD, circular dichroism spectropolarimetry; PCP, peptidyl carrier protein. (TIF) [file pbio.2003145.s002.tif]

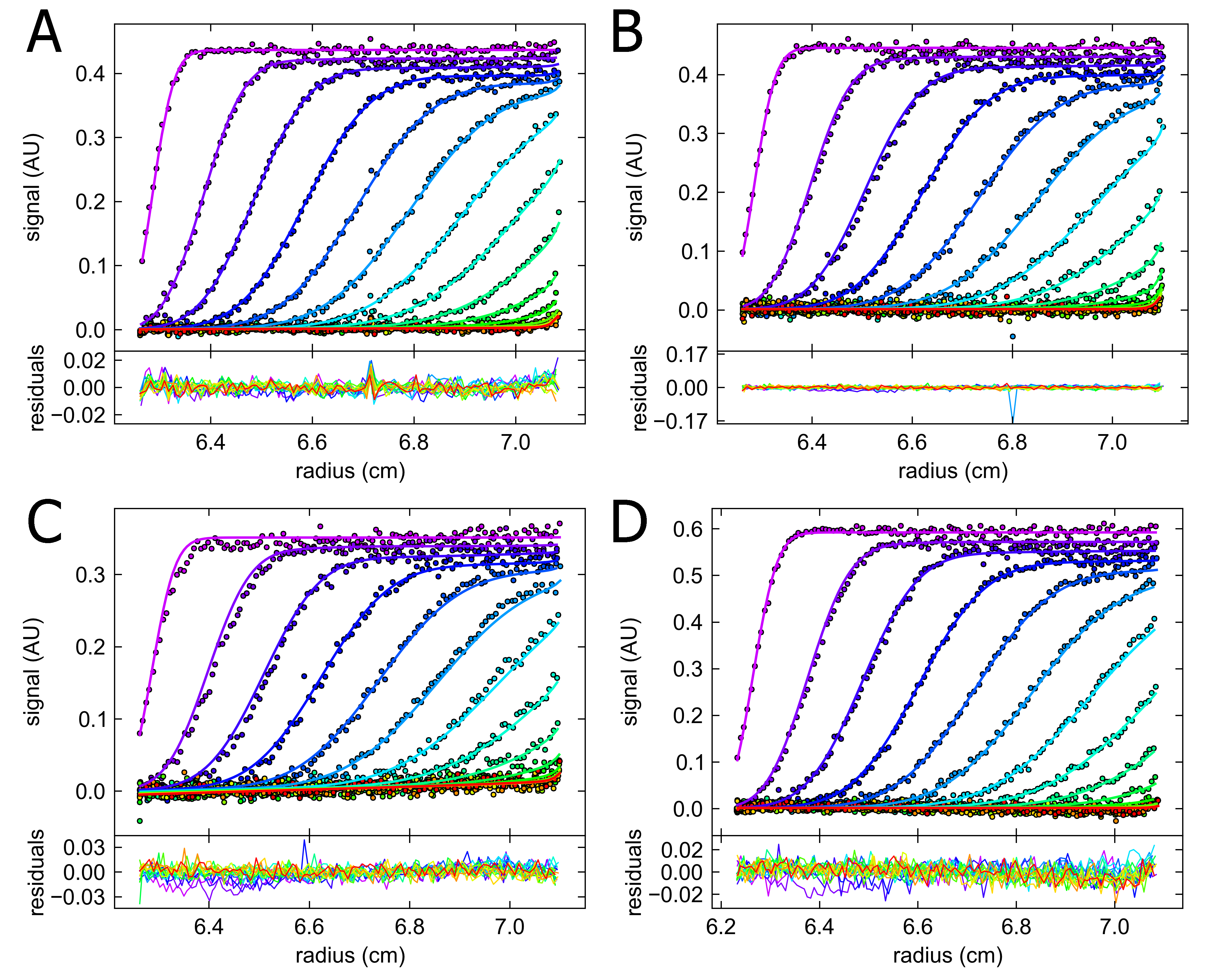

Supplement: S4 Fig — The absorbance boundary fits and residuals are shown for (A) P450sky wild type; (B) P450sky W193A; (C) P450sky L194A; and (D) P450sky W193A/L194A. Details about the conditions are described in the legend for S3 Fig. The y-axes represent absorbance at 418 nm, while the x-axes represent the distance from the center of axis of rotation in cm. Going from left to right, each scan represents the absorbance boundary at a given time. In later scans, most of the material has cleared the meniscus, and thus no absorbance is observed. (TIF) [file pbio.2003145.s004.tif]

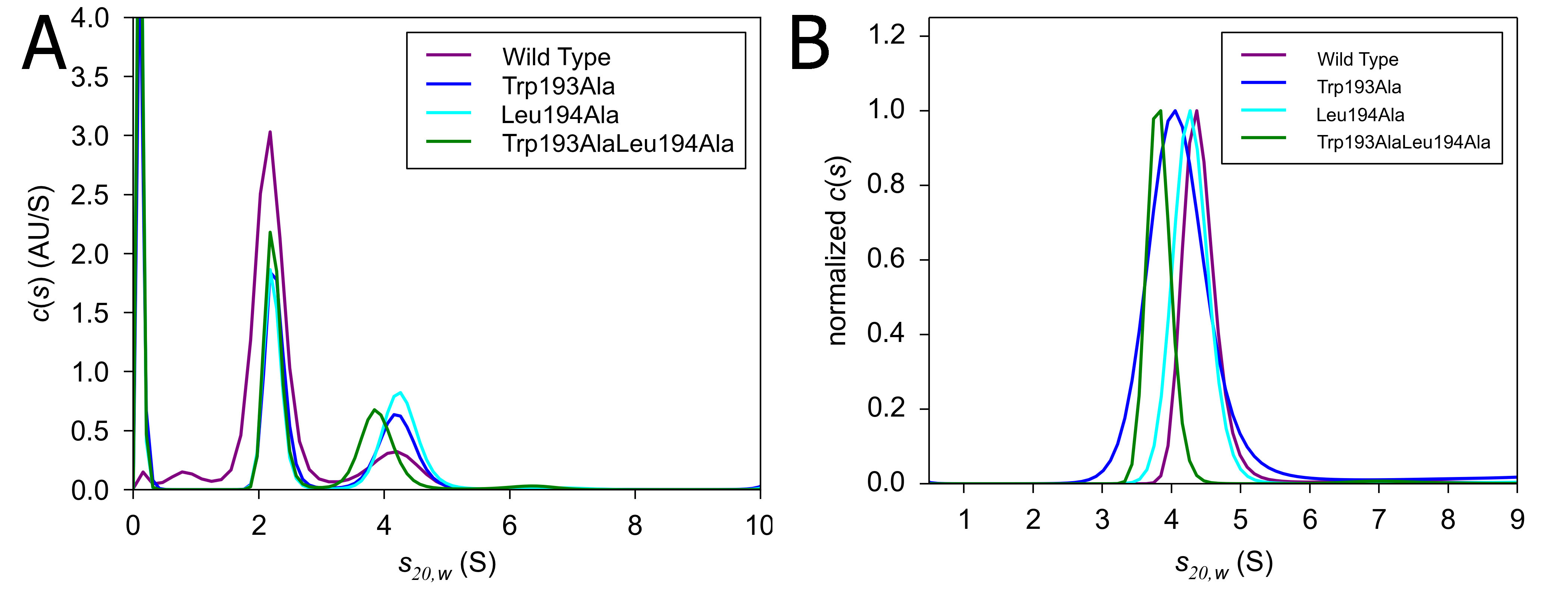

Supplement: S5 Fig — c(s) distributions are shown for 10 μM P450sky wild type and mutants W193A, L194A, and W193A/L194A in the presence of 60 μM L-imidazoyl-PCP7sky. (A) c(s) distributions for data collected at 280 nm. (B) c(s) distributions for data collected at 418 nm. PCP7sky mutant proteins were dialyzed extensively overnight against Sfp buffer to remove excessive substrate. The absorbance boundaries, fits, and residuals are shown in S6 Fig. The apparent sedimentation coefficient of P450sky W193A/L194A in the presence of 60 μM L-imidazoyl-PCP7sky is the same as P450sky in absence of the binding partner (See S1 Table). Calculated dissociation constant values obtained from sedimentation velocity data show that, as expected, introduction of W193A and L194A mutations had the biggest impact on binding, followed by W193A and L194A mutations, showing successively tighter binding. PCP, peptidyl carrier protein. (TIF) [file pbio.2003145.s005.tif]

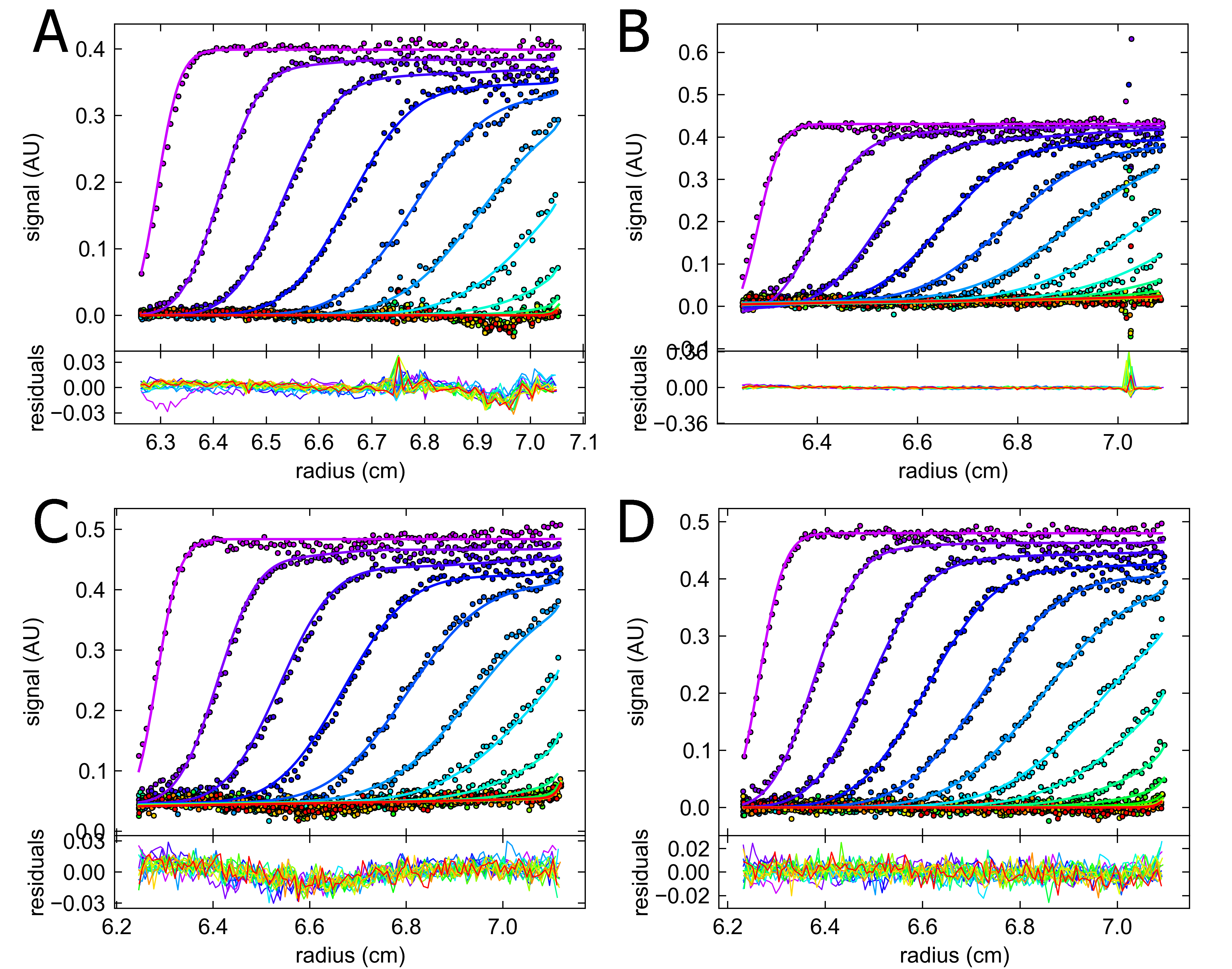

Supplement: S6 Fig — (A) Wild-type; (B) L193A; (C) W194A; and (D) W193A/L194A; Data were collected at 418 nm over time with fits and residuals. P450sky-PCP7sky-inhibitor complex boundary data fitted to an A+B ↔ AB model implemented in Sedphat. Details about the conditions are described in S5 Fig legend. PCP, peptidyl carrier protein. (TIF) [file pbio.2003145.s006.tif]

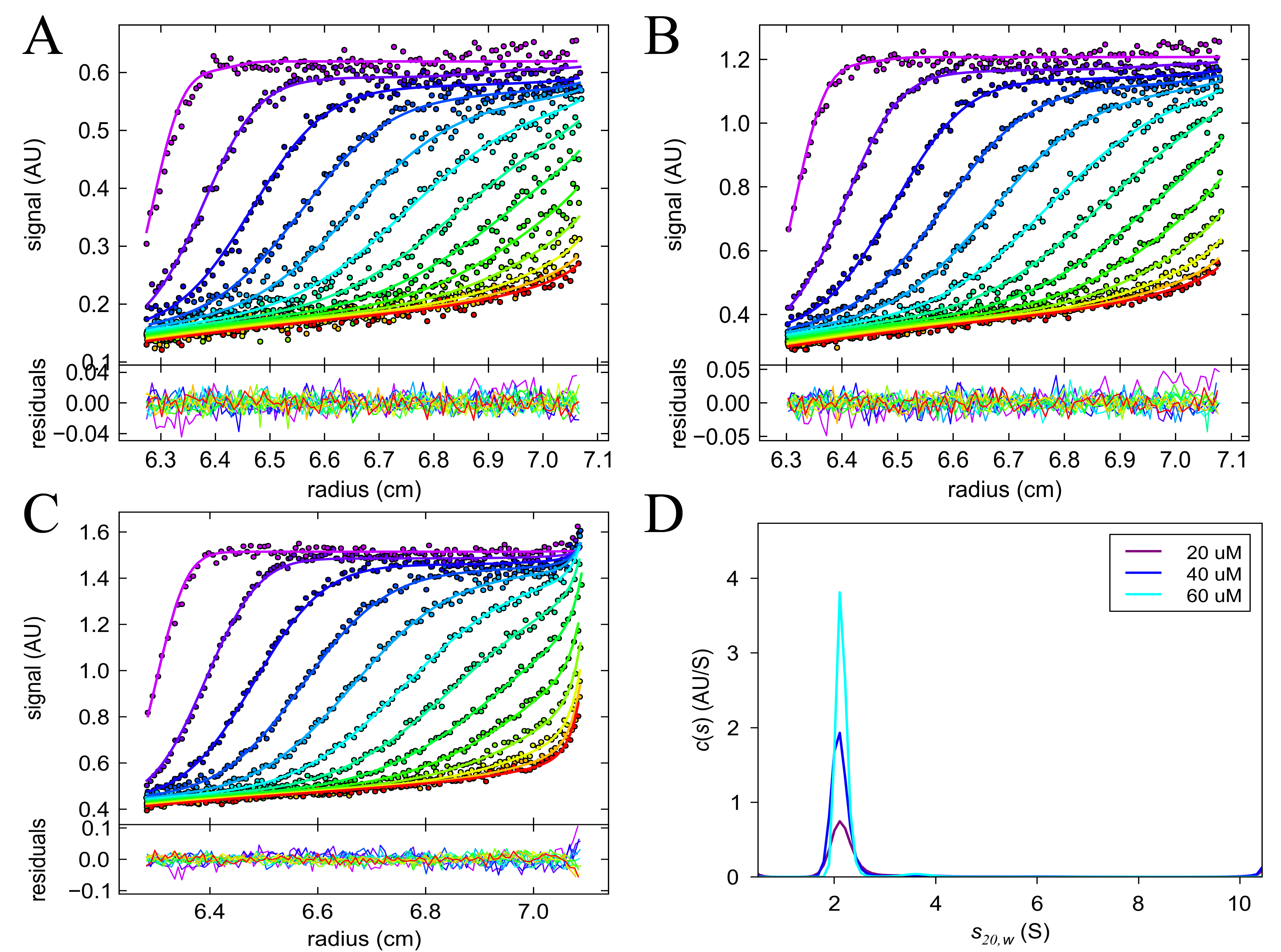

Supplement: S7 Fig — Absorbance boundary fits, residuals, and c(s) distributions for the imidazoyl-PCP7sky F66A mutant. (A–C) The 280 nm absorbance boundary fits and residuals, with protein concentrations at 20, 40, and 60 μM; (D) the corresponding c(s) distributions. A 3-fold concentration range was tested, and the sedimentation coefficient profiles (D) show a single peak corresponding to the monomeric form of the protein. Only the area under the peak, not the peak position, changed with increasing concentration. All samples were dialyzed overnight at 4°C against Sfp buffer. The rotor was run at 50,000 rpm. PCP, peptidyl carrier protein. (TIF) [file pbio.2003145.s007.tif]

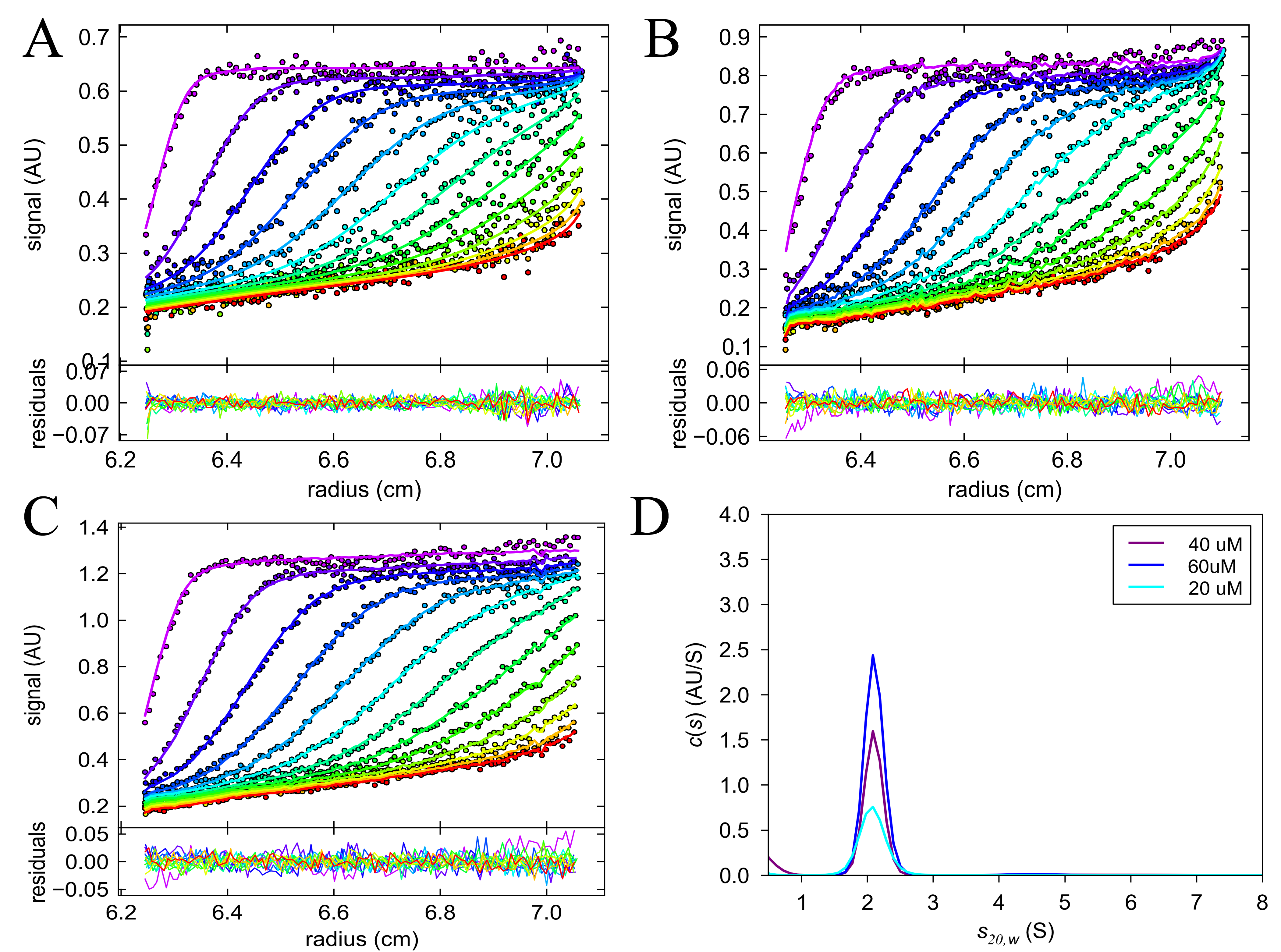

Supplement: S8 Fig — Absorbance boundary fits, residuals, and c(s) distributions for the imidazoyl-PCP7sky L62A mutant. (A–C) The 280 nm absorbance boundary fits and residuals, with protein concentrations at 20, 40, and 60 μM; (D) the corresponding c(s) distributions. A 3-fold concentration range was tested and the sedimentation coefficient profiles (D) show a single peak corresponding to the monomeric form of the protein. Only the area under the peak, not the peak position, changed with the increasing concentration. All samples were dialyzed overnight at 4°C against Sfp buffer. The rotor was run at 50,000 rpm. PCP, peptidyl carrier protein. (TIF) [file pbio.2003145.s008.tif]

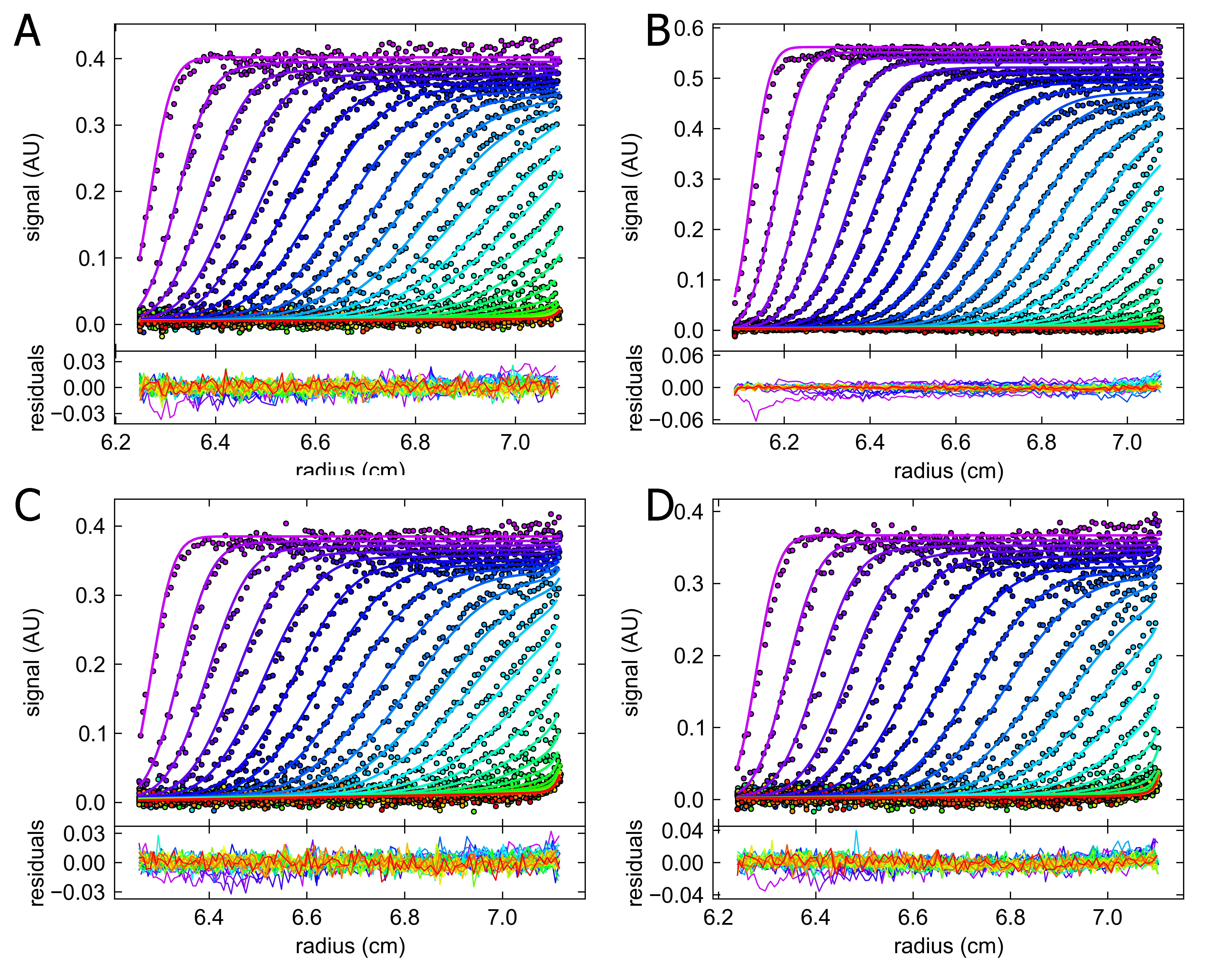

Supplement: S9 Fig — The absorbance boundary fits and residuals are shown for (A) P450sky wild type alone; (B) in complex with L-imidazoyl-PCP7sky L62A; (C) in complex with L-imidazoyl-PCP7sky F66A; and (D) in complex with L-imidazoyl-PCP7sky wild type. The y-axes represent absorbance at 418 nm, while the x-axes represent the distance from the center of axis of rotation in cm. Going from left to right, each scan represents the absorbance boundary at a given time. In later scans most of the material has cleared the meniscus, and thus no absorbance is observed. Data were fitted to an A + B ↔ AB model. Details about the conditions are described in Fig 4. PCP, peptidyl carrier protein. (TIF) [file pbio.2003145.s009.tif]

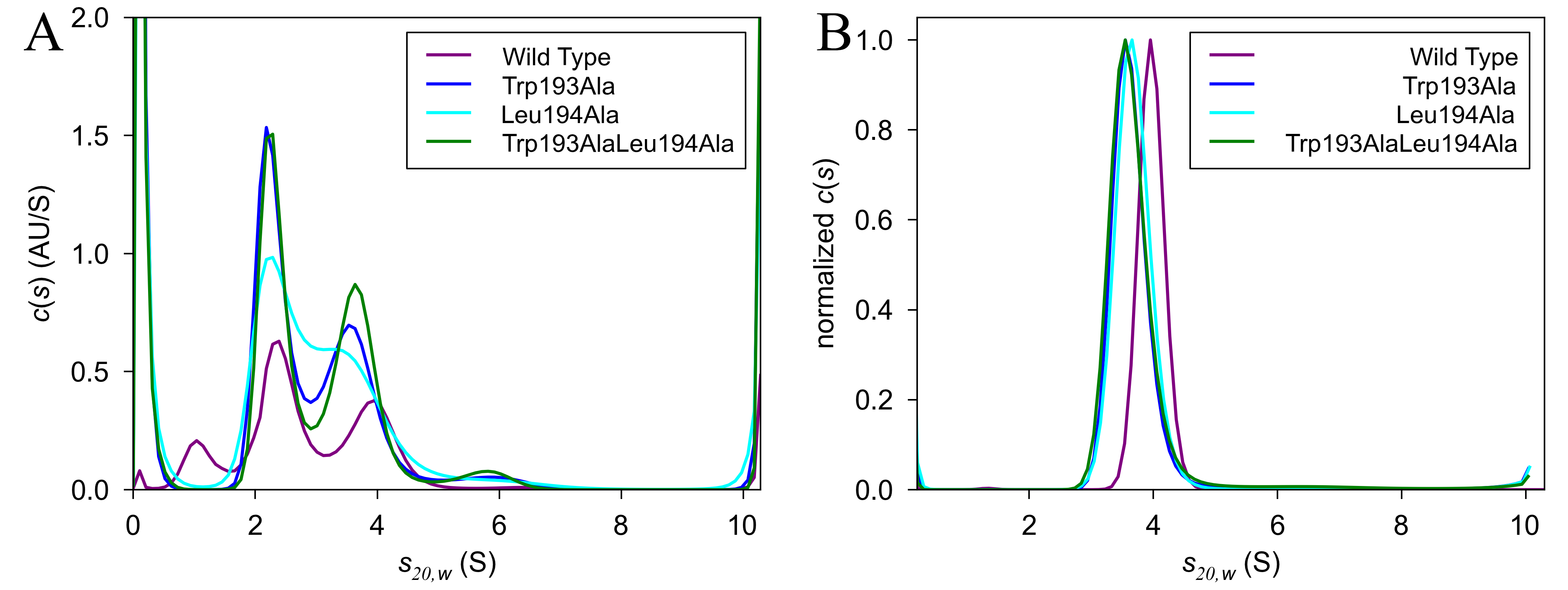

Supplement: S10 Fig — c(s) distributions shown for 10 μM P450sky wild type and mutants W193A, L194A, and W193A/L194A in the presence of 60 μM L-(OMe)-Tyr-PCP7sky. (A) 280 nm data; (B) 418 nm data. Modified PCP7sky was dialyzed extensively overnight against Sfp buffer at 4°C to remove excessive substrate L-(OMe)-Tyr. Absorbance boundary fits, and residuals are shown in S11 Fig. A lower affinity of P450sky wild type for its natural substrate L-(OMe)-Tyr-PCP7sky compared to L-imidazoyl-PCP7sky was previously observed using UV-vis spectroscopy and sedimentation velocity experiments. PCP, peptidyl carrier protein. (TIF) [file pbio.2003145.s010.tif]

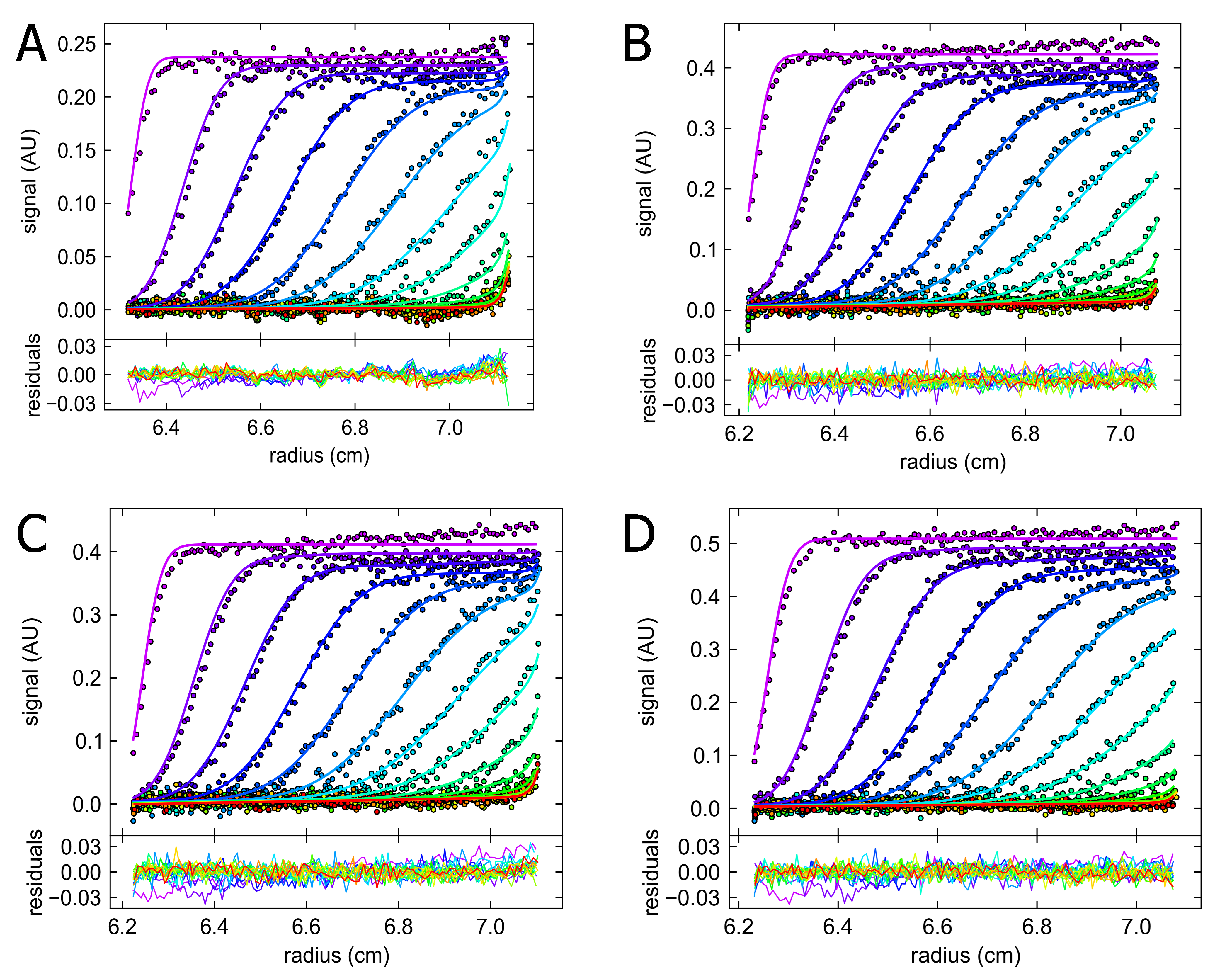

Supplement: S11 Fig — The absorbance boundary fits and residuals are shown for (A) P450sky wild type; (B) P450sky W193A; (C) P450sky L194A; and (D) P450sky W193A/L194A, in the presence of 60 μM L-(OMe)-Tyr-PCP7sky. The y-axes represent absorbance at 418 nm, while the x-axes represent the distance from the center of axis of rotation, in cm. Going from left to right, each scan represents the absorbance boundary at a given time. In later scans most of the material has cleared the meniscus, thus no absorbance is observed. Data were fitted to an A + B ↔ AB model. Details about the conditions are described in S10 Fig legend. PCP, peptidyl carrier protein. (TIF) [file pbio.2003145.s011.tif]
