# Supplementary material for: Uncovering protein–protein interactions through a team-based undergraduate biochemistry course
Source: PLoS Biol. 2017 Nov 1;15(11):e2003145. doi: 10.1371/journal.pbio.2003145 (PMC5683658; doi:10.1371/journal.pbio.2003145)
Supplement: S1 Methods — (DOCX) [file pbio.2003145.s013.docx]

**Supplementary Information for**

**Uncovering Protein-Protein Interactions through a Team-based Undergraduate Biochemistry Course**

David L. Cookmeyer^1‡^, Emily S. Winesett^1‡^, Bashkim Kokona^2^, Adam R. Huff^1^, Sabina Aliev^1‡^, Noah B. Bloch^2‡^, Joshua A. Bulos^1‡^, Irene L. Evans^1‡^, Christian R. Fagre^2‡^, Kerilyn N. Godbe^1‡^, Maryna Khromava^1‡^, Daniel M. Konstantinovsky^1‡^, Alexander E. Lafrance^2‡^, Alexandra J. Lamacki^1‡^, Robert C. Parry^1‡^, Jeanne M. Quinn^2‡^, Alana M. Thurston^1‡^, Kathleen J. S. Tsai^1‡^, Aurelio Mollo^1‡^, Max J. Cryle^3,4^, Robert Fairman^2*^, Louise K. Charkoudian^1*^

^1^Department of Chemistry, Haverford College, Haverford PA 19041, USA

^2^Department of Biology, Haverford College, Haverford PA 19041, USA

^3^The Monash Biomedical Discovery Institute, EMBL Australia, Monash University, Clayton, Victoria 3800, Australia

^4^The Department of Biochemistry and Molecular Biology and ARC Centre of Excellence in Advanced Molecular Imaging, Monash University, Clayton, Victoria 3800, Australia

^5^Undergraduate student enrolled in 2015 Biochemistry 390 (“Biochemistry Superlab”)

**Methods**

*Protein Expression and Purification*. Protocols for skyllamycin protein expression and purification were adapted from previously published procedures.(1,2) Skyllamycin PCP7_sky_ was expressed as a thioredoxin/His_6_-tag fusion protein using the expression construct pMPI-Hd05.(1,2) PCP7_sky_ was expressed in *E. coli* BL21(DE3) to obtain the *apo*-protein. P450_sky_ was expressed as a His_6_-tag fusion partner in *E. coli* BL21(DE3)/pMPI-Hd02.(1,2) Seed cultures were grown for 12 hours in 10 mL terrific broth (TB) medium containing kanamycin (50 mg/L), 0.5% (v/v) glycerol, and 0.05% (m/v) glucose at 37°C. TB medium was inoculated with 1% (v/v) of the overnight culture, incubated at 37°C, and induced with 1 mM IPTG at an OD_600_ of 0.4-0.6. Cells were grown for an additional 12 hours at 18°C. Prior to temperature reduction, cultures expressing the P450_sky_ fusion construct were supplemented with 0.5 mM δ-aminolevulinic acid to promote formation of the central heme moiety. Cells were harvested by centrifugation (9000 g, 20 min, 4°C) and resuspended in chilled lysis buffer (50 mM Tris-HCl, pH 7.8, 300 mM NaCl, 10 mM imidazole, 5 mM reduced glutathione, 5% (v/v) glycerol, and 2 mM phenylmethanesulfonyl fluoride (PMSF)). After cell lysis by sonication, cell debris was removed by centrifugation (31,000 g, 30 min, 4°C).

For His_6_-Tag purification of all proteins, high-density nickel resin (GoldBio, H-320-25) was added to the supernatant (2 mL resin/L culture) and mixed overnight at 4°C. The resin was collected using a fritted column (Bio-Rad) and washed with 6 column volumes of wash buffer (50 mM Tris-HCl, pH 7.8, 300 mM NaCl) followed by a second wash step containing 30 mM imidazole in the wash buffer. Proteins were eluted with 3 column volumes of elution buffer (50 mM Tris-HCl, pH 7.8, 300 mM NaCl, and 250 mM imidazole). Eluted fractions were mixed with glycerol to a final concentration of 10% (v/v), flash frozen in liquid nitrogen, and stored at -80°C for future use. Purity and relative molar mass of proteins were determined by SDS-PAGE (Jule, Inc) using the Mini-Protean tetra system (Bio-Rad). The theoretical mass of the thioredoxin-PCPs and P450_sky_ wild type and mutants are shown in Table 1. R4-4 Sfp, used to enzymatically attach the amino-acyl CoA moiety to the PCPs, was expressed and purified as previously reported.(4)

*SDS-PAGE Analysis*. Purity of proteins was determined using a 9% SDS-PAGE gel. A total of 1 µg purified protein was loaded into each well. The gel was run for 90 minutes at 120V. Precision Plus Protein Standards (Bio-Rad) was used for the molecular weight markers.

*Mutagenesis.* Pairs of primers ranging in size from 28-36 bp were synthesized (Eurofins Genomics) for PCR mutagenesis using the Stratagene QuickChange kit (Agilent Technologies). PCR amplification of the plasmid/primer complex was carried out using *PfuTurbo* DNA polymerase (2.5 U/μL; Agilent Technologies). After DpnI treatment of the amplified product, supercompetent XL-1 Blue cells were transformed with the modified plasmid for preparation of DNA for sequencing and subsequent transformation into BL21(DE3) cells, as appropriate. Mutations in the P450_sky_ and PCP7_sky_ genes were verified by sequencing (Eurofins Genomics). In cases where the mutation failed to work, a two-stage PCR protocol was used.(5)

*Synthesis of inhibitor imidazole-CoA and L-(OMe)-Tyr-CoA*. Coenzyme A was modified with imidazole based on the methods reported by Haslinger et al.(1) The amino acid derivatives, 4-imidazole carboxylic acid or Boc-protected OMe-L-Tyr (1.5 equiv, 48 μmol), were dissolved in 2.4 mL dimethylformamide (DMF) with *N*,*N*,*N*’,*N*’-tetramethyl-O-(1H-benzotriazol-1-yl)uranium hexafluorophosphate, (HBTU, 1.4 equiv, 44.8 μmol) and 1-hydroxybenzotriazole (HOBt, 1.4 equiv, 44.8 μmol). *N*,*N*-diisopropylamine (DIEA, 4 equiv, 128 μmol) was added, and the solution was stirred at 25°C for 10 min. Coenzyme A (1 equiv, 32 μmol) was added and the solution stirred overnight at 25°C. For the L-(OMe)-Tyr-CoA synthesis, an additional deprotection step was required in which 5.2 mL of deprotection mixture (TFA/ H_2_O/ TIPS, 95/2.5/2.5 (v/v/v)) was added and the reaction mixture stirred for an additional 2 hrs at 25°C. For both synthetic routes, the desired product was precipitated with 120 mL of ice-cold (-20 °C) ethanol and stored overnight at -20 °C. The solution was then filtered through a coarse filter, concentrated under reduced pressure, and precipitated with ethanol. The solution was then filtered using a fine porosity filter. The product was purified by preparative high performance liquid chromatography (HPLC, Rainin Model SD-200 equipped with Dynamax Absorbance Model UV-D II) using a Pursuit XRs C18-derivatized column: 10 μm bead size, 250 x 21.1 mm (imidazolyl CoA gradient: 0-10% acetonitrile (ACN) in 7 min, 10-20% ACN in 10 min, to 100% ACN in 5 min. incl. 0.1% TFA; L-(OMe)-Tyr-CoA gradient: 0-20% ACN in 7 min, 20-50% ACN in 20 min, 50-65% ACN in 10 min, to 100% in 8 min incl. 0.1% TFA). The product was analyzed by liquid chromatography / mass spectrometry (LC/MS) using an Agilent 1100 LCMS (*m/z*: imidazoyl CoA calculated [M-H]^-^ 859.6; observed: [M-H]^-^ 860.0. L-(OMe)-Tyr-CoA calculated [M-H]^-^ 943.7; observed: [M-H]^-^ 943.5). The fractions containing the desired product were pooled and lyophilized, dissolved in ddH_2_O, and stored at -80 °C for future use.

*Modification of PCP Domains*. Loading of the PCP domain with the inhibitor or substrate was performed as described previously.(2) Thawed *apo*-PCP proteins were dialyzed overnight at 4 °C in Sfp buffer (50 mM Tris-HCl, pH 7.4, and 10 mM MgCl_2_). *Apo*-PCP (80-124 µM) was mixed with 1.5 – 5 fold excess of 4-imidazoyl-CoA or L-(OMe)-Tyr-CoA substrate and 0.5-1 µM R4-4-Sfp (generously provided as a gift from the Yin Lab)^3^ in dialysis buffer. The reaction was carried out at 30 °C for 0.5-1 hour with gentle shaking and then dialyzed against 4 L of Sfp reaction buffer overnight at 4 °C in order to remove excess substrate. Successful loading of the substrate onto *apo*-PCP was verified by matrix-assisted laser desorption/ionization (MALDI) analysis, and the difference in molar masses between substrate-loaded PCP and *apo*-PCP was consistent with quantitative addition of the Ppant arm plus imidazole or the substrate L-(OMe)-Tyr. After dialysis in Sfp buffer, modified PCPs were used directly in the AUC studies.

*CD Spectropolarimetry*. All proteins (1 mL aliquots) were dialyzed against 2 L of 10 mM phosphate, pH 7.4 buffer at 4˚C in preparation for the CD runs, using 0.1-0.5 ml molecular weight cutoff (MWCO) 10 kDa dialysis cassettes (Pierce) for the P450_sky_ proteins and 0.5-3.0 ml MWCO 3.5 kDa dialysis cassettes for the PCP7_sky_ proteins. After overnight dialysis, sample concentrations were determined by monitoring tryptophan and tyrosine absorption using the following extinction coefficients as determined at 280 nm: His_6_-Tag/P450_sky_ fusion = 55,885 M^-1^ cm^-1^ for wild type and the L194A mutant, and 50,485 M^-1^ cm^-1^ for both the W193A and W193A/L194A mutant. Thioredoxin/His_6_-Tag/*apo*-PCP7 fusion proteins extinction coefficient = 15,085 M-1 cm-1.^31^ Stock solutions were diluted in dialysis buffer to final concentrations between 5-10 µM. In order to remove large non-specific aggregates, samples were filtered using a 0.2 µm low protein-binding filter with HT Tuffryn membrane (PALL Life Sciences). CD spectra were recorded on a CD model 410 spectropolarimeter (Aviv Biomedical, Lakewood, NJ 08701) using a 0.1 cm pathlength cuvette. Ellipticity, θ, was recorded at 25˚C between 180 nm and 260 nm, with the following parameters: step size 0.5 nm, averaging time 5 sec, bandwidth 1 nm. The instrument gives raw output in ellipticity measured in millidegrees (mdeg). In order to compare data from each mutant, ellipticity, θ, was converted to molar ellipticity, [θ], with units of degrees cm^2^ dmol^-1^. The spectra were smoothed using a manual smoothing function implemented in the Aviv instrument software, using a window width of 11 data points, a polynomial degree of 2. Raw data, smoothed data, and residuals were plotted in Origin (v. 8.6.0).

*Analytical Ultracentrifugation*. All proteins were dialyzed against 4 L of Sfp reaction buffer at 4°C in preparation for the sedimentation velocity (SV) runs, using 0.1-0.5 ml MWCO 10 kDa dialysis cassettes (Pierce) for the P450_sky_ protein and 0.5-3.0 ml MWCO 3.5 kDa dialysis cassettes for the modified PCP proteins. Concentrations of stock solutions were determined by monitoring tryptophan and tyrosine absorption using the extinction coefficients used for the CD experiments. Each run consisted of 8-10 μM His_6_-Tag/P450_sky_ fusion by itself; and a mixture of 8-10 μM His-Tag/P450_sky_ fusion and 50-120 µM modified PCP7_sky_. Based on the estimated concentration and extinction coefficient of the R4-4 Sfp, the protein expected absorbance is 0.03 at 280 nm, which is comparable to noise levels.

All experiments were performed using a Beckman model Optima XL-A AUC equipped with an An-60 Ti rotor. SV runs used two-channel Epon, charcoal-filled centerpieces with 1.2-cm path lengths containing 350 µL samples and 360 µL buffer references. Sedimentation boundaries of P450_sky_ alone and in complex with PCPs were measured at a speed of 42,000 rpm at 20 °C, using a data collection step size of 0.003 cm, a delay time of 0 seconds, and a total of 100 scans. Samples were monitored at 280 nm and 418 nm with a requirement for a starting absorbance between 0.3-0.6. Sedimentation boundaries for mutant PCPs were monitored at a speed of 50,000 rpm at 20˚C, step size of 0.003 cm, a delay time of 0 seconds, and a total of 150 scans. Samples were monitored at 280 nm with a requirement for a starting absorbance between 0.3-0.6.

Temperature-corrected partial specific volumes, densities, and viscosities were calculated using Sednterp (v. 1.08).(6) The partial specific volumes for the acyl-PCP proteins were calculated using a previously established procedure.(7)

Model independent analysis for determining heterogeneity of mixtures was carried out using both DCDT+ (v.2.4.0),(8) and Sedfit (v. 14.4d).(9) DCDT+ implements the dc/dt method of analysis,(10) using a time derivative technique, taking a “snapshot” of a subset of scans (15-20%), and transforming this subset to produce a g(s*) distribution. Using this algorithm, the data were fitted for sedimentation, s_20,w_, diffusion, D_20,w_, and these parameters, along with calculated molar mass (MM) information, are reported in Table S1. SV data were also analyzed for heterogeneity using Sedfit (v. 14.4d) using a model-independent continuous sedimentation, *c(s)*, distribution function. Regularization of the distribution by the maximum entropy method was used with the parameter, α, constrained to a value of 0.95. In fitting the data, all species were assumed to have the same frictional ratio, f/f_0_. *c(s)* distributions were plotted using the Gussi (v. 1.0.3) interface implemented in Sedfit (v. 14.4d) (9,11).

Sedimentation of interacting two-component systems predicts the existence of up to three boundaries: the undisturbed sedimentation of the free species and a separate species representing the complex formed by the two species. However, our 280 nm data appear to contain only two boundaries, with one component sedimenting with the expected size of the PCP species, and a second component that can be modeled as a reaction boundary exhibiting the coupled sedimentation of a mixture of the free form of P450_sky_ and its complex with PCP. Multi-signal c_k_(s) analysis for multi-component mixtures implemented in Sedphat designed to take advantage of different extinction coefficient of sedimenting protein components (12) allowed us to determine the composition of protein complexes in the mixture and resolve multiple co-existing complexes. In multi-signal c_k_(s) analysis, constrain was applied so that all > 3.8 S species were complexes, which had at least 1:1 or higher stoichiometries (*e.g.* 2:2) .(13) Single-site heterogeneous association A + B ↔ AB model was used to determine dissociation constants of wild type and mutant proteins. Molar masses of wild type and mutant proteins were determined in separate experiments and values are reported in Table S1. For each PCP7_sky_ mutant, data were collected at multiple concentrations to determine if there were any self-association of PCP7_sky_ and in order to distinguish between the noninteracting mixtures or interacting system alternatives.

**Reference List**

1. Haslinger, K., Brieke, C., Uhlmann, S., Sieverling, L., Süssmuth, R. D., and Cryle, M. J. (2014) The Structure of a Transient Complex of a Nonribosomal Peptide Synthetase and a Cytochrome P450 Monooxygenase. *Angewandte Chemie International Edition* **53**, 8518-8522

2. Uhlmann, S., Süssmuth, R. D., and Cryle, M. J. (2013) Cytochrome P450 skyInteracts Directly with the Nonribosomal Peptide Synthetase to Generate Three Amino Acid Precursors in Skyllamycin Biosynthesis. *ACS Chem Biol* **8**, 2586-2596

3. Sunbul, M., Marshall, N. J., Zou, Y., Zhang, K., and Yin, J. (2009) Catalytic Turnover-Based Phage Selection for Engineering the Substrate Specificity of Sfp Phosphopantetheinyl Transferase. *Journal of Molecular Biology* **387**, 883-898

4. Wang, W., and Malcolm, B. A. (1999) Two-stage PCR protocol allowing introduction of multiple mutations, deletions and insertions using QuikChange Site-Directed Mutagenesis. *Biotechniques* **26**, 680-682

5. Schuck, P., and Zhao, H. (2011) Editorial for the special issue of methods &quot;Modern Analytical Ultracentrifugation&quot;. *Methods (San Diego, Calif.)* **54**, 1-3

6. Durchschlag, H., and Zipper, P. Calculation of the partial volume of organic compounds and polymers. *Progress in Collid &amp; Polymer Science* **94**, 20-39

7. Philo, J. S. (2006) Improved methods for fitting sedimentation coefficient distributions derived by time-derivative techniques. *Anal Biochem* **354**, 238-246

8. Dam, J., Velikovsky, C. A., Mariuzza, R. A., Urbanke, C., and Schuck, P. (2005) Sedimentation velocity analysis of heterogeneous protein-protein interactions: Lamm equation modeling and sedimentation coefficient distributions c(s). *Biophysical journal* **89**, 619-634

9. Stafford, W. F., 3rd. (1992) Boundary analysis in sedimentation transport experiments: a procedure for obtaining sedimentation coefficient distributions using the time derivative of the concentration profile. *Anal Biochem* **203**, 295-301

10. Zhao, H., Brautigam, C. A., Ghirlando, R., and Schuck, P. (2013) Overview of current methods in sedimentation velocity and sedimentation equilibrium analytical ultracentrifugation. *Current protocols in protein science / editorial board, John E. Coligan ... [et al.]* **Chapter 20**, Unit20.12

11. Balbo, A., Minor, K. H., Velikovsky, C. A., Mariuzza, R. A., Peterson, C. B., and Schuck, P. (2005) Studying multiprotein complexes by multisignal sedimentation velocity analytical ultracentrifugation. *Proc Natl Acad Sci U S A* **102**, 81-86

12. Kokona, B.; Winesett, E. S.; Krusenstiern, von, A. N.; Cryle, M. J.; Fairman, R.; Charkoudian, L. K. (2016) Probing the selectivity of betahydroxylation reactions in non-ribosomal peptide synthesis using analytical ultracentrifugation. *Anal. Biochem*, **495**, 42-51.
