# Supplementary material for: Uncovering protein–protein interactions through a team-based undergraduate biochemistry course
Source: PLoS Biol. 2017 Nov 1;15(11):e2003145. doi: 10.1371/journal.pbio.2003145 (PMC5683658; doi:10.1371/journal.pbio.2003145)
Supplement: S1 Manual — (DOCX) [file pbio.2003145.s014.docx]

**Supplementary Information for**

**Uncovering Protein-Protein Interactions through a Team-based Undergraduate Biochemistry Course**

David L. Cookmeyer^1‡^, Emily S. Winesett^1‡^, Bashkim Kokona^2^, Adam R. Huff^1^, Sabina Aliev^1‡^, Noah B. Bloch^2‡^, Joshua A. Bulos^1‡^, Irene L. Evans^1‡^, Christian R. Fagre^2‡^, Kerilyn N. Godbe^1‡^, Maryna Khromava^1‡^, Daniel M. Konstantinovsky^1‡^, Alexander E. Lafrance^2‡^, Alexandra J. Lamacki^1‡^, Robert C. Parry^1‡^, Jeanne M. Quinn^2‡^, Alana M. Thurston^1‡^, Kathleen J. S. Tsai^1‡^, Aurelio Mollo^1‡^, Max J. Cryle^3,4^, Robert Fairman^2*^, Louise K. Charkoudian^1*^

^1^Department of Chemistry, Haverford College, Haverford PA 19041, USA

^2^Department of Biology, Haverford College, Haverford PA 19041, USA

^3^The Monash Biomedical Discovery Institute, EMBL Australia, Monash University, Clayton, Victoria 3800, Australia

^4^The Department of Biochemistry and Molecular Biology and ARC Centre of Excellence in Advanced Molecular Imaging, Monash University, Clayton, Victoria 3800, Australia

^5^Undergraduate student enrolled in 2015 Biochemistry 390 (“Biochemistry Superlab”)

**Manual**

What drives the Interactions in the PCP-P450 Complex?

Biochemistry 390B

Spring 2015

Louise Charkoudian and Robert Fairman

| **Week Of** | **Day** | **Experiments** | **Meetings** |
| --- | --- | --- | --- |
| Jan. 20 | Tues | Lab safety, training, and orientation; introduction to Sky system, Pymol, structure analysis | Goals, expectations, syllabus, introduction to secondary metabolites and 0s |
|  | Thur | structure analysis and start work on hypothesis for next class | Journal club Pohle JACS 2011 |
| Jan. 27 | Tues | sterile technique; structure analysis; start O/N culture on Monday;  grow large scale culture (need to start in the morning),  spin down culture and freeze pellet on Wednesday | Journal club on Uhlmann et al 2013 paper, introduction and presentation of figures |
|  | Thur | cell lysis; | Journal club on Uhlmann et al 2013 paper, questions |
| Feb. 3 | Tues | protein purification;  post 2-3 hypotheses per group on Google Doc | Journal club, cont’d;  Alternate methods for busting cells, and protein purification |
|  | Thur | PAGE analysis; concentration; dialysis | Journal club on Haslinger et al 2014 paper |
| Feb. 10 | Tues | Basic characterization: CD, and UV-vis data collection | Discussion of hypotheses |
|  | Thur | Basic characterization: CD, and UV-vis data analysis cont’d | IR, LC-MS |
| Feb. 17 | Tues | Independent Projects | Hypothesis presentations |

|  | Thur | Independent Projects | Hypothesis presentations |
| --- | --- | --- | --- |
| Feb. 24 | Tues | Independent Projects  Peer evaluations of notebooks. | Loading cargo and probes onto phosphopantetheine arm; Mutagenesis |
|  | Thur | AUC and Data analysis  **Research proposals due** | AUC |
| Mar. 3 | Tues | Independent Projects | quantifying binding reactions |
|  | Thur | Independent Projects  Mid-semester evaluations | No lecture |
| Mar. 10 | Spring Break | | |
| Mar. 17 | Tues | Independent Projects | No lecture |
|  | Thurs | Independent Projects | No lecture |
| Mar. 24 | Tues | Independent Projects | Journal club: paper from Yan Kung |
|  | Thurs | Independent Projects | Guest speaker: Yan Kung (crystallography) |
| Mar. 31 | Tues | Independent Projects | Journal club: paper from Nate Snyder |
|  | Thurs | Independent Projects | Guest speaker: Nate Snyder. Using LCMS to understand complex biological processes. |
| Apr. 7 | Tues | Independent Projects | No lecture |
|  | Thurs | Independent Projects  **Wiki drafts are due** | No lecture |
| Apr. 14 | Tues | Independent Projects | Journal club: paper from Brian Conlon |
|  | Thurs | Independent Projects | No lecture |
| Apr. 21 | Tues | Independent Projects | Skype with Max |
|  | Thurs | Independent Projects  **Wikis are due** | No lecture |
| Apr. 28 | Tues | Independent Projects | Journal club: paper from Brian Conlon |
|  | Thurs | Lab clean up | Wrap-Up |
| April 30 |  | Joint superlab poster symposium with Chemistry & Biology Superlabs  8:00-9:30 PM | |
| May 1 |  | Brian Conlon seminar  4:15-5:30, Sharpless Auditorium | |

**Learning Objectives (assessment category)**

At the successful completion of the semester, students will be able to:

- Visualize molecular structure and analyze phylogeny of proteins using publically available in *silico* tools. (A)
- Design and construct a plasmid for protein expression. (A)
- Express and purify proteins using standard recombinant and biochemical techniques. (A)
- Conduct biochemical assays to determine the catalytic activity of biosynthetic enzymes. (A)
- Use state-of-the-art equipment in the science center to analyze protein structure and binding. (A)
- Locate, read and understand primary journal articles and scientific reviews. (B)
- Navigate the scientific literature to inform practical laboratory techniques (A,B)
- Work safely and efficiently as a member of a research team (A)
- Maintain a laboratory notebook (C)
- Troubleshoot unexpected experimental results (A)
- Effectively communicate scientific thoughts and results in both written and oral form (D)

Assessment

Your grade for this semester will be based on the following components:

Lab and class performance (A, D) 25%

Journal clubs (B) 10%

Lab notebook (C) 20%

Hypothesis presentation (A, D) 10%

Research proposal (A, D) 10%

Wiki entry on NRBS domains (A, D) 15%

Research presentation (A, D) 10%

General Lab Policies

You will not always be able to fit all of the procedures within the constraints of a class schedule and you will be required on occasion to perform some procedures outside of the scheduled class times, particularly when using instrumentation that is unique. Of course, you may come in at other times as you wish, but you may never be in the lab without a partner. You are expected to attend the scheduled Tuesday & Thursday lectures given at 11:30 AM as well as the lab from 1:15-4 pm unless stated otherwise by the instructor. Assignments turned in late will be penalized. You should make sure that you are properly trained before using equipment or instruments in the laboratory. Please check with the instructor or the TAs if you have any questions or are unsure, about the use of any equipment or instruments.

Lab performance

Lab performance is a measure of several factors. These include: 1) an understanding of the theory and practice underlying the methods that we will be using in the laboratory; 2) the ability to carry out an experiment properly and efficiently; 3) the ability to think independently while working together effectively as a team; 4) respect for the safety and well-being of the other students in the laboratory; and 5) good lab citizenry (e.g., reporting problems with equipment or reagents running low).

**Lab notebook**

You will be working in groups of two during this biochemistry lab. However each student must keep his or her own individual laboratory notebook, since an important aim of this laboratory is to learn how to keep complete and clearly written experimental records. Lab notebooks can be purchased in the bookstore. Always bring your lab notebook **and** your manual to lab each day. Lab notebooks must be well organized and clearly written using a ballpoint pen. DO NOT USE PENCIL, since you could be tempted to erase any entries. Your objective is to ensure that you, or someone else, will be able to turn to your notebook at any future time and, from the description you have written, repeat a procedure with the same result. To this end, it is important that you do not erase, do not use correction fluid and do not use pencil or erasable pen in your notebook. Additions and corrections should always be made in ink of a contrasting color and dated.

**The following information should be recorded in your notebook**:

**a. The date**. Technically speaking, every page should have the date in the top corner. If you work in industry, or ever apply for a patent based on your work, your lab notebook becomes a legal document and the handwritten date is part of the legal record. It is good practice to start dating the pages now.

**b. A page index**, kept at the front of your notebook and maintained as you go along.

**c. A succinct title** for each experiment.

**d. A brief summary of the goals or aims** of each experiment (including broader scope, *please include the why* as well as the what).

**e. Log of procedures used.** We would like you to write out the procedures to be used on any particular day, by hand, from this manual into your lab notebook. As you do this, imagine yourself doing each step, and think about what questions you need to ask before you do. We know that this may feel redundant, but in fact, many professional scientists do just this. It will ensure that you have a day-by-day, step-by-step record of exactly what you did in lab. If you repeat a procedure later, you may simply record it by referring to the previous date and notebook page that the same experiment was performed. **Please be careful to annotate any deviations from the protocol and to carefully record precise amounts, dilutions etc of reagents actually used.** (This will be a critical component of our grading scheme.)

**f. All data, calculations and results obtained** at the time you obtain them. If you realize at a later date that you need to correct an entry made previously, add the correction and date it appropriately.

**g. Comments on significant results, explanations for unexpected findings, etc** (another critical component of our grading scheme). These notes should be written while data are still fresh in your mind (don’t wait until after dinner!).

**h. Appropriate attribution**. It will sometimes happen that one lab group will have difficulty with a particular experiment and may then obtain a reagent or even a set of results from another group. On other occasions, different groups each contributing part of an experiment will need to share data. These situations reflect what happens during collaborative science in the real world. It is therefore terribly important that you learn now to credit the people involved directly in your book. Complete and accurate citation is an important manifestation of your scientific integrity.

**i. A brief and concise discussion of your data.** At the end of each set of experiments, you need to briefly note down the answers to such questions as:

- - What did your experiments show?
  - Was the result what you expected? If so, why; if not, why not?
  - What problems did you encounter in doing the experiments?
  - How would you alter the protocol for next time?

Please note, you should **never** be embarrassed to show your own data. A careful, thoughtful discussion of ambiguous or negative results is worth more in your book than a sloppy treatment of nice data. Consider your notebook to be your scientific diary.

**You must never record your data on scraps of paper or paper towels and rewrite later!!** This wastes your time, results in needless mistakes and, most importantly, is regarded as poor, and potentially dishonest, scientific practice.

**j. Print outs of your data.** During your experiments, you will accrue digital images that should be placed in a folder on temp storage. It is your responsibility to back up important files either onto your own hard drive (via a portable flash drive) or onto the Storage Server. The locations of these files should be referenced in your notebook. Hard copies of this information should be printed out, entered and annotated in your lab notebook **as the experiments proceed**. At the end of the quarter, we will ask you to submit some electronic images. Please do not wait until the quarter is over to print out or assemble your data.

**Grading of lab notebooks:**

Here is the form that we will use to grade your lab notebooks. In order to provide you with feedback on your notebook skills, we will have an informal evaluation in the third week carried out by your colleagues. You will swap notebooks with your colleagues and have them evaluate them according to the criteria below. If you wish additional feedback, we would be happy to discuss your notebook and student evaluation on an individual basis.

 1 - Poor

2 - Fair

3 - Good

4 - Very Good

1) Organization

a) Table of Contents 1 2 3 4

b) Dates 1 2 3 4

c) Titles 1 2 3 4

2) Goals 1 2 3 4

3) Results 1 2 3 4

4) Interpretation 1 2 3 4

5) Annotation of Protocols 1 2 3 4

6) Graphs & Figures 1 2 3 4

7) Usability 1 2 3 4

**Journal Clubs**

Scientific inquiry is best accompanied by close and critical reading of the scientific literature. There will be six journal clubs this semester (three each quarter) aimed at introducing you to the body of literature relevant to this exciting field and helping you to think about the types of approaches used and the broader implications of your research. Journal clubs will also give you an opportunity to critically analyze the primary literature. Collectively, these goals will serve as a good foundation for your written research proposal and final presentation.

Journal clubs will be held in the Tuesday/Thursday lecture slots as an informal collective presentation and discussion of the article. We will provide a list of discussion questions in advance of the journal club meeting and you will be randomly assigned questions to present, explicate, or answer the day of the journal club. Therefore, we highly recommend that you answer all the questions before coming to class.

The following papers will be assigned:

1. Stefan Pohle, Christian Appelt, Mallorie Roux, Hans-Peter Fiedler, and Roderich D. Süssmuth. Biosynthetic Gene Cluster of the Non-ribosomally Synthesized Cyclodepsipeptide Skyllamycin: Deciphering Unprecedented Ways of Unusual Hydroxylation Reactions. *J. Am. Chem. Soc.* 2011, **133:**6194–6205.
2. Stefanie Uhlmann, Roderich D. Süssmuth, and Max J. Cryle. Cytochrome P450_sky_ Interacts Directly with the Nonribosomal Peptide Synthetase to Generate Three Amino Acid Precursors in Skyllamycin Biosynthesis. *ACS Chem. Biol.* 2013, **8**:2586−2596.
3. Kristina Haslinger, Clara Brieke, Stefanie Uhlmann, Lina Sieverling, Roderich D. Süssmuth, and Max J. Cryle. The Structure of a Transient Complex of a Nonribosomal Peptide Synthetase and a Cytochrome P450 Monooxygenase. *Angew. Chem. Int. Ed.* 2014, **53:**1–6.
4. Losee L. Ling1, Tanja Schneider, Aaron J. Peoples, Amy L. Spoering, Ina Engels, Brian P. Conlon, Anna Mueller, Till F. Schäberle, Dallas E. Hughes, Slava Epstein, Michael Jones, Linos Lazarides, Victoria A. Steadman, Douglas R. Cohen, Cintia R. Felix, K. Ashley Fetterman, William P. Millett, Anthony G. Nitti, Ashley M. Zullo, Chao Chen & Kim Lewis. A new antibiotic kills pathogens without detectable resistance. *Nature* 2015, doi: 10.1038/nature14098. [Epub ahead of print]

**Hypothesis Presentation & Research Proposal**

You will be writing a short research proposal describing your hypothesis, and how you aim to test your hypothesis using the various biochemical and biophysical methods that will be available. Your hypothesis will be centered around the protein structural analysis that you did, along with the proposed amino acid mutation or phosphopantetheine modification. You should also consult with the other students in the lab to know what hypotheses they are planning. Place your proposed hypotheses in the context of the broader set of hypotheses being proposed by the other student projects. As part of this assignment, you will also present (in pairs) your proposal orally to the class. You may use Powerpoint for this presentation. Presentations will be approximately 10 minutes long, and 2-3 minutes for questions.

The research proposal papers are expected to be individual efforts. The format of your paper should be as follows:

1. Hypothesis – based on structure/function analysis and mutation /phosphopantetheine modification proposed

2. Background to show your understanding of the goals of the lab and to defend your hypothesis (drawn from the literature)

3. Comparison to other plans in the lab

4. Brief description of planned experiments to test your hypothesis

5. References (with titles) at the end of your proposal entitled "References". Be sure to use proper citations within the text to your reference list (either using numbers or the standard citation). You can follow the ACS Biochemistry guidelines for reference formats. Here is an example to help guide you:

Kar, K., Jayaraman, M., Sahoo, B., Kodali, R., and Wetzel, R. (2011) Critical nucleus size for disease-related polyglutamine aggregation is repeat-length dependent. Nat. Struct. Mol. Biol. 18, 328−336.

Your proposal should be written in the style of a National Science Foundation fellowship proposal (some of you might wind up applying for one of these in the next couple of years!). These are typically two pages long and single-spaced, but we will allow a range of 2-3 pages for this assignment. You may use images to help support your proposal. Here is a brief description of the research statement requirements for NSF proposals to help guide you:

The [research] statements must be written using the following guidelines: standard 8.5" x 11" page size 12-point, Times New Roman font or Computer Modem (LaTeX) font 10-point font may be used for references, footnotes, figure captions and text within figures 1" margins on all sides single spaced or greater line spacing. The maximum length of the Graduate Research Plan Statement is two pages. These page limits include all references, citations, charts, figures, images, and lists of publications and presentations.

You should use PubMed to help you find the necessary background papers. If you are not familiar with how to do PubMed searches, you should seek out the professors or our science librarian, to help you. You should feel free to show us the articles that you have been reading for feedback.

**Grading of Research Proposals (25 pts total)**

Abstract: 3 pts

- Concise
- Captures most important points

Background: 6 pts

- Evidence of understanding of primary literature with appropriate citation of seminal work and milestones. - 2 pts
- Clear articulation of hypothesis/question - 2 pts
- Clear explanation of proposed plan of attack - 2 pts

Experimental plan: 6 pts

- Clarity in presentation of methods (right amount of detail) - 3 pts
- Explanation for why these methods are useful to address questions - 3 pts

Significance and importance: 4 pts

- Will the proposed research afford important results? – 2 pt
- Proposed research placed in the context of the class goals/experiments and the field in general – 2 pt

Clarity and organization: 2 pts

Grammar and syntax: 2 pts

Reference list: 2 pts

- Appropriate format – 1 pt
- Adequate citations included – 1 pt

**Grading of hypothesis presentations (100 pts total)**

Breadth and depth of knowledge (20 pts)

- Background information
- Proposed Techniques
- Importance of Projects

Articulation of hypothesis or question (40 pts)

- “Big picture”
- Specific aims
- Expected results

Effective communication/delivery (10 pts)

- Slide design/transitions
- Illustrations/graphics
- Pace
- Eye contact

Engagement and answers (20 pts)

- Poised, succinct answers
- Thoughtful analysis/predictions

Overall (10 pts)

- Strengths of presentation
- Quality of suggested experiments

**Wiki on NRPS domains** (with due credit to Jon Wilson for pioneering the use of Wikis)

Posts should include the following:

1. Catalytic function of the domain. Show the overall transformation as well as the detailed curved arrow mechanism.
2. Evolution of domain (if known)
3. Size and conserved sequence motifs
4. Information about the protein’s secondary, tertiary, and quaternary structure. Is it normally expressed in a module or as a standalone protein? If within a module, can it be excised and expressed as an active standalone protein? Reference any known crystal and NMR structures here.
5. Sequence-structure-function relationship.
6. Prospects for engineering to make “unnatural” natural products.

You should also include fun facts about how/when the domain was discovered, any interesting examples of the domain, etc.

Please make sure to cite all your references. The Finkling 2004 review is a good place to start.

All students are expected to provide feedback and comments on entries after drafts have been mounted (due April 9), and these comments will be a component of the grade for this assignment. Students should respond to these posts in the completion of their Wiki entry by the April 23 deadline.

**Final Research Presentation**

An important part of scientific research is presenting *and discussing* your results with your colleagues. During the last week of the quarter, we would like for you to take this opportunity. You and your partner should plan to make a short presentation to your classmates about your results. You may use Powerpoint, overheads and/or the blackboard as you like. This presentation should be about 10 minutes in length and ***explicitly*** present: 1) the hypothesis that you have tested; 2) the experimental results; and 3) your interpretation of the results.

Your presentation could describe the effect of your mutation on protein-protein binding affinities, the effect of modifications of phosphopanteheine derivatives on P450 binding, but could alternatively be focused on another aspect of our experiments (AUC analysis, enzyme binding and/or turnover experiments, mutagenesis, organic synthesis, protein purification, trouble-shooting, etcetera).

*Note that our goal is to engage one another in discussion, so please plan to contribute accordingly!*

**Grading of final research presentation (35 pts total)**

Background (5 pts)

- “Big picture”
- Background on the skyllamycin system
- Importance of proposed work
- Hypothesis and aims of research

Results (10 pts)

- Presented a complete record of what they accomplished
- Appropriate amount of data shown
- Results clearly presented
- Obstacles and troubleshooting

Conclusions and future directions (5 pts)

- Conclusions supported by data
- Thoughtful and feasible future experiments

Effective communication/delivery (5 pts)

- Flow of information on poster
- Illustrations/graphics on poster, effective use of text
- Pace
- Eye contact
- Division of labor

Engagement and answers (5 pts)

- Poised, succinct answers
- Thoughtful analysis/predictions

Symposium (5 pts)

- Presented at symposium on Thursday night
- Actively engaged with other posters on Thursday night

**Safety Notes**

There are hazards associated with working in a laboratory: **toxins**, **carcinogens** and, possibly, **allergens** are among the hazards you will encounter this quarter**.** You are probably familiar with most of these hazards, having encountered them in previous lab courses. To avoid injury, you must follow correct procedure when dealing with these hazards.

**TOXINS/CARCINOGENS:** acrylamide is a known toxin and polyacrylamide gels used for protein separation should be handled with gloves in the unlikely instance that some free acrylamide might be present. If we run any agarose gels for DNA analysis, ethidium bromide will likely be used and gloves must be warn when pipetting this chemical hazard. Other chemicals for which care must be taken include detergents (used in cell lysis and protein running buffer).

**ALLERGENS:** Among the possible allergens you may encounter are latex gloves, which you must wear to protect yourself from hazards and to protect your samples from enzymes on your skin. If you are aware that you are allergic to latex (or any other materials we will work with) or if you experience problems during the course, inform your instructor.

**SHARPS:** You will be using various sharp implements, such as syringes, in the lab. Please be sure to be trained in the proper use of syringes. Proper disposal of sharps will be covered as well.

**CENTRIFUGES:** Centrifuges may not be used without appropriate training. This training will be provided either by the faculty or department instrument specialists. Here are a few notes to remind you of safe practices in using centrifuges:

1. Always use appropriate bottles or tubes for centrifugation;
2. Do not overfill bottles or tubes;
3. You may use a balance or scale to make sure that pairs of tubes (bottles) are appropriately balanced in weight;
4. Always be sure that paired tubes (bottles) are placed in the rotor at positions opposite one another across the axis of rotation;
5. Be sure that rotor is placed in centrifuge properly, and that lid is properly secured;
6. Specific instructions for individual centrifuges will be posted as well;
7. Clean up any mess from accidental spillage; if you spilled bacterial cultures, ask for proper sanitizing clean up procedure.
8. **No eating, drinking or using mobile phones in the laboratory**
9. **Wear gloves and change your gloves if they become contaminated**
10. **Wash your hands when leaving the laboratory even though you have been wearing gloves.**
11. **Dispose of contaminated materials correctly**
12. **Be sure to monitor open flames, and to shut off gas valves when work is completed.**

I have read and understand these safety notes____________________________________

A signed and dated copy of this statement should be in your lab notebook.

**Introduction to protein model system and experimental approaches**

When faculty co-teach superlab, they often will want to take advantage of interests and expertise related to their own scholarly work. This superlab is no different. We take advantage of Lou’s interest and expertise in protein model systems involved in the fascinating and hot area of non-ribosomal protein synthesis. This is emerging as an area of research that could very well provide exciting new approaches to solving the world crisis in bacterial pathogen resistance to the current classes of antibiotics that have served the biomedical community so well in the past 70 years. This interest is married to Rob’s expertise in protein expression and purification, developed in work that he had done for seven years in the pharmaceutical industry, and continued here at Haverford. The biochemical and biophysical approaches that will be applied take advantage of the rich variety of instruments that support more broadly the science faculty scholarship that exists at Haverford.

We start with a basic introduction to non-ribosomal peptide synthesis (which is very well covered in Wikipedia, and served as an organizational source of material for this introduction; <http://en.wikipedia.org/wiki/Nonribosomal_peptide>). Non-ribosomal peptide synthetases (NRPSs) represent a very clever way for bacteria, fungi, and certain other organisms to circumvent the genetic restriction imposed by ribosomal synthesis, which limits chain incorporation to the 20 canonical (and three non-canonical) amino acids. There are limited modifications that can occur to amino acids post-synthesis, such as phosphorylation, lipidation, and glycosylation, to name a few. Bacteria and fungi have evolved protein complexes that act to bypass this synthetic pathway, leading to a huge variety of unnatural amino acid functionalities that may be incorporated into peptides, such as cyclization, oxidation, and adenylation, to name a few. Such peptides are typically involved as secondary metabolites, involving functions such as antibiotics (e.g., vancomycin) and immunosuppressants (e.g., cyclosporine), with these examples being of particular biomedical interest.

Non-ribosomal peptide synthesis machinery has significant similarity to the machinery that is involved in building other secondary metabolites (e.g., polyketides) and fatty acids. In general, such machinery consists of very large arrays of modular multi-enzyme functions, often residing in a single operon, resulting in these functional “domains” residing within a single polypeptide chain. The molecular backbones for NRP assembly versus polyketide and fatty acid assembly are controlled by the adenylation (NRP) and acyl transferase (polyketide and fatty acid synthases) proteins that collaborate with the carrier proteins to present the substrate to the assembly for enzymatic modification. Acyl transferase enzymes pick up simple malonyl CoA building blocks and pass this substrate to acyl carrier proteins to build up polyketide and fatty acid chains via decarboxylative Claisen-like condensations. Adenylation domains help load amino acids onto the peptide carrier proteins that are then incorporated into NRPS via peptide bond forming reactions. Perhaps it is not surprising that, based on the modular nature of the enzymatic functions within a complex, polyketide modules and NRPS modules can be found within the same multi-enzyme complex!

The different types of enzymatic functions that may be found in NRPSs can be reviewed in the Wikipedia site, but for our purposes, it is valuable to call out the following synthetic functions (involved in skyllamycin synthesis; more detail provided below): condensation, adenylation, thiolation, epimerization, beta-oxidation, and thioesterification. Various combinations of these enzymatic domains are responsible for addition/modification of each amino acid in an NRP. The specific combination of domains that are used to incorporate and modify an amino acid defines a module. So, for example, for production of skyllamycin, 11 modules are required to synthesize this 11-amino acid secondary metabolite. At a minimum, three enzymatic domains are required to add an amino acid, those that are responsible for amino acid activation (A=adenylation), covalent loading of the activated amino acid via the PCP (T=thiolation), and finally, condensation of this amino acid to the growing chain (C=condensation). Of course, other domains may be involved that might modify the amino acids or the chain (e.g., oxidation, cyclization).

We have chosen to study several enzymatic domains from a particular multi-enzyme complex involved in making the molecule, skyllamycin, a secondary metabolite from *Streptomyces* that can act to inhibit the platelet-derived growth factor signaling pathway, making it of biomedical interest. This complex was chosen because the reagents were readily available from Lou’s collaborator, Max Cryle (Max Planck Institute/Monash University), and because it was an excellent candidate to provide teaching opportunities in protein expression, purification, mutagenesis, and organic synthesis. Furthermore, this system is particularly attractive because Max Cryle has recently solved the structure of the complex of an oxygenase domain with its partner PCP, providing a fantastic tool for structure-based hypothesis building, a central purpose of this lab.

Skyllamycin is largely synthesized by a multi-enzyme NRPS produced from a single gene cluster, in which a series of genes, designated as “SkyN” genes, encode a set of polypeptide chains for the synthesis of this molecule. The particular enzymatic function, or domain, that we are interested in is an oxidation reaction, involving Sky32, a separate polypeptide encoding a heme-containing cytochrome P450 enzyme. The role of this enzyme is to beta-hydroxylate three amino acids in skyllamycin: phenylalanine, tyrosine, and leucine. These amino acids are presented to the cytochrome for modification by PCPs contained within modules 5 and 7 of Sky30 and module 11 of Sky31. See for details: Uhlmann et al., (2013) *ACS Chem Biol*, 8(11):2586.

In this lab, we will focus on the interaction of cytochrome P450_sky_ with its target substrates, as presented by the PCP_7_ and PCP_10_ domains. What we hope to learn is why P450_sky_ can recognize and modify amino acids bound to PCP7, since the enzyme normally will not modify the free amino acid nor amino acids bound to PCP_10_. We have obtained His-Tag fusion expression constructs for all three of these enzymatic domains, and your goal is to design a set of experiments that will provide us with greater insight into the binding affinity and specificity for P450_sky_ for various derivatives of the PCP_7_ and PCP_10_ constructs.

How do PCP_7_ and PCP_10_ (and CPs in general) present the “activated” amino acid for condensation reactions, or in our case, hydroxylation by P450_sky_? PCPs are modified to contain a phosphopantetheinyl (Ppant) arm as the covalent partner for the appropriate amino acid, through a thioester linkage

The Ppant arm itself is derived from Coenzyme A and added through a phosphate linkage to a conserved serine in the PCP. This flexible 18 Å arm presumably allows for the presentation of the amino acid to a variety of enzymatic active sites (for condensation or oxidation in this case), so the question of what defines the specificity of oxidation is left dangling. Perhaps the specificity is encoded in specific contacts between the enzymatic domain and the PCP? We are interested in exploring this question, and to do so, we would need to compare the ability of P450_sky_ to recognize and bind apo-PCP (no Ppant arm), holo-PCP (PCP with Ppant), and holo-PCP in which the Ppant has been loaded with the appropriate substrate. So this is where the organic chemistry component of the lab becomes important. In order to test various substrates (or inhibitors, should you wish) on P450_sky_ engagement, the substrates would have to be covalently attached to Coenzyme A. Having accomplished this, the substrate-Ppant complex would then have to be enzymatically added, using phosphopantetheinyl transferase, or Sfp) to the apo-PCP by phosphorylation of the appropriate target serine

This biochemistry superlab follows in the long-standing tradition of hypothesis-driven approaches to laboratory instruction, in which students are encouraged to take ownership over the direction of their project. Towards supporting this training goal, we encourage you to think about the background materials (including the relevant primary literature), along with the techniques and methods that we have made available to you, and develop your own set of questions that you wish to address. To help guide you in formulating specific plans, some general questions are provided here, and arise from a careful reading of the two Cryle papers that we will be studying:

1. Why does P450_sky_ oxidize amino acids attached to the isolated PCP_7_ but does not seem to oxidize amino acids attached to the isolated PCP_10_?
2. Do the PCPs that interact with P450_sky_ have a different structure than those that do not interact?
3. To what extent are protein-protein interactions between PCP_7_ and P450_sky_ important for affinity and specificity?
4. Does the phosphopantetheine arm of PCP_7_ interact with P450_sky_?
5. How does the solvent accessibility and/or dynamics of the phosphopantetheine arm change upon binding P450_sky_?
6. How does the presence of a substrate/inhibitor affect phosphopantetheine arm conformations and protein-protein interactions?
7. Is the nitrogen at the beta position of the substrate important for productive PCP_7_- P450_sky_ interaction?
8. Does the presence of an inhibitor facilitate PCP binding via conformational change to the P450_sky_?

You will have a variety of biochemical and biophysical methods available to you to explore these questions. Of course, reagent preparation is a key critical first step to be able to apply such methods. You will have available to you protein fusion constructs of P450_sky_, apo-PCP_7_, holo-PCP_7_, apo-PCP_10_, and holo-PCP_10_, which will therefore require that you express, isolate, and purify the appropriate proteins. The expression will be carried out using highly optimized plasmid/bacterial systems, followed by purification, principally focusing on affinity purification, taking advantage of a histidine tag amino acid sequence. You may also need to take advantage of various anion exchange or gel filtration purification methods. We will also be providing you with the appropriate tools to study the structure and dynamics of these proteins (circular dichroism spectropolarimetry, infrared spectroscopy, and analytical ultracentrifugation), the binding of the appropriate pairs of proteins (analytical ultracentrifugation, or the enzymatic turnover (uv-visible spectroscopy, HPLC, and LC-MS). You will also be given the reagents and tools to make changes in either the proteins themselves (site-directed mutagenesis) or in the particular substrate or inhibitor bound to the Ppant arm. We will be providing you with details about the protein constructs, as well as basic protocols for the various experimental procedures that you will likely need to carry out. The appendices in the manual (see below) will provide you with a basic understanding of the principles of the tools and experimental protocols. You are now “armed” with the information that you need to carry out the work in our brand new biochemistry lab, so go forth and have fun!

**APPENDIX 1: BACKGROUND MATERIALS**

**Bacterial Expression Growth**

The goal in this experiment is to grow bacteria transformed with a vector containing a gene encoding your fusion proteins. You will be picking a single colony and preparing a saturated culture as an inoculum for the culture in which you will be inducing expression. You will need about 500 mL of culture for your experiments in the expectation that 2-3 mgs of protein will be sufficient for your binding studies.

The construct you will use is a fusion of either the cytochrome P450 or the PCP to a “His-tag” amino acid sequence created by inserting the appropriate genes into a derivative of pET28a, a commercially available vector (Novagen) used for protein expression and purification. In addition, the PCP constructs also have the thioredoxin protein fused upstream of the His-Tag as a solubilizing agent. This places the gene encoding the protein just downstream of a T7 phage promoter, a *lac* operator site, a ribosome binding site (RBS), and a sequence that encodes a stretch of 6-histidine residues. The T7 promoter is recognized by the bacterial RNA polymerase in order to make the RNA transcript. The translational apparatus can synthesize a protein containing both the target protein sequence and the His-Tag sequence in a single polypeptide chain when induced by the lactose analog, isopropyl-beta-D-thiogalactopyranoside (IPTG).

The construct will be expressed in the Rosetta strain, which is a modified form of the bacterial strain, BL21(DE3), that has been constructed specifically for inducible expression of proteins cloned next to the T7 promoter. In this strain, researchers have inserted a piece of DNA into the bacterial chromosome (DE3) that contains the gene for the T7 RNA-polymerase behind a *lac* promoter and operator. This cell line also contains an F’ episome that encodes the gene (lacI) for the lac repressor protein. When we add ITPG into the medium, abundant expression of T7 polymerase is induced that will transcribe the His-tagged protein fusion gene from the pET vector. The vector is maintained in the bacteria because it contains a gene for neomycin phosphotransferase II, an enzyme that breaks down the kanamycin that we introduce into the LB medium, thus providing the host bacteria with kanamycin resistance. The Rosetta BL21(DE3) modifications of this bacterial expression system allow for greater expression of genes that use codons that are typically less frequently used by bacteria, and could potentially limit the overall expression of heterologous proteins. PCP constructs can also be expressed in BAP1(DE3) cells, which harbors a gene encoding the Sfp phosphopantetheinyl transferase enzyme, to afford the holo-PCP.

**Affinity Chromatography Purification**

There are three general classes of column chromatography used in the purification of proteins. These are ion exchange, gel filtration, and affinity chromatography. Ion exchange chromatography separates proteins by differences in their charge. Gel filtration separates proteins by molecular weight. Perhaps the most efficient way to purify proteins is to take advantage of their function; this is generally referred to as affinity chromatography. For example, enzymes that use ATP as an energy source can be purified by passing them over a column support in which a non-hydrolysable analog of ATP has been immobilized. Such a column would selectively bind your protein and allow all proteins that do not bind ATP to pass through.

Another effective way to increase the purity of a protein that has similar properties to other proteins is to elute your protein in a buffer gradient. For example, since our His-Tag fusion protein is eluted with imidazole from the nickel column, one can set up a gradient of imidazole concentration. Such a concentration gradient applied to your column will selectively elute proteins according to their affinity to the immobilized nickel. We will not be performing a gradient elution; rather we will elute our protein at a single concentration of imidazole. This will hopefully still achieve greater than 90% purity for our case, although for other proteins it may be necessary to run a gradient to achieve the necessary levels of purity. We expect the molecular weights of the Thioredoxin-His-Tag PCP fusion constructs to be ~ 22 kDa and the His-Tag P450_sky_ fusion construct to be ~ 45 kDa.

Nickel chromatography takes advantage of the fact that histidines, because of their imidazolium side-chain functionalities, will bind to the column by chelating the nickel. Many vectors have the advantage of carrying the His-Tag sequence, a stretch of either 6 or 10 histidine residues that can be expressed at the N-terminal or C-terminal end of the target protein (covalently attached as part of the polypeptide chain, creating a “fusion protein”). The His-Tag sequence binds to divalent cations (e.g. Ni^2+^) immobilized on a metal chelation resin. After unbound proteins are washed away, the target protein is recovered by elution with imidazole. This system provides a convenient, economical means of purification without the need to develop new protocols for each protein. It also allows the purification of target proteins under gentle, native conditions for maintaining activity of soluble proteins, as well as under denaturing conditions necessary for solubilization of inclusion bodies. Up to 20 mgs of target protein can be purified on a single 2.5 ml column. (Recommendation: P450 shouldn’t be more than 5 mg/mL concentration or it will aggregate; so for a 1 Liter culture (assume 20-30 mg yield), use a 3-4 mL Ni-NTA column to avoid over-concentrating the protein during the elution.

**Measurement of Protein Concentration Using the Pierce Coomassie Plus Reagent**

Several protein assay methods are routinely used by protein chemists to quantify total protein. Although these assays provide data about how much protein is in a specific sample, they all have shortcomings. A perfect protein assay reagent is yet to be developed, however, most researchers would agree on its characteristics:

- Fast
- Easy to perform
- Very sensitive
- Accurate
- Precise
- Free of interfering substances

We will be using a Coomassie dye-based assay to measure protein concentration (Pierce; their website is [www.pierce.com](http://www.pierce.com) ). This assay is based on the Bradford method, which uses Coomassie brilliant blue G-250 and was developed originally in 1976. This assay method is based on the immediate absorbance shift from 465 to 595 nm that occurs when this dye binds to proteins in an acidic solution. Upon addition of sample, the dye will bind protein, resulting in a color change from greenish brown to blue. This dye is assumed to bind to protein via an electrostatic attraction of the dye's sulfonic groups, principally to arginine, histidine, and lysine residues. It also binds weakly to the aromatic amino acids, tyrosine, tryptophan, and phenylalanine via van der Waals forces and hydrophobic interactions. Coomassie dye-based assays are known for their non-linear responses over a wide range of protein concentrations. Thus, it is important to generate a standard curve using a protein of known concentration. We will be generating a standard curve using a known concentration of the protein, BSA (bovine serum albumin).

You will be using this assay to quantify the protein concentrations of all the samples that you saved from both Week 2 and Week 3. You will be assaying a total of 11 samples:

1. Centrifuge supernatant

2. Cell pellet resuspension

3. “Depleted extract”

4. Bind eluent

5. Wash eluent

6. - 11. Elute fractions 1-6

You will be using the information from the Coomassie assay to decide which fractions from the elution you will combine. Once the appropriate fractions have been combined, it will be important to prepare the protein for THE BIOCHEMICAL STUDIES in subsequent weeks. You will need to concentrate the protein to about 0.6-1.0 mg/mL and exchange the elution buffer with a buffer that is appropriate for protein binding assays.

**Polyacrylamide Gel Electrophoresis (PAGE) for Evaluating Purification**

PAGE is the single most important procedure used in the characterization of the purity of proteins. It is sensitive (μg quantities), rapid, convenient and relatively inexpensive. PAGE can serve a number of purposes. It: 1) resolves protein mixtures especially effectively when used in the two-dimensional mode, 2) permits rough quantitation, 3) provides data to calculate apparent molecular weight, and when used in conjunction with a blotting technique it 4) permits identification of protein chains by immunological and sometimes enzymological methods.

We will use PAGE as an assay system to follow the effectiveness of our purifications and to confirm the expected molecular weight of the PCP and P450 fusion constructs. Protocols for using the vertical gel apparatus will be provided in lab.

**UV Absorbance to Measure Protein Concentration**

Solutions of organic compounds often absorb light at specific wavelengths. This property can be used to measure the concentrations of these compounds, without using up any of the material being measured. Absorption spectra are measured with an instrument called a spectrophotometer, which can produce a parallel beam of monochromatic light over a range of wavelengths. This beam passes through an absorption cell, after which the energy is measured by a photomultiplier.

Beer's Law

The amount of light absorbed by a solution is dependent upon the concentration of solute “absorbaphores” (or absorbing molecules) in that solution. Beer demonstrated that the intensity of light obtained when light passes through a solution of concentration, c, and length, d, is equal to that obtained when light passes through a solution of the same substance at concentration c/2 and length 2d. Generalized, Beer's law states that:

LIGHT ABSORPTION IS PROPORTIONAL TO THE NUMBER OF MOLECULES OF ABSORBING SUBSTANCE THROUGH WHICH THE LIGHT PASSES.

Thus, as you will be testing with your own protein preparations, provided that all other components of the solution are transparent to that wavelength of light, THE ABSORPTION OF THE SOLUTION IS PROPORTIONAL TO THE MOLAR CONCENTRATION OF THE PROTEIN.

Mathematically, Beer’s Law can be expressed as follows:

**A = εcl**

where: **A** = absorbance

**ε** = molar absorptivity or extinction coefficient in liters/mole/cm

**c** = concentration in moles/liter

**l** = path length of sample cell in cm

The molar extinction coefficient can be thought of as the standard absorbance, or optical density, of a 1 M solution of the substance as measured using a 1 cm sample cell.

Proteins absorb ultraviolet light at in the range of 190-300 nm. Proteins can most sensitively be detected in the range from 190-240, this wavelength range is often referred to as the far UV. The amide bond contributes to absorbance in this range and has a maximum absorption at 214 nm. Proteins will also absorb light in the near UV, corresponding to a range from 250-300 nm. This range includes absorption principally from the aromatic amino acids tryptophan, tyrosine, and phenylalanine and disulfide bonds.

Protein chemists will typically use the near UV (usually 280 nm) to quantify their protein concentrations rather than the far UV because many solvents will absorb or scatter light, and thus interfere, with the absorbance of the amide chromophore. Aromatic amino acids, such as tyrosine and tryptophan have large, and well characterized, extinction coefficients in the 250-300 nm range. Thus, if one knows the number of tyrosines and tryptophans in a protein, one can calculate an extinction coefficient for that protein for a given wavelength and use this to measure the protein concentration in solution.

ε_280 nm_ for tyrosine = 1280 cm^-1^ M^-1^

ε_280 nm_ for tryptophan = 5690 cm^-1^ M^-1^

as an example, for Thioredoxin-His Tag-PCP_7_ = (1x5690)+(3x1280) = 9,530, 1/ ε = 105 μM

For example, if we measure an absorbance at 280 nm for the His-Tag fusion of 1.0 using a 1 cm cell, we can use Beer’s Law to find that:

**A = ε cl**: 1.0 = 9,530 x 1.0 x **c** and solving for **c**: [protein fusion] = 1.05 x 10^-4^ M.

Of course, this molar extinction coefficient should only be used when dealing with highly purified proteins. A rule of thumb that can be used to monitor total protein concentration in a sample of a mixture of proteins is:

OD_280_ = 1.0 absorbance unit for a 1 mg/mL protein solution.

In addition to quantifying your protein concentration, an absorption spectrum will give information about certain properties of your protein solution that is often important in evaluating the quality of your protein. Such information includes identity of dominant aromatic groups and presence of aggregated protein. This technique is not without its artifacts. You might have problems in getting a high quality spectrum due to interfering absorbance by certain buffers and by not correcting properly with your blank sample cell; you should watch out for these problems. Presence of these artifacts can be ascertained by measuring the protein spectrum and allows for a more informed interpretation of the absorbance measured at 280 nm for protein quantification.

**PCR Mutagenesis and Bacterial Transformation**

Designing a mutant

Based on your hypothesis, you may wish to consider making mutant proteins. The final decision for which mutation you choose to make will be determined by the outcomes of the exercise outlined below and from talking with your instructors.

The particular protocol that we will be using for creating your mutation will involve designing two overlapping 25-45 base-long oligonucleotides (or primers) that contain your mutation. Of course, you will need the DNA sequence of the gene for the fusion construct to design your primers; this is provided on the following pages. The following considerations should be made for designing mutagenic primers.

1. Both the mutagenic primers must contain the desired mutation and anneal to the same sequence on opposite strands of the plasmid.
2. Primers should be between 25 and 45 bases in length, and the melting temperature (T*_m_*) of the primers should be greater than or equal to 78°C. The following formula is commonly used for estimating the T*_m_* of primers:

T*_m_* = 81.5+0.41(%GC)-675/N-%mismatch

For calculating T*_m_*:

1. N is the primer length in bases
2. Values for %GC and % mismatch are whole numbers
3. The desired mutation should be in the middle of the primer with ~10-15 bases of correct sequence on both sides.
4. The primers optimally should have a minimum GC content of 40% and should terminate in one or more C or G bases, but this is less important.

Turn in your sequences for your two primers. Follow the formatting directions given in class carefully, so that we can order them with the minimum of delay. We will order these primers and barring unexpected delays in the synthesis of your oligonucleotide primers, you can perform the mutagenesis in the following week. Stay posted, though.

Mutagenesis Protocol

This week, you will begin generating your mutant PCP or P450 gene and transforming the product into the bacterial expression system. This is a two-step procedure that you will carry out over two days. On the first day, you will set up a PCR reaction to generate the actual mutation in the plasmid DNA. On the second day, you will confirm the presence of the PCR product by gel electrophoresis, and then transform the DNA into bacteria. Because there have been past problems with the efficiency of transforming the mutagenized DNA directly into BL21-derived cells, we will be transforming first into a strain that acquires external DNA with high efficiency (DH5α or XL1-blue). The DH5α will give us the opportunity to expand and recover the mutant plasmid for transformation into the BL21 strain.

DNA mutagenesis techniques are commonly employed in biology to alter DNA sequence and ultimately the sequence of expressed proteins. Random mutagenesis is employed when we know what effect we would like to see (e.g. a specific phenotype) but not what DNA changes might cause that effect. In contrast, site-directed mutagenesis, the method we will use, is employed when we want to test a hypothesis about a specific DNA change. We decide exactly what to change, and then look to see if we obtain the hypothesized effect. A variety of techniques have been developed for site-directed mutagenesis. These methods commonly employ three steps:

1) Generation of DNA that contains the desired mutation. To accomplish this step, we will use PCR (polymerase chain reaction) with two overlapping DNA oligonucleotide primers that encode the DNA alteration and are oriented in opposite directions on the template DNA. As a result, we will end up amplifying the entire plasmid. This is why this PCR reaction is affectionately known as “around the world” PCR. In the process, we will incorporate the desired mutation.

2) Removal of the unmutated, template DNA. Following the PCR reaction, we are left with a mixture of both the original, unmutated template DNA and the mutated newly synthesized DNA. As we only want the newly synthesized DNA, we must have a way of identifying and eliminating the original template. DNA synthesized in *E. coli* is efficiently methylated at specific nucleotides, whereas DNA synthesized in a PCR machine remains unmethylated due to the absence of DNA methylating enzymes. We will use a restriction enzyme called *DpnI* that cleaves DNA into smaller pieces. Restriction enzymes bind to specific sequences in DNA prior to cleavage, and it happens that *DpnI* binds (and therefore cleaves) exclusively DNA that contains methylated adenines in the following sequence:

CH 3

I

5’…GATC…3’

5’…CTAG…3’

I

CH 3

Therefore, digestion by *DpnI* leaves the PCR generated DNA intact, but cuts the original template DNA into many pieces that will not be maintained in *E. coli* because only circularized DNAs are replicated and inherited.

3) Amplification of the mutagenized DNA. We then transform our DNA into *E. coli*, where bacterial enzymes (DNA ligases and topoisomerases) repair the PCR synthesized DNA and amplify the DNA plasmid. Because we have had trouble transforming directly into the bacterial strain used for expression, we will transform into a cell line specifically designed for optimal recovery of plasmid DNA and then re-transform into an expression cell line.

If the site-directed mutagenesis is successful, we will have a) introduced the desired change into the DNA sequence, and b) not introduced any independent, spurious changes that might have an effect on the protein activity or expression. We will use a high fidelity DNA polymerase with DNA editing functions (*Pfu*) in the PCR to reduce the probability of incorporating spurious changes into the DNA. DNA sequencing of the target gene in the vector that we recover can be used to determine unambiguously that the desired changes, and only the desired changes, are present in the gene. In the past, 95% of the colonies obtained following transformation have been correctly mutated.

**Preparation of Mutated Plasmid DNA**

One of the common procedures used in molecular biology is “minipreps.” Minipreps are used to purify plasmid DNA from small culture volumes (1.5-5 mL). This DNA can be used for further manipulation (e.g. through restriction digestion and insertion of a new piece of DNA into the plasmid using DNA ligation), for determining the DNA sequence of a portion of the plasmid, or for introduction into a new host by DNA transformation. We will use the miniprep procedure to recover the plasmids that harbor our mutations from the DH5α blue cells so that we can introduce these plasmids into BL21 cells, for mutant PCP/P450 expression and purification. We cannot use the DH5α strain for expression of mutant PCP/P450 from the pET vector because the expression of the gene in this system is driven by T7 polymerase, and the gene for this polymerase is not present in the DH5α strain (as it is in the BL21 (DE3)-derived Rosetta strain and BAP1).

We will use a kit sold by Qiagen ([www.qiagen.com](http://www.qiagen.com)) to prepare the plasmid DNA. The purification involves three steps:

1. Lysis of the bacteria in an alkaline solution with SDS, a detergent. This step destroys the outer membrane of the bacterial cells and permeabilizes the cell wall. Relatively small DNA molecules are released into the solution efficiently, whereas the larger chromosomal DNA molecules remain largely associated with the cellular debris.
2. Precipitation of the cellular debris, the SDS and most of the chromosomal DNA by addition of potassium acetate.
3. Purification of the plasmid DNA from the supernatant on a small column. The plasmid binds to the column by electrostatic interactions. The column is washed to remove contaminating biomolecules, and the plasmid DNA is then eluted from the column.

Following completion of this procedure, the next step is to transform Bl21-Rosetta cells with the DNA followed by plating the cells onto LB+kanamycin plates that are to be found in sleeves in the refrigerator.

**Biophysical methods**

Circular dichroism spectropolarimetry is a powerful and important tool in the study of protein structure. It is used to analyze secondary structure for proteins and its greatest utility is for proteins whose atomic structures are not known. However, even for proteins whose structures have been determined by x-ray crystallography or NMR spectroscopy, analysis of secondary structure can be a rapid method to determine the structural integrity of a purified protein. Circular dichroism has historically been an important tool in studies of protein folding and stability as it is a sensitive and reliable probe of the structure and stability of proteins. In the first experiment, you will be assaying the structural integrity of your purified fusion protein. In the second, optional experiment, you will determine the stability of your fusion protein using GuHCl, a chemical denaturant, to unfold the protein. The data that you collect can be analyzed to determine an equilibrium constant (or a thermodynamic free energy) to quantify the stability of your protein.

Analytical ultracentrifugation (AUC) is enjoying a renaissance in its use in the quantitative study of protein-protein interactions. It has been used to study self-associating mechanisms resulting in the formation of oligomeric proteins and has also been used to study associations between different proteins. Analytical ultracentrifugation was originally developed in the 1920's to determine covalent molecular weights of polypeptide chains and to determine protein. This ground-breaking work was done by Svedberg and we recognize him as the father of this technique. With the advent of SDS-PAGE, the use of analytical ultracentrifugation decreased significantly, being relegated to its use in only a few laboratories in the world. Today, since new instrumentation and simpler methods for data analysis have been developed, there are many new laboratories that are using this technique. Two principle methods using this instrument include sedimentation velocity and sedimentation equilibrium experiments. Sedimentation velocity experiments are particularly useful for studying the size and shape of very large macromolecules (>100,000 daltons) while sedimentation equilibrium measurements are best suited for quantifying protein-protein interactions and determining absolute molecular weights of proteins in the size range of 1,000-100,000 daltons. Sedimentation equilibrium experiments are unique in determining molecular weights of proteins because the method employs direct physical equations applying concepts in diffusion and flux. Most other methods to determine molecular weights are based on empirical approaches using comparative methods (ie, using molecular weight standards in SDS-PAGE for molecular weight determination).

Another physical technique that can be used to understand conformational flexibility of proteins is vibrational spectroscopy. Infrared absorption spectroscopy (IR) and Raman scattering spectroscopy can be used to determine the conformational distribution of protein structures in solution. Traditionally, vibrational bands of native and modified amino acids are studied to determine structural distribution and solvent exposure at the site of the amino acid. The Charkoudian and Londergan labs are currently collaborating to use site-specific vibrational spectroscopy to study acyl carrier protein structural dynamics involved in polyketide biosynthesis. The terminal thiol of the Ppant arm of holo-carrier protein can be chemically converted into a thiocyanate (SCN) moiety. The thiocyanate is a powerful vibrational spectroscopic probe because it displays a strong vibrational stretch in the region of the IR that is void of other protein bands. The frequency and bandwidth of the thiocyanate peak reports on the local solvation environment of the Ppant arm, and thus one can distinguish between the Ppant arm being solvent exposed versus tucked inside a hydrophobic cavity of the protein with picosecond resolution. The conformational dynamics of PCPs have yet to be studied using this technique, but given the success of using the thiocyanate probe to study acyl carrier proteins, it would be exciting to use this experiment to explore the conformational dynamics of PCPs. Moreover, it is possible that interactions between the thiocyanate-modified Ppant arm and enzyme partners could be visualized by other biophysical techniques, such as AUC and UV-vis.

Liquid chromatography-mass spectrometry (LCMS) is an analytical technique that combines separation chromatography with mass analysis. In this experiment, a sample is fractionated through a column (the “stationary phase”; usually a non-polar silica column) by a carrier solvent at high pressure (the “mobile phase”). Molecules will separate on the column based on polarity and therefore reach the accompanying mass spectrometer at different times (“retention times”). The molecule will then be ionized (chemically or by electron beam impact) and the ions separated according to mass-to-charge ratio by electromagnetic fields. The ions are then detected to afford a mass spectra. Taken together, the retention time and mass spectra can facilitate the structural characterization of molecules and is often used to determine whether or not a reaction proceeded as expected. In both the Pohle 2011 and Uhlmann 2013 papers, LCMS was used to determine if a betahydroxylation reaction occurred.

**PROTOCOLS**

**Instructions For Using PyMol**

Use PyMol (which has been installed on each of the computers in the computer cluster) to explore possible sites in your proteins that you may wish to mutate to test specificity of protein-protein interactions. You will be using protein structures from Haslinger et al (2014). If you are not familiar with PyMol, a tutorial is included here to provide you with a basic working knowledge of the program:

Pymol is a program that displays information about protein structures obtained from techniques such as x-ray crystallography and NMR. It allows you to view the structure of a protein from multiple perspectives, highlight certain portions and make mutations. We will use it to look at structures from the Protein Data Bank.

Part A: accessing info from the Protein Data Bank (PDB)

1. go to http://www.rcsb.org/pdb/explore/explore.do?structureId=4PWV
2. this will take you to the page containing the co-crystal structure of P450sky in complex with a peptidyl carrier protein domain, as described in the Haslinger et al paper.
   1. The main page for this structure (the structure summary page if you look at the tabs on the top of the page) gives you information about how the structure was generated (x-ray diffraction) and the citation for the paper in which the structure was originally reported. It also tells you the general functional class of the protein and its different domains and their secondary structure.
   2. Click on the Biology and Chemistry tab. This gives you information about the protein, its molecular weight, other molecules that might be associated with the protein and what organism it was derived from.
   3. Click on the Sequence Details tab. This gives you the sequence of the chain of the protein using the 1 letter amino acid code. It also tells you the different secondary structures present in the protein.
   4. If you ever want to get back to the main page, just click the square next to the PDB ID (“4PWV”) on the left hand side of the page.
3. Download the PDB structure for use in Pymol.
   1. See the options under “download files” by clicking the triangle next to it.
   2. Click on “PDB file (Text)” and save it somewhere on the computer.
   3. Next open Pymol
      1. Go to File 🡪 Open and find where you saved the structure and open it (it will automatically save as 4PWV if you didn’t change the name)
      2. Click on the green button in the upper left hand corner to maximize the graphics screen.

Part B: using Pymol

1. how to control the movement of the protein structures
   1. left click and hold to rotate around a point
      1. to change the atom around which the structure pivots: press control + shift and then click the middle mouse button on the atom of interest
         1. this can be helpful for looking at different parts of the molecule
   2. right click and hold to move it away from you or towards you
   3. The scroll wheel lets you decided how much of the molecule is visible (how deep the field of view is)
   4. Pressing and holding the middle button lets you move the structure around within the view window
2. Showing the sequence associated with the structure
   1. It is often useful to look at the amino acid sequence to help guide your understanding of the sequence/structure relationship. To display the sequence, go to the Display menu and select “Sequence On”
   2. You can change the format of the sequence display by choosing “Sequence Mode” and selecting the appropriate format.
   3. The scroll bar below the sequence will allow you to view the entire sequence and all associated small molecules (i.e., water molecules are listed as “O”). If the protein contained more than one chain, each chain’s sequence would have a different color.
   4. If you left-click on any residue in the sequence, it will automatically highlight that residue in the structure and create a new selection in the selection list in the right hand window. As long as you keep left clicking, residues will continue to be added into this selection. Clicking anywhere else on the screen will complete the residue list that will be part of this selection. Left-clicking and dragging the mouse across the sequence will allow you to select a range of residues.
   5. If you click on any residue in the structure, the appropriate residue will be highlighted in the sequence. If you don’t see the residue highlighted, most likely it is because you chose a residue outside the window of displayed residues. However, the scroll bar can be moved to allow you to find the highlighted residue in the sequence.
   6. You can rename your selection to something more appropriate simply by left-clicking on the “A” button associated with your old selection name and choosing the “rename selection” option.
   7. You can change your selection criteria by clicking on the green “Selecting” tool in the right hand window. As you click on this text, it will change to different selection modes. The default is to select residues (single amino acids), but it is often useful to be able to select individual atoms or entire chains.
3. Look at the protein in different views
   1. Go to “S”🡪 show 🡪 as
      1. Pick cartoon to get sheets and helices
      2. Pick ribbon to just see the backbone
      3. Pick sticks to see all the side chains and backbones (how it when you first opened it in PyMOL
   2. Show it as a cartoon
4. How to select a subset of the structure for manipulating the view
   1. Use the sequence to select the PCP_7_ chain.
      1. You will name this selection something so that you can later go back to it easily. Lets name it “helix bundle”.
      2. Select the strand by choosing the appropriate residues in the sequence bar, then rename the selection “helix bundle”
         1. color this selection blue
         2. if you wish, you can now use the command line to execute commands on sheet1 rather than the mouse
            1. for example you can type “hide everything, helix bundle” to make it go away or “show cartoon, helix bundle” to bring it back or “color red, helix bundle”
   2. See the accompanying quick reference for pymol commands to select other types of things such as specific secondary structures
5. Saving your work
   1. To save your session where you left off go to file 🡪 save session as🡪 save it somewhere
   2. This saves your current view and all of your selections

Part C: Some exercises to do with the P450_sky_/PCP_7_ co-crystal structure in preparation for possible mutagenesis experiments

- Identify the two protein chains
  - Describe the secondary structures and structural motifs in the two chains
- Highlight the heme and represent as spheres
- Highlight the imidazole-modified pantotheine arm and represent as sticks
  - Describe the interactions between the two prosthetic groups. Why did the authors design this interaction? Is this interaction relevant biologically? Why or why not?
  - How far apart is the heme from the thioester of the pantetheine arm? What does this mean in terms of the size of cargo that can be accommodated on the PCP7? Could small changes in the P450_sky_/PCP interaction change this distance?
- Highlight the residues from the Haslinger paper that show the interactions between the two chains.
- Consider the residues from PCP_10_ in place of the relevant residues in PCP_7_ interacting with P450_sky_. Do you expect that these changes can be accommodated? Based on your analysis, how might you predict the affinity to P450_sky_ would be affected? You may wish to use the mutagenesis wizard to change the sidechains in PCP_7_ to those of PCP_10_ to enhance your intuition:

1. Select Mutagenesis from Wizard pull-down menu;
2. Pick a residue to mutate by left-clicking on the residue of interest;
3. Under the Mutagenesis menu (lower right hand corner), select the “Mutate to” bar and choose the appropriate amino acid from PCP_10_;
4. “Apply” the mutation;
5. Click “Done” when you’re done making changes; you may wish to rotate the sidechain to see if there are better rotamers for maintaining the interaction of interest.

**Quick reference for Pymol commands**:

For more info see online manual: http://www.pymolwiki.org/index.php/Main_Page

Avoid these characters in the command line: ! @ # $ % ^ &* ( ) ' " [ ] { } \ | ~ ` <> . ? /

To include multiple things use “or”

To limit a selection use “and”

Use resn when selecting with the 3 letter amino acid code or 1 letter nucleic acid code

Use resi when selecting amino acids based on residue number

To select a chain: **select chain A**

To select all atoms in chains A, B and C: **select chain A or chain B or chain C**

To select all of a certain residue: **select resn ala**

To do this on a certain chain: **select resn ala and chain A**

To select a residue on all chains: **select resn ala and resi 75**

To select hydrophobic residues: **select hydrophobes,(resn ala+gly+val+ile+leu+phe+ met)**

On a certain portion of a chain: **select hydrophobes,(resn ala+gly+val+ile+leu+phe+ met) and resi 339-357 and chain A**

To select hydrophilic residues: **select hydrophilics,(resn arg+lys+his+glu+asp+asn+ gln+thr+ser+cys)**

To select basic residues: **select pos,(resn arg+lys+his)**

To select acidic residues: **select neg,(resn asp+glu)**

To select aromatic residues: **select aromatics,(resn phe+tyr+trp+his)**

To select heteroatoms: **select het**

To select a residue on just chain A: **select resn ala and resi 75 and chain A**

To select a chain and name it “bob”: **select bob, chain A**

To select all backbone atoms and name it bb: **select bb, name c+o+n+ca**

To make a selection of residues and name it “bob” for later use: **select bob, resi 1-10**

To do this just on one chain: **select bob, resi 1-10 and chain A**

To hide a selection: **hide everything, bob**

To show a selection again: **show cartoon, bob**

To get rid of surface or sphere view: **hide spheres**

To color the selection: **color blue, bob**

To color the whole pdb structure: **color orange, 1LBI**

To color specific residues: **color green, resi 50+54+58**

To color a number of residues in a row: **color yellow, resi 60-90**

To do this on a single chain type: **color yellow, resi 60-90 and chain A**

To color all helices: **color blue, ss h**

To color all beta sheets: **color purple, ss s**

To color all loops and unassigned residues: **color yellow, ss l+””**

To color the backbone: **color yellow, name c+o+n+ca**

To store a view (and name it v1): **view v1, store**

The view only saves for the duration of the pymol session

To recall this view: **view v1, recall**

**Bacterial Growth Procedure for P450_Sky_ and PCP7/10**

Day 1 (or better yet, afternoon/evening before Day 1)– Preparation of inoculum

1. Add 2x5 mL of TB containing 0.05 mg/mL kanamycin supplemented with 0.05% (w/v) glucose and 0.5% (v/v) glycerol. USE STERILE TECHNIQUE!

2. We will be providing you with an agar plate containing individual colonies for the expression growth. Carefully pick a single colony using a Pipetman and a sterile tip. Transfer one colony to each of your two tubes. You should only need one tube; we typically do an extra growth just in case one doesn’t grow.

3. Place your tubes into a shaking incubator set for 37°C. You will let your bacteria grow overnight.

Day 2 – Expression growth

You will be setting up large-scale growths of each transformed bacterial strain. You will be provided with a 2-Liter flask containing 500 mL of TB and with kanamycin antibiotic stock (50 mg/L).

1. Remove 2 mL of TB from your flask using a pipet and place 1 mL each into two plastic cuvettes. You will need these cuvettes later as a reference for the measurement of the optical density of your culture.

2. Add 5 mL of your inoculum to your flask and place your flask in a 37°C shaker/incubator.

4. Your culture will need to grow to an optical density, as measured at 600 nm, of 0.6-0.8; this should take about 2-3 hours. To check the density, remove 1 mL of culture and place it into a cuvette. Measure the optical density in the cuvette measurement option on the nanodrop spectrophotometer in E106. Discard the cuvette properly! We will have a beaker with some diluted bleach in it to be sure to kill the waste bacteria.

5. Once the density is at the right level, remove 1 mL of the culture and spin it down in an microfuge tube for 3 minutes. Remove the supernatant and add 100 µL of SDS-sample buffer to the microfuge tube. Label the microfuge tube as "pre-induction control". Vortex to resuspend the pellet and place in your rack in the lab freezer. This sample will be analyzed on your SDS-PAGE gel as a control for expression of the protein.

6. Immediately after removing the 1 mL culture for analysis, add 1.0 mM IPTG (you will be provided with a 1 M stock solution) to the growing culture.

7. Transfer the flask to an incubator that will be set to 18°C. This lower temperature helps to keep the newly expressed protein soluble. IMPORTANT NOTE: For expression of P450_sky_, you must add 0.5 mM δ-aminolevulinic acid. Allow the cells to grow overnight.

8. The next day, take another 1 mL sample and treat it as described in Step 5. This time, label the tube "post-induction control".

9. Pour the culture into a 500 mL centrifuge bottle.

10. Spin the samples in the refrigerated centrifuges (set at 4°C) at 10,000 rpm for 20 minutes. Remove the supernatant and let the bottles drain for about a minute. Label your bottles, and store them in a -80°C freezer for next week’s purification protocol.

**His-Tag Purification Procedure**

**Day 1 – Preparation of cell lysates.**

1. Take your cell pellet out of the freezer and place it immediately on ice.
2. Add 10 mL of cell extraction buffer directly to your centrifuge bottle. Carefully pipet the cells up and down until the cells are suspended homogeneously. ***Remember to keep the bottle on ice while resuspending the pellet!*** Incubate the suspension for 20 minutes on ice.
3. Transfer the homogenized pellet to a 50 mL falcon tube. Pack a beaker **very tightly** with ice and add water to create an ice slurry. You can use your fist to really pack in the ice. Place your tube (you should have to be forceful if you packed your ice properly) into the beaker for sonication.
4. Prepare the sonicator microprobe by rinsing it with MeOH and drying it with a Kim-Wipe. Sonicate your sample for 30 seconds using a power setting no higher than 7 and a duty cycle of 50%. Wait three minutes and repeat the 30-second sonication. Wait another three minutes and perform a third 30-second sonication. BE SURE THAT THE MICROPROBE DOES NOT TOUCH THE SIDES OR THE BOTTOM OF YOUR 50 ML TUBE.
5. Transfer your sonicated sample to a 30 mL centrifuge tube. (Save your 50 mL tube.) Spin your sample at 10,000 rpm for 20 minutes in a refrigerated centrifuge.
6. After the centrifuge has stopped, carefully remove the tube and decant your supernatant into your 50 mL tube. REMEMBER that you are saving the supernatant. The pellet contains cell debris, including some DNA, the membrane material, and insoluble protein.
7. Remove 100 μL of your supernatant and place it into an microfuge tube. Label this tube as spin super. Freeze this with your other samples for future analysis. (DO NOT add SDS sample buffer to this aliquot; it will be used to determine protein *concentration*!) Your sample is now ready for affinity chromatography. Should you choose to proceed directly, keep your sample on ice while preparing your resin (step 2 below) ***or*** store extract at -80°C for long-term storage.
8. Resuspend your pellet in an equal volume of your cell extraction buffer (10 mL). Save 100 μL of this in an microfuge tube and label this as spin pellet, for future analysis. You may discard the remainder of the resuspended pellet. You will be analyzing the protein content of the pellet by SDS-PAGE to find out how much of your protein was lost in the sonication procedure.

**Day 2 – Protein purification.**

1. Thaw the cell lysate in a water bath (room temperature).
2. Meanwhile, equilibrate the Ni-NTA agarose resin in cell extraction buffer (*minus* lysozyme). Transfer 2 mls of resuspended slurry to a 50 mL tube. Set the tube on the bench and allow the agarose to settle. Carefully remove as much of the supernatant as possible without removing the resin. Add 25 mL extraction buffer, and mix on the Nutator for 5 min. to equilibrate the beads.
3. Centrifuge for 1 min. at 1000 rpm to collect the beads. Remove and discard the supernatant.
4. Binding of the tagged protein to the beads. Add the cell lysate to the agarose beads. Place on the Nutator and incubate for 20 min. Save the second 50 mL tube.
5. Meanwhile, set up the column. Make sure the frit is sitting flat in the bottom. Attach the column to a ring stand using the clips provided.
6. Set the tube with cell lysate and agarose beads upright on the bench, and incubate for another 10 min., allowing the beads to settle to the bottom.
7. Remove the lysate carefully from the beads. You can return it to the empty 50 mL tube you saved. This is your “depleted lysate” fraction. Store it on ice.
8. Using a P-1000 with a cut-off tip or plastic pipette, resuspend the beads in ca. 2 mL fresh cell extraction buffer and transfer them to the column. Allow the beads to settle, **but do not run them dry** (you can add more cell extraction buffer on top).
9. Wash the column with 5 volumes of Wash Buffer A and 5 volumes of Wash Buffer B. Collect the eluent from each wash in separate 15 mL tubes, label them as your Wash Fractions A and B, and store them on ice.
10. Elute the bound protein with 6 volumes of Elution Buffer. Collect 1 mL fractions into microfuge tubes and label your tubes appropriately.
11. Store all of your samples at 4°C (preferably in the cold room).

**Recipes**

**1X Cell Extraction (Lysis) buffer (1 liter):**

50 mM Tris-HCl, pH 7.8

300 mM NaCl

10 mM imidazole

5 mM reduced glutathione

5% (v/v) glycerol

2 mM phenyl methyl sulfonyl fluoride (PMSF)

**1X Wash Buffer A (1 liter):**

50 mM Tris-HCl, pH 7.8

300 mM NaCl

**1X Wash Buffer B (1 liter):**

50 mM Tris-HCl, pH 7.8

300 mM NaCl

30 mM imidazole

**1X Elution buffer (1 liter):**

50 mM Tris-HCl, pH 7.8

300 mM NaCl

250 mM imidazole

**Bacterial Growth and Protein Purification Procedure for Putidaredoxin (Pd) and Putidaredoxin Reductase (PdR)**

Starting culture was grown in 30 ml-LB-ampicillin (100 μg/mL) (in 150 ml Erlenmeyer flask) to OD 600=0.4-0.8 and used to inoculate five flasks each containing 1 L 2xYT ampicillin (100 μg/mL) (2 ml of starting culture per flask). The flasks were shaken at 200 RPM at 37°C for 40 hours. IPTG is not necessary (lac promoter is leaky). The cells were collected by centrifugation.

Pd purification (NB: 20 mM BME should be present in all the buffers; (Pd is supposed to be unstable without it).

1) Lyse the cells with sonication in 50 mM Tris/HCl pH 7.4, clarify the lysate by centrifugation.

2) Load onto DEAE Sepharose FF column (bed volume –about 100 ml), brown band should form on the top of the column. Wash with starting buffer, elute in NaCl gradient (try 600 mM NaCl in buffer B, 100 ml column, about 300 ml of each buffer). Collect colored fractions.

3) Precipitate with ammonium sulfate (0.4 g/ml) (salt cut), dissolve in small volume of DI water, dialyze against 50 mM Tris/HCl, pH 7.4, concentrate, if necessary.

4) Run second DEAE column (HPLC).

Column – Protein-Pak DEAE (Waters) ~1.2x6.5 cm

Buffer A: 50 mM Tris/HCl, pH 7.4; 20 mM BME

Buffer B: 50 mM Tris/HCl, pH 7.4; 1 M KCl; 20 mM BME

Flow rate -1.2 ml/min

Run linear gradient from 0 to 50% B in 40 min. Monitor absorbance at 330 nm as well as 280 nm. Collect the fractions with absorbance at 330 nm.

5) Concentrate and purify by size-exclusion chromatography. I used Superdex 200, got satisfactory separation at up to 1 ml loading volume.

PdR:

Starting culture was grown in 30 ml LB amp 100 (in 150 ml Erlenmeyer flask) to OD 600=0.4-0.8 and used to inoculate five Fernbach flasks each containing 1 L 2xYT amp 100 (2 ml of starting culture per flask). The flasks were shaken at 220 RPM at 37 C overnight (~17 hours). The cells were collected by centrifugation. NB: All the buffers contained 7.5% glycerol

1) The cells were lysed with sonication in 20 mM Tris pH 7.4; the lysate was clarified by centrifugation
2) DEAE Fast Flow column (~50 ml), equilibrated in the same buffer, wash; elution with same buffer + 0.1 M KCl
3) Salt cut - 0.35 g/ml, buffer exchange.

4) Run second DEAE column (HPLC). Column – Protein-Pak DEAE (Waters) ~1.2x6.5 cm

Buffer A: 25 mM Tris, 7.5 % glycerol, pH 7.4

Buffer B: 100 mM Tris, 0.6 M KCl, 7.5 % glycerol, pH 7.4

Flow rate -1.2 ml/mil

Run linear gradient from 0 to 50% B in 40 min.

5) Some fractions were OK after that, the rest was concentrated and run on SEC (Superdex 200).

DNA sequences

>putidaredoxin gene (Pd)

ATGTCTAAAGTAGTGTATGTGTCACATGATGGAACGCGTCGCGAACTGGATGTGGCGGATGGCGTCAGCCTGATGCAGGCTGCAGTCTCCAATGGTATCTACGATATTGTCGGTGATTGTGGCGGCAGCGCCAGCTGTGCCACCTGCCATGTCTATGTGAACGAAGCGTTCACGGACAAGGTGCCCGCCGCCAACGAGCGGGAAATCGGCATGCTGGAGTGCGTCACGGCCGAACTGAAGCCGAACAGCAGGCTCTGCTGCCAGATCATCATGACGCCCGAGCTGGATGGCATCGTGGTCGATGTTCCCGATAGGCAATGG

>putidaredoxin insert

gtcgaccgaccaacagaaggttaccgccgtcctctgcgaggacggcacaaggctgccagcggatctggtaatcgccgggattggcctgataccaaactgcgagttggccagtgcggccggcctgcaggttgataacggcatcgtgatcaacgaacacatgcagacctgtgatcccttgatcatggccgtcggcgactgtgcccgatttcacagtcagctctatgaccgctgggtgcgtatcgaatcggtgcccaatgccttggagcaggcacgaaagatcgccgccatcctctgtggcaaggtgccacgcgatgaggcggcgccctggttctggtccgatcagtatgagatcggattgaagatggtcggactgtccgaagggtacgaccggatcattgtccgcggctctttggcgcaacccgacttcagcgttttctacctgcagggagaccgggtattggcggtcgatacagtgaaccgtccagtggagttcaaccagtcaaaacaaataatcacggatcgtttgccggttgaaccaaacctactcggtgacgaaagcgtgccgttaaaggaaatcatcgccgccgccaaagctgaactcagtagtgcctgaaatctataccccaccataaatcacctttttgccccatagcgtgtgaggataaacagATGTCTAAAGTAGTGTATGTGTCACATGATGGAACGCGTCGCGAACTGGATGTGGCGGATGGCGTCAGCCTGATGCAGGCTGCAGTCTCCAATGGTATCTACGATATTGTCGGTGATTGTGGCGGCAGCGCCAGCTGTGCCACCTGCCATGTCTATGTGAACGAAGCGTTCACGGACAAGGTGCCCGCCGCCAACGAGCGGGAAATCGGCATGCTGGAGTGCGTCACGGCCGAACTGAAGCCGAACAGCAGGCTCTGCTGCCAGATCATCATGACGCCCGAGCTGGATGGCATCGTGGTCGATGTTCCCGATAGGCAATGGtaaaccacaatggtaaaccactgcgagccaaaacagccgagcaggagcgcagtccggcaacaccttattaagcacatgccgaaccctatttgcagcgcttcatgcctgcaaagtcccgattgatgaaatccgggctccaagcaaggagcccggaatctctcaccgccacgaaatcaatggccaatcccggg

>putidaredoxinReductase gene

ATGAACGCAAACGACAACGTGGTCATCGTCGGTACCGGACTGGCTGGCGTTGAGGTCGCCTTCGGCCTGCGCGCCAGCGGCTGGGAAGGCAATATCCGGTTGGTGGGGGATGCGACGGTAATTCCCCATCACCTACCACCGCTATCCAAAGCTTACTTGGCCGGCAAAGCCACAGCGGAAAGCCTGTACCTGAGAACCCCAGATGCCTATGCAGCGCAGAACATCCAACTACTCGGAGGCACACAGGTAACGGCTATCAACCGCGACCGACAGCAAGTAATCCTATCGGATGGCCGGGCACTGGATTACGACCGGCTGGTATTGGCTACCGGAGGGCGTCCAAGACCCCTACCGGTGGCCAGTGGCGCAGTTGGAAAGGCGAACAACTTTCGATACCTGCGCACACTCGAGGACGCCGAGTGCATTCGCCGGCAGCTGATTGCGGATAACCGTCTGGTGGTGATTGGTGGCGGCTACATTGGCCTTGAAGTGGCTGCCACCGCCATCAAGGCGAACATGCACGTCACCCTGCTTGATACGGCAGCCCGGGTTCTGGAGCGGGTTACCGCCCCGCCGGTATCGGCCTTTTACGAGCACCTACACCGCGAAGCCGGCGTTGACATACGAACCGGCACGCAGGTGTGCGGGTTCGAGATGTCGACCGACCAACAGAAGGTTACCGCCGTCCTCTGCGAGGACGGCACAAGGCTGCCAGCGGATCTGGTAATCGCCGGGATTGGCCTGATACCAAACTGCGAGTTGGCCAGTGCGGCCGGCCTGCAGGTTGATAACGGCATCGTGATCAACGAACACATGCAGACCTGTGATCCCTTGATCATGGCCGTCGGCGACTGTGCCCGATTTCACAGTCAGCTCTATGACCGCTGGGTGCGTATCGAATCGGTGCCCAATGCCTTGGAGCAGGCACGAAAGATCGCCGCCATCCTCTGTGGCAAGGTGCCACGCGATGAGGCGGCGCCCTGGTTCTGGTCCGATCAGTATGAGATCGGATTGAAGATGGTCGGACTGTCCGAAGGGTACGACCGGATCATTGTCCGCGGCTCTTTGGCGCAACCCGACTTCAGCGTTTTCTACCTGCAGGGAGACCGGGTATTGGCGGTCGATACAGTGAACCGTCCAGTGGAGTTCAACCAGTCAAAACAAATAATCACGGATCGTTTGCCGGTTGAACCAAACCTACTCGGTGACGAAAGCGTGCCGTTAAAGGAAATCATCGCCGCCGCCAAAGCTGAACTCAGTAGTGCCTGA

**Bradford Protein Determination, Dialysis, and Concentration Procedures**

**1.** Make a master mix of Coomassie Plus working reagent, by mixing one part of reagent with one part of MilliQ-filtered water. You will need 1 mL mix for each of your unknown and standard samples.

**2.** Preparation of Standard samples. When using color developing reagents for measuring an unknown protein concentration, one must compare the color developed by the unknown with that of a known protein "standard." For this standard measurement we will use a 2 mg/mL solution containing Bovine Serum Albumin (BSA). It is important to obtain a "standard" value that will be within the sensitivity range of the assay used and within the linear portion of the plot correlating absorbance (A595 nm) to the concentration. Using your 2 mg/mL stock solution, choose a set of volumes in the range 0.5-5 μL. Probably 5-15 samples of differing volumes should be sufficient to generate a reliable standard curve.

**3.** Preparation of Unknown samples. Add 10 μL of each of your 11 unknowns to your reagent tubes. If the color development of these samples is too low to see a visual color change, go back and add 50 μL to a new dilution of the Coomassie Plus reagent.

**4.** Measurement of absorbance. Read the absorbance at 595 nm using the nanodrop UV/Vis in E106 as follows:

Adjust to zero absorbance using the blank (water and Coomassie reagent alone) and follow with measurements of absorbance with the rest of your samples.

Draw a standard curve by plotting the absorbance at 595 nm vs. μg of protein in your BSA standard samples. Using this standard curve, determine the amount of protein for each unknown protein sample. Note that you can conduct this analysis using the Nanodrop software. Here is a sample calculation:

Suppose your unknown sample read an absorbance of 0.2, which corresponds to 10 μg on your BSA standard curve. This means that you must have added this quantity of your unknown to your "working" sample (the sample whose absorbance you actually measured).

Make sure that you understand why the volume of the working sample doesn't matter; the only thing that matters is the volume of unknown that was added to your working sample. This is because you are working with mass units, not concentration units.

Therefore, since you added 10 μl volume to your measurement, for which you determined contains 10 μg protein, the concentration of the unknown must be 1.0 μg/μl, or 1.0 mg/ml. This is the concentration of your original sample!

**5.** Preparation of samples for SDS-PAGE. Based on your protein concentration determinations, withdraw the volume that would correspond to 5 μgs of protein from each of your eleven fractions and place into an appropriately labeled microfuge tube. Don't withdraw more than 15 μLs for any sample since volumes greater than this will not fit into your wells in your SDS-PAGE experiment. Then, add 5 μl SDS-sample buffer and freeze these samples. You will use these aliquots to analyze the purification procedure by SDS-PAGE next lab.

**6.** Dialysis and concentration of protein.

**Step A. Dialysis:** Based on your protein concentration determinations for your elution fractions, pool the appropriate fractions into a single tube. **If the pooled concentration is less than 0.6 mg/mL, you will need to follow step B below to concentrate your protein first.** In this step, you will need to replace the imidazole buffer with a buffer that contains 2 liters of:

20 mM sodium phosphate, pH 7.5

150 mM NaCl.

1 mM EDTA

1 mM DTT

You will do this by dialysis using a Slide-A-Lyzer (Pierce). This apparatus uses two membranes with molecular weight cutoffs of 3,500 Daltons to create a chamber, in a slide format, in which your protein will be retained but the buffer will be exchanged. Follow this procedure:

Add about 4 liters of buffer and a stir bar into a beaker and place in the cold room on a stir plate.

1. Using a 1 mL or a 3 mL syringe with an 18 gauge needle, draw up your sample, leaving a small amount of air in the syringe.

2. With the bevel sideways, insert the tip of the needle through one of the syringe ports located at a top corner of the Cassette.

3. Inject sample slowly. Withdraw air by pulling up on the syringe piston.

4. Remove the syringe needle from the Cassette while retaining air in the syringe. BE SURE TO SAVE YOUR SYRINGE TO REMOVE YOUR SAMPLE TOMORROW!

5. Attach a buoy to your Cassette and place into your 4 liter beaker for overnight dialysis. We should be able to fit about 6-8 samples per beaker.

6. After your dialysis is complete, remove your sample, transfer it to a 1.5 mL microfuge tube, along with a 1.5 mL aliquot of the dialysis buffer as well, label your tubes and store them in the cold room.

Step B. Concentration**:** You may need to concentrate your protein to 0.6-1.0 mg/mL. Use your Coomassie assay to estimate what volume your solution will need to be to give you this concentration. We will use Centricon microconcentrators for this step. The microconcentrators are centrifuge tubes that contain membranes with 3,500 Dalton molecular weight cutoffs that will retain protein in the retentate cup but will allow buffer to flow through into the eluent cup. Use the following protocol to concentrate your samples.

1. Insert sample reservoir into filtrate cup.

2. Add 1 mL buffer to sample reservoir, being careful not to touch membrane.

3. Centrifuge for 10 minutes at 7,000 rpm and check that a significant fraction of the solution has been retained. This will establish that your micro-concentrator is free of defects.

Add your sample (3 mL limit) and centrifuge in 20 minute increments until the concentration of your protein is about 1 mg/mL or your total volume is less than 0.5 mL. For example, if your protein concentration at the start of this step is 0.2 mg/mL in 4 mL total, then you will need to reduce the volume 3-5-fold, or down to ~1 mL, in order for your protein concentration to be in the proper range. To recover your protein, place a retentate cup on the top of your sample reservoir, invert it, and centrifuge it at 3,000 rpm for 3 minutes. Your sample is now ready for dialysis.

**Polyacrylamide Gel Electrophoresis (PAGE) for Evaluating Purification**

PAGE is the single most important procedure used in the characterization of the purity of proteins. It is sensitive (μg quantities), rapid, convenient and relatively inexpensive. PAGE can serve a number of purposes. It: 1) resolves protein mixtures especially effectively when used in the two-dimensional mode, 2) permits rough quantitation, 3) provides data to calculate apparent molecular weight, and when used in conjunction with a blotting technique it 4) permits identification of protein chains by immunological and sometimes enzymological methods.

We will use PAGE as an assay system to follow the effectiveness of our purifications and to confirm the expected molecular weight of the PCP and P450 fusion constructs.

1. After removing your precast gel from its sealed plastic bag, gently pull the comb out. Don't despair if the polyacrylamide teeth left by the comb get bent -- they can be straightened up by nudging them with a hypodermic needle. Briskly snap off the bottom plastic portion. Wipe the notched side dry. Orient the sandwich so that the notched plate faces the gasket and the notches are at the top. Set the bottom of the sandwich on the bottom of the lower buffer chamber and center the plate so that the gasket seals both sides.
2. Fill the upper and lower buffer chambers with running buffer. You may need to prepare the running buffer by diluting a 10x stock appropriately using Milli-Q water.
3. Number the slots 1 through 15 on the glass plate, using a marker. You may also wish to outline the wells with the marker to easily identify them when loading your samples. Heat your samples at 100°C for 5 minutes and allow them to cool. Remember, you have 2 samples from your expression growth last week, PLUS 11 samples that you saved from your purification procedure yesterday PLUS another sample for your protein marker lane. Thus you will actually be loading a TOTAL of 14 lanes. Load your samples (no more than 20 µl total) into the wells.
4. Start the run. Attach the cover to the gel apparatus and turn on the power supply (Fig. 2). Since all the proteins are now coated with dodecyl sulfate, they have (-) charge and will move toward the (+) electrode. Make sure (+) is at the bottom! Set the power supply for "constant voltage" and set it for 90 volts and run the gel until the dye front has migrated to the bottom of the gel.

Staining and Destaining the gel

1. Once the run is complete, turn off the power, disconnect the leads, and remove the lid. Pour out the buffer prior to removing the gel sandwich. Remove the clamps and lift away the gel sandwich.
2. Snap apart the plastic plates using a spatula (or a tool that will be provided). Don't worry, it makes an awful noise if you're doing this correctly.
3. Now gently free one corner of the gel from the glass; it should slowly peel off and fall into the stain (See Fig. 3). Put the cover on the box and place on shaking table for 30 minutes. You should use a minimal amount of stain but enough to allow the gel to move freely.
4. Hold the gel in place in the box and pour the stain into a waste bottle that we've set up for you. Leave the gel soaking in about 100 mL of Safestain solution until the next day. You may wish to throw in a couple of crumpled Kim-Wipes off in a corner to more effectively destain your gels. Cover the box again.
5. Take a picture of your gel for your notebook. If you wish, you may also dry the gel on membrane using a drying frame. We can demonstrate this to anyone interested in saving their gel. Estimate the molecular weight of your protein by comparing its migration relative to your molecular weight markers. Be sure to put this information, including your assessment of the purification protocol, in your notebook.

**GEL SOLUTION RECIPES**

A. SDS gel Loading Buffer (already made up) contains per 100 ml:

30 ml 50% glycerol

3 g SDS

15 ml Stacking gel Tris buffer (above)

10 mg Bromphenol Blue tracking dye, then DH2O to 100 ml

B. Running Buffer (already made up) contains per 1 liter:

3 g Tris

14.4 g Glycine

1 g SDS

pH 8.3 (no need to adjust if measured correctly)

C. Gel Staining Solution contains per 1.1 liter:

500 ml Methanol (poison)

100 ml Glacial Acetic Acid

400 ml DH2O

Then add 2.5 g (0.25%) Coomassie Brilliant Blue R-250; mix well.

D. Gel Destaining Solution contains per 4 liters:

300 ml Glacial Acetic Acid

150 ml Methanol

DH2O (not DDH2O) to 4 liters

**Synthesis of aminoacyl-CoA derivatives.**

As with all organic chemistry reactions, this protocol should be conducted in a fume hood. In a small round-bottom flask or glass vial equipped with a rice-sized stir bar, add the Boc-protected amino acid or inhibitor starting material (1.5 equiv, 12 μmol), HBTU, and HOBt (1.4 equiv, 11.2 μmol each) to 600 μL DMF. Add DIPEA (4 equiv, 32 μmol) and stir for 5-10 minutes to allow the formation of an ester. Add Coenzyme A (1 equiv, 8 μmol) and still overnight. Deprotect by adding 1.3 mL of deprotection mixture (TFA, H_2_O/TIPS, 95/2.5/2.5 v/v/v) and stir for 2 hours. Transfer into 30 mL cold (-20 **°**C) ether and store reaction at -20C to facilitate precipitation of the product. Collect product by filtration and dissolve in 5% aqueous methanol. The product can then be purified by preparative HPLC (5-40% v/v methanol in 20 minutes, to 100% at 25 minutes) on a C18 column. Fractions containing the desired product should be collected and dried by lyopholization and dissolve in ddH_2_O to make a 10 mM working stock solution to be stored at -80 **°**C. For experimental details, see 2013 Uhlmann.

**Modification of PCP domains with Sfp.**

To load the *apo*-PCP domain with the aminoacyl group and Ppant arm, mix 100 μM of apo PCP with 150 μM aminoacyl CoA and 0.5 μM Sfp in 50 mM Tris-HCl, pH 7.4, 10 mM MgCl_2_ in a total volume of ~ 1 mL. Incubate for at least 10-30 min at 37 **°**C or at room temperature overnight. For experimental details, see 2013 Uhlmann.

**Turnover studies.**

The redox partner proteins putidaredoxin and putidaredoxin reductase are required to conduct turnover studies. These proteins can be expressed as outlined above. To the PCP modification mixture (see above), add 1 μM PdR, 5 μM PdX, 1 mM NADH, 2 μM P450_sky_, and 20 μL L-4-flurophenylalanine as an internal standard. After incubation at 30 **°**C for 30 minutes, quench the reaction by adding sodium borohydride and incubating at 37 **°**C for 15 minutes. Use centrifugation to avoid foam. Control reactions should be performed in the absence of the P450 and also in the absence of NADH. Add formic acid to a final concentration of 10%, centrifuge to remove precipitation (13000 g, 2 min) and load onto the LCMS (making sure to use the filter vials). Analyze by LCMS using a C18 column and flow rate of 0.5 mL/min with the following gradient: 5% (v/v) MeCN in H_2_O with 0.1% formic acid for 4 min, ramp to 55% MeCN over 21 min and wash with 100% MeCN for 4 minutes. For experimental details, see 2013 Uhlmann.

**One Pot Cyanylation reaction of PCP_7_**

Approximately 100 μM Holo / Apo PCP_7_ aliquots should be dialyzed overnight against 50 mM phosphate buffer, pH 7.0. Protein solutions should be treated with 8 equivalents of 5,5”-dithiobis-(2-nitrobenzoic acid) (DTNB) dissolved in 50 mM phosphate, pH 7.0 buffer for about 90 minutes to form a mixed disulfide at the terminal thiol of the 4’-Ppant arm. Addition of DTNB solution will result in the *holo* protein turning light yellow in color due to release of TNB byproduct, which absorbs at 412 nm. Apo-PCP_7_ color should not change. The DTNB-PCP_7_ Holo/Apo adduct sample should be treated with 55 molar equivalents of NaCN dissolved in 50 mM phosphate buffer, pH 7.0 and incubated on ice for about 90 minutes. Holo-PCP_7_ should turn into a more intense yellow due to further release of TNB species, while apo-PCP_7_ color should be far less intense. The cyanylated PCP_7_ can then be isolated using a Sephadex PD-10 desalting column (GE Healthcare) equilibrated in 50 mM phosphate, pH 7.0 buffer. Cyanylated PCP_7_ 4-6 fractions should then be pooled together, and the concentration should then be determined. Cyanylated PCP_7_ holo should be concentrated to 0.8-1 mM with a 3-kDa cutoff Vivaspin centrifugal concentrator (Sartorius).

**Use of UV-visible absorbance to measure concentration of proteins**

The procedure used for measuring the absorbance (optical density) of a solution is to compare the absorbance of the solution with that of a blank (consisting of your dialysis solution).

1. Remove your dialyzed sample from the dialysis cassette using your syringe and place it into an microfuge tube. Also save at least a few mLs of the dialysis buffer.

2. Take 25 μl of your dialyzed protein and add it to 1.0 mL of the dialysis buffer in a microfuge tube.

3. Fill two clean quartz micro cuvettes (1 ml capacity) with dialysis buffer ONLY. One cuvette is your reference cell and the second cuvette is your sample cell. Plastic cuvettes won't work! You will use this for “referencing” the instrument using buffer only in each cuvette.

4. You are now ready to take your spectrum using the **spectrophotometer** using the instructions provided on the next page. Replace the blank solution in the cuvette closest to you with your protein sample. The protocol for this procedure is described below in more detail.

5. After completing the protocol, compare the shape of your spectrum to the spectra in shown in the prelab lecture. Comment on the differences/similarities in your lab notebook. Does your spectrum make sense? Why or why not?

**PCR Mutagenesis and Bacterial Transformation**

Designing a mutant

Based on your hypothesis, decide on a set of mutations that you may wish to consider. The final decision for which mutation you choose to make will be determined by the outcomes of the exercise outlined below and from talking with your instructors.

The particular protocol that we will be using for creating your mutation will involve designing two overlapping 25-45 base-long oligonucleotides (or primers) that contain your mutation. Of course, you will need the DNA sequence of the gene for the fusion construct to design your primers; this is provided on the following pages. The following considerations should be made for designing mutagenic primers.

1. Both the mutagenic primers must contain the desired mutation and anneal to the same sequence on opposite strands of the plasmid.
2. Primers should be between 25 and 45 bases in length, and the melting temperature (T*_m_*) of the primers should be greater than or equal to 78°C. The following formula is commonly used for estimating the T*_m_* of primers:

T*_m_* = 81.5+0.41(%GC)-675/N-%mismatch

For calculating T*_m_*:

1. N is the primer length in bases
2. Values for %GC and % mismatch are whole numbers
3. The desired mutation should be in the middle of the primer with ~10-15 bases of correct sequence on both sides.
4. The primers optimally should have a minimum GC content of 40% and should terminate in one or more C or G bases, but this is less important.

Turn in your sequences for your two primers. Follow the formatting directions given in class carefully, so that we can order them with the minimum of delay. We will order these primers and barring unexpected delays in the synthesis of your oligonucleotide primers, you can perform the mutagenesis shortly thereafter. Stay posted, though.

**dna/PROTEIN sequences**

**P450_sky_ (http://www.ncbi.nlm.nih.gov/nuccore/327179695):**

atgggcagcagccatcatcatcatcatcacagcagcggcctggtgccgcgcggcagccat

M G S S H H H H H H S S G L V P R G S H

atgaccgcgcacaccctgcctataccggacgacatctccaccatcaatctgaccgatccc

M T A H T L P I P D D I S T I N L T D P agaacgtacgaggtgaatgacctcagcgaatactggcggcaattgcggaccacccgtccg

R T Y E V N D L S E Y W R Q L R T T R P ctgtactggcatccaccggtcggcgacgcaccgggattctgggtggtcagccggtacgcg

L Y W H P P V G D A P G F W V V S R Y A gacgtcatggcgctgtacaaggacaacaagaagctcacgtcggagaagggcaacgtgctg

D V M A L Y K D N K K L T S E K G N V L gtcacgctgctcgccgggggcgactcggcggccggcaagatgctcgccgtgaccgacggc

V T L L A G G D S A A G K M L A V T D G gccatgcaccgcggcctgcgcaacgtgctgctcaagagtttctctccgcaggcgctcaag

A M H R G L R N V L L K S F S P Q A L K ccgattgtcgaccagatccgggtcaatacgacccggctggtggtggacgccgcgcggcga

P I V D Q I R V N T T R L V V D A A R R ggtgaatgcgacttcgccgccgacgtcgccgagcagattccgctgaacaccatctcggac

G E C D F A A D V A E Q I P L N T I S D ctgctcggtgtgcccgccgcggaccgtgagttccttctcaagctcaacaagtccgcgctg

L L G V P A A D R E F L L K L N K S A L agttcggaggacgccgaccagtcggcgaccgatgcgtggctcgcccgtaacgagatcctg

S S E D A D Q S A T D A W L A R N E I L ctgtacttcagcgagctggtcgccgagcgccgcgcgaagccgaccgaggacgtcatcagc

L Y F S E L V A E R R A K P T E D V I S gttctcgccaacagcatggtggacggcaaaccgctgaccgaggaagtcatcgtcctcaat

V L A N S M V D G K P L T E E V I V L N tgctacagcctgatcctcggcggcgacgagaccagtcggctctccatgatcgactcggtg

C Y S L I L G G D E T S R L S M I D S V cagaccttcacgcagtaccccgaccagtgggagctgctgcgcgacggaaaggtgaccctg

Q T F T Q Y P D Q W E L L R D G K V T L gagtcggccaccgaggaggtgctgcgctgggcgacccccgcgatgcacttcggccgccgg

E S A T E E V L R W A T P A M H F G R R gcggtgacggacatggagctccacggccaggtgatcgccgccggtgacgtggtcacgctc

A V T D M E L H G Q V I A A G D V V T L

tggaacaactcggccaaccgggacgaggaggtcttcgcggacccgtacgccttcgacctg

W N N S A N R D E E V F A D P Y A F D L aaccggtcgcccaacaagcacatcaccttcggatacggtccgcacttctgcctcggcgcc

N R S P N K H I T F G Y G P H F C L G A tacctgggccgggccgaggtgcacgcgctgctcgacgccctgcgcacctacaccaccgga

Y L G R A E V H A L L D A L R T Y T T G ttcgagatcaccggtgagccgcagcggatccactccaacttcctcaccgggctgtcccgg

F E I T G E P Q R I H S N F L T G L S R ctgccggtgcgcatccagccgaacgaggccgcgatcgccgcctacgacagcgacaacggg

L P V R I Q P N E A A I A A Y D S D N G

gtgcggtcgtga

V R S -

**PCP_7_:**

atgggccccgacggccgcgagccgcgcaacgagaccgagtcccggctgcgccggatcttcgaggaggtgctgcacagcgaggacgtcgacgtcgaggcgaacttcttcgaactcggcgggcactcgctgcaggcgaccaagctggtcagccggatccgcagcgagttcgacgccgaactcccgctgcgcgacttcttcgagcacccgaacgtcgccggactcgccgtactgatcggcggggcc

MAPDGREPRNETESRLRRIFEEVLHSEDVDVEANFFELGGHSLQATKLVSRIRSEFDAELPLRDFFEHPNVAGLAVLIGGA

**Thioredoxin:**

atgagcgataaaattattcacctgact

M S D K I I H L T

gacagttttgacacggatgtactcaaagcggacggggcgatcctcgtcgatttctgggca

D S F D T D V L K A D G A I L V D F W A gagtggtgcggtccgtgcaaaatgatcgccccgattctggatgaaatcgctgacgaatat

E W C G P C K M I A P I L D E I A D E Y cagggcaaactgaccgttgcaaaactgaacatcgatcaaaaccctggcactgcgccgaaa

Q G K L T V A K L N I D Q N P G T A P K tatggcatccgtggtatcccgactctgctgctgttcaaaaacggtgaagtggcggcaacc

Y G I R G I P T L L L F K N G E V A A T aaagtgggtgcactgtctaaaggtcagttgaaagagttcctcgacgctaacctggccgga

K V G A L S K G Q L K E F L D A N L A G tctggcagtggttctggtcatcaccatcaccatcactccgcgggtagcgagaatctttat

S G S G S G H H H H H H S A G S E N L Y

tttcagggcgcc

F Q G A

**PCP_7_/His-Tag:**

atgggccccgacggccgcgag

M G P D G R E

ccgcgcaacgagaccgagtcccggctgcgccggatcttcgaggaggtgctgcacagcgag

P R N E T E S R L R R I F E E V L H S E gacgtcgacgtcgaggcgaacttcttcgaactcggcgggcactcgctgcaggcgaccaag

D V D V E A N F F E L G G H S L Q A T K ctggtcagccggatccgcagcgagttcgacgccgaactcccgctgcgcgacttcttcgag

L V S R I R S E F D A E L P L R D F F E cacccgaacgtcgccggactcgccgtactgatcggcggggcggccgcactcgagcaccac

H P N V A G L A V L I G G A A A L E H H

caccaccaccactga

H H H H

**PCP_10_:**

atggcgaaggacagccgc

M G K D S R

gccgcgggcaacgacaccgagcgggtcctgtgcgggctcttcgccgaggtgctcggcctg

A A G N D T E R V L C G L F A E V L G L tccgaggtcgggatcgacgacagcttcttcgacatcggcggcgacagcatcgtctccatc

S E V G I D D S F F D I G G D S I V S I aagctggtcagccgggcccgcacggcgggcatcgagttcaccgcgcgggacgtcttcgag

K L V S R A R T A G I E F T A R D V F E cacaagaccgtcgcccgtctcgcggcggccgcccgcgtcggc

H K T V A R L A A A A R V G

Mutagenesis Procedure

**Day 1 - PCR mutagenesis using Strategene QuikChange kit**

1. For each mutagenesis reaction, you will make a 25 μl reaction according to the directions below. Do not increase the amount of DNA. Using less DNA is better than using more, as too much can inhibit the reaction.

dNTP mix 1.0 μl

Pfu Turbo buffer (10X) 2.5 μl

dsDNA template 25 ng

Primer F (25 ng/μl) 5 μl

Primer R (25 ng/μl) 5 μl

ddH_2_0 to 24.5 μl

Then add 0.5 µl of *PfuTurbo* DNA polymerase (2.5 U/µl)

2. Split the sample into two 12.5 μl samples. You will put one in the PCR machine. Keep the other on ice for use as a negative control.

3. You will add your reactions to a PCR machine set up in the lab with the following cycling parameters (P450_sky_-pET28 = 6700 bases; PCPx-pET32 6200 bases):

| **Segment** | **Cycles** | **Temperature** | **Time** |
| --- | --- | --- | --- |
| 1 | 1 | 95°C | 30 seconds |
| 2 | 16 | 95°C | 30 seconds |
|  |  | 55°C | 1 minute |
|  |  | 68°C | 2 minutes/kb of plasmid length |

You may leave your samples in the PCR machine overnight as the last step of the cycling protocol will set the temperature to 4°C. Your samples may be in the lab cold room, depending on whether a TA or an instructor had the time to move your samples out of the PCR machine.

**Day 2 - *DpnI* treatment and transformation**

1. Add 0.5 µl of the *Dpn I* restriction enzyme directly to each reaction, both the sample put in the PCR machine and the negative control that you set aside.

2. Gently and thoroughly mix each reaction mixture by pipetting the solution up and down several times. Spin down the reaction mixtures in a microcentrifuge for 10-30 seconds and immediately incubate each reaction at 37°C for 1 hour to digest the parental (i.e., the non-mutated) supercoiled dsDNA.

3. Gently thaw the XL-1 Blue supercompetent cells on ice. For each strain aliquot 20 µl of the cells to transform with the control PCR reaction and 20 µl of the cells to transform with the sample reaction. Be sure to aliquot cells into a microfuge tube that has been **pre-chilled** on ice.

4. Transfer 1 µl of the *DpnI* -treated DNA from the control and 1 µl from the sample reaction to separate aliquots of the supercompetent cells. Swirl the transformation reactions gently to mix and incubate the reactions on ice for 30 minutes. Put the rest of your *Dpn I*-treated DNA in the -20°C freezer (not the refrigerator!).

5. Heat shock the transformation reactions for 45 seconds at 42°C in a water bath and then place the reactions on ice for 2 minutes.

6. Add 0.5 ml of SOC broth and incubate the transformation reactions at 37°C for 1 hour with shaking at 225-250 rpm.

7. Immediately plate the transformation reactions on LB-kan agar plates.

8. Incubate the transformation plates inverted (plate with agar on top) at 37°C overnight.

9. Seal your plates with parafilm and place them into the cold room until you are ready to start your inoculum next week. You should see lots of colonies on the plate for your sample that was put in the PCR machine. If the *DpnI* digest was complete, you should see no colonies on the control plate.

**Preparation of Mutated Plasmid DNA**

One of the common procedures used in molecular biology is “minipreps.” Minipreps are used to purify plasmid DNA from small culture volumes (1.5-5 mL). This DNA can be used for further manipulation (e.g. through restriction digestion and insertion of a new piece of DNA into the plasmid using DNA ligation), for determining the DNA sequence of a portion of the plasmid, or for introduction into a new host by DNA transformation. We will use the miniprep procedure to recover the plasmids that harbor our mutations from the XL1-blue cells so that we can introduce these plasmids into BL21 or BAP1 cells, for mutant PCP/P450 expression and purification. We cannot use the DH5α strain for expression of mutant PCP/P450 from the pET vector because the expression of the gene in this system is driven by T7 polymerase, and the gene for this polymerase is not present in the DH5α strain (as it is in the BL21 (DE3)-derived Rosetta strain and BAP1).

We will use a kit sold by Qiagen ([www.qiagen.com](http://www.qiagen.com)) to prepare the plasmid DNA. The purification involves three steps:

1. Lysis of the bacteria in an alkaline solution with SDS, a detergent. This step destroys the outer membrane of the bacterial cells and permeabilizes the cell wall. Relatively small DNA molecules are released into the solution efficiently, whereas the larger chromosomal DNA molecules remain largely associated with the cellular debris.
2. Precipitation of the cellular debris, the SDS and most of the chromosomal DNA by addition of potassium acetate.
3. Purification of the plasmid DNA from the supernatant on a small column. The plasmid binds to the column by electrostatic interactions. The column is washed to remove contaminating biomolecules, and the plasmid DNA is then eluted from the column.

Following completion of this procedure, the next step is to transform Rosetta cells with the DNA followed by plating the cells onto LB+kanamycin plates that are to be found in sleeves in the refrigerator.

# Miniprep Procedure

**Prepare two 15 mL culture tubes with 5 mL each of LB+kanamycin (0.05 mg/mL). Inoculate each with a single colony from the DH5α+ mutant protein plates. Incubate overnight with shaking at 37^o^.**

Day 1 – DNA isolation

Pellet the bacteria by centrifugation for 5 min. at 7500 rpm. Remove the supernatant and resuspend each pellet in 250 uL Buffer P1. Continue using the “QIAprep Spin Miniprep Kit”.

Day 2 –Bacterial transformation

1. Confirm the isolation of your plasmid DNA by running the DNA on a 1% agarose gel. The size standards are provided below.

2. Transform the competent cells with 2 uL of the purified plasmid DNA.

# CD instrument Procedure

**Determination of Secondary Structure by Circular Dichroism**

CD spectra of canonical alpha-helices, beta-sheets, and random coils are shown below. Note that the spectra are dramatically different from one another making it easy to distinguish amongst these classes of structures. In the absence of beta-sheet in a protein, helical content can be quantified by the absolute magnitude of the ellipticity at 222 nm in the spectrum since at this wavelength, the helical signal is the only signal that contributes to the overall CD intensity. Even with beta-sheet present in a protein, the magnitude of the spectral band at 222 nm is a good approximation of the helix content. There is not an equivalent band in beta-sheet spectra for quantifying beta-sheet content.

Circular dichroism in proteins arises fundamentally from the asymmetry of the amide bond. This asymmetry causes a differential absorption of left circularly polarized light vs right circularly polarized light (see **Chapter11** in **Protein Structure: A Practical Approach**). Absorption of light causes an excitation of the electrons of the amide bond and the energy of absorption and the number of excited electrons depend on the type of secondary structure in which the amide bond is found. Electrons in the amide bond typically absorb light at 215 nm, so most CD spectral bands appear in this range as evidenced by the spectra shown in the prelab lecture.

**Part A - Turning the Instrument On**

1. Open valve on liquid nitrogen tank attached to the regulator and nitrogen line (6). Adjust the small black knob if necessary so the pressure float is just above the red arrow. The instrument **MUST be purged for one hour before striking the lamp**.

2. Turn on left circuit breaker (1). When lamp ready light is lit (4), strike the lamp by pushing red button. The lamp indicator should be red. You may need to repeat this several times. Allow the lamp to warm up for at least 30 minutes. Turn on the water bath, press the on button in front panel (5).

3. Turn on right circuit breaker (2). This supplies electricity to the computer and electronics. Turn on the computer and monitor.

4. You will be saving your data on the hard drive and then moving it to your personal space on the College storage server or onto the Biochemistry_Lab course space on the storage server. There is an icon on the desktop for accessing the server.

5. PLEASE SIGN IN AND LOG YOUR HOURS IN THE LOG BOOK!

**Part B - Acquiring a Spectrum**

1. Take your sample spectrum first. Place your sample into your 1 mm cuvette then place the cuvette into the 1 mm adaptor. Place the cuvette plus adaptor into the sample compartment (5). To do this, lift the lid off the sample compartment, remove the second small, black lid on the cuvette thermostatted holder, and place the cuvette into the holder with the narrow side facing you. Replace the lids.

2. First, you will set all the parameters for your experiment. You will do this by selecting “Read Configuration File” from the file menu. Find the Biochemistry folder and load the “NRPS1spec.cfg” file. If you want to view and/or change these parameters, choose the “Configure experiment” menu. Make the appropriate changes in this window and the window that opens when you select “Wavelength Configuration” from the “Experiment Configuration” menu. Be sure to Exit/Save and then save the configuration file to an appropriate name with the .cfg extension. The parameters are designed to optimize the signal to noise of your spectrum:

a) Bandwidth = 1.5 nm. This increases the amount of light entering the sample by allowing a wider span of wavelengths. It also has the effect of reducing the resolution of your observed spectral bands.

b) The Wavelength Start and End values are set to 198 nm and 260 nm, respectively, for this experiment.

c) Step size (listed as “Sample Every”) = 0.5 nm . This collects data every 0.5 nm.

d) Averaging Time = 3 sec . Increases signal to noise by greater time averaging.

4. Hit the “RUN EXPERIMENT” box. Data collection will take about 5-10 minutes. You may change your axis limits during data collection by either building a box around the accumulating data or by right-clicking in the graph. Adjust your scale accordingly.

5. After your spectrum has been collected you need to give your spectrum a name and save it to disk. Choose “Save experiment in data browser and hard drive”.

6. To acquire a blank, or reference, spectrum, replace the sample in your cuvette with buffer. Then repeat steps 4and 5.

**Part C - Correcting the Spectrum**

You will now need to subtract your blank spectrum from your sample spectrum. It is common for spectra to be offset from true zero millidegrees due to lamp and cuvette artifacts. So the first step is to reset the offset to zero. This needs to be done for both your blank and sample spectra. Do the following steps:

1. You will need to review your sample and blank spectra to figure out how far from zero millidegrees your spectra have been offset. Do this by simply zooming in on the region around 250 nm and taking your best guess as to the average offset from zero. You only need to be accurate to the tenths decimal place. To view your data, select “Data Review” from the “Displays” menu and choose “Wavelength”. Then choose “Left Multi-Data Set” from the “Axis Definitions” menu and select all of the CD signals that you wish to view.

2. Having established an offset value, you are ready to modify your spectra accordingly (both sample and reference). Under “Math Operations”, select “Wavelength Experiments”. Start by choosing “Select Data Set A”. A new Data Browser frame will open. Select the appropriate Experiment and CD signal from the “Wavelength Experiments” folder. With the appropriate folder opened for your CD signal, hit the “Select Data Set” button off to the left of your browser window. Your experiment name and data set name should now be indicated to the left. Choose “Add Constant” from the “Operation or Constant” menu bar and then type the offset value into the “Constant” window, using a value of the opposite sign of course. Give your newly modified spectrum a new filename and keep it associated with your experiment name that you loaded for data set A. Select “Calculate” , then select “Return”. Repeat this procedure for your blank spectrum.

3. To subtract your blank spectrum from your sample spectrum, select your new files that represent your offset spectra as data sets A and B and choose “Subtract Sata Sets” from the Operation menu bar. Be sure to enter a new filename for your difference spectrum. YOU MUST PERFORM THE OPERATIONS IN THE ORDER THAT THEY APPEAR IN THIS MENU!

4. The units for your spectrum are in mdegs. You will need to report your final spectrum in terms of mean residue ellipticity (MRE) using units of deg cm^2^ dmol^-1^. To do the conversion, use the following relationship:

MRE = (100 * mdeg) / (pathlength * [protein] * #aa's)

The pathlength is 0.1 cm and the protein concentration should be in units of mM.

5. To change your units from mdeg to MRE, be sure that you have this information from step 4 in hand and modify your modify your difference spectrum (from step 3 above) using this factor by entering your filename representing your difference spectrum as data set A and choosing “Convert to Molar Ellipticity” from the Operation menu bar. Be sure to give this file a new name. Save your files and move your files to your storage server location. To save them to the hard drive, select “Load Data Set” from the File menu and select your experiments within the “Wavelength Experiments” folder in the data browser window. Then select the “Save Data Set -> Disk” button.

**Part D - Turning off the Instrument**

1. Make sure all your data that you want to keep are saved on the hard drive and have been copied over to the storage server.

2. Quit from the software and Choose Shutdown from the Start menu to shut down the computer. Turn off the monitor. **LOG** in the lamp hours prior step 3 in the log book plus any remarks.

3. Turn off both circuit breakers (1) and (2). Turn off nitrogen valve (6) and water bath (5).

There is a paper copy of this document in the folder CD instructions next to the CD instrument.

**Determination of Protein-Protein Interactions, Size, and Molecular Weight by Analytical Ultracentrifugation**

The analytical ultracentrifuge is the most versatile, rigorous, and accurate means for determining the molecular weight and the hydrodynamic and thermodynamic properties of a protein. No other technique is capable of providing the same range of information with a comparable level of precision and accuracy. The reason for this is that the method of sedimentation analysis is firmly based in thermodynamics. All terms in the equations describing sedimentation behavior are experimentally determinable.

The analytical ultracentrifuge is unsurpassed for the direct measurement of molecular weights of proteins in the native state and as they exist in solution, without having to rely on calibration and without having to make assumptions concerning shape. Two basic analytical experiments are used: sedimentation velocity, and sedimentation equilibrium.

When a protein is suspended in a solvent and subjected to a centrifugal field, three forces act on the particle. First, there is a sedimenting, or centrifugal force, *F_s_*, proportional to the mass of the particle and the acceleration. In a spinning rotor, the acceleration is determined by the distance of the protein from the axis of rotation, *r*, and the square of the angular velocity, ω (in radians per second):

*F_s_* = *m*ω^2^*r*

where *m* is the mass in grams of a single protein. Second, there is a buoyant force, *F*_b_, that is equal to the weight of fluid displaced:

*F*_b_ = *mv*_bar_ρ

Where ρ is the density of the solvent and *v*_bar_ is the volume in mL that each gram of the solute occupies in solution. Since proteins are more dense than the typical solution conditions, the particle will begin to sediment.

In a velocity sedimentation experiment, the goal is to observe the protein concentration boundary move from the meniscus to the “bottom” of the cell, representing sedimentation behavior of the individual particles, so a rotor speed is chosen to optimize movement of the concentration boundary within a reasonable time frame (3-9 hours).

See: https://www.embl.de/pepcore/pepcore_services/protocols/biophysical_characterisation/au/

The goal is to collect a reasonable number of traces of the boundary profiles as a function of time, so that sufficient data are collected to provide with robust data analysis. A representative set of boundary profiles are shown here below. Analysis of the collective dataset allows one to identify the number of boundaries that are present, and after appropriate data manipulation, can be represented as a distribution of species as a function of size, as shown in the second graph:

See: *http://www.ap-lab.com/sedimentation_velocity.htm*

In an equilibrium sedimentation experiment (we will probably not do this type of experiment), as the concentration of protein at the bottom of the cell begins to increase, a third force, that of diffusion, opposes that of sedimentation. After an appropriate period of time, the forces of diffusion and buoyancy and the opposing centrifugal force allow the protein to reach equilibrium in all parts of the solution column and, for a single, ideal solute component, the concentration of the solute increases exponentially towards the cell bottom. At sedimentation equilibrium, the processes of sedimentation and diffusion are balanced; the concentration distribution from the top of the cell to the bottom no longer changes with time, and is a function of molecular weight. Ideally, equilibrium sedimentation experiments are run at lower speeds than velocity sedimentation experiments, since we don’t want to pellet the protein at the “bottom” of the cell.

Technically, equilibrium sedimentation is measured in real time by a monochromater mounted inside the ultracentrifuge. The monochromater shines light through cells loaded into the rotor and the protein absorbance is monitored by a photomultiplier in the base of the chamber. The monochromater scans across the cells, which hold the sample between two quartz windows. The measured signal shows an increased absorbance as the monochromater moves away from the center of axis of rotation, as protein piles up in the bottom of the cell. The absorbance can be plotted as a function of radial distance and takes on the form of an exponential. These data can be analyzed using an expression based on the equations given above:

This equation states that the protein concentration at any position, r, can be predicted from a reference protein concentration position, C_0_, through the exponential function shown. This exponential function contains the parameters described above, including a molecular weight term, M. The data can be analyzed by the use of a fitting function containing this expression; thus the molecular weight of a protein can be extracted from such data.
