# Supplementary material for: Uncovering protein–protein interactions through a team-based undergraduate biochemistry course
Source: PLoS Biol. 2017 Nov 1;15(11):e2003145. doi: 10.1371/journal.pbio.2003145 (PMC5683658; doi:10.1371/journal.pbio.2003145)

**Supplementary Information for**

**Uncovering Protein-Protein Interactions through a Team-based Undergraduate Biochemistry Course**

David L. Cookmeyer^1‡^, Emily S. Winesett^1‡^, Bashkim Kokona^2^, Adam R. Huff^1^, Sabina Aliev^1‡^, Noah B. Bloch^2‡^, Joshua A. Bulos^1‡^, Irene L. Evans^1‡^, Christian R. Fagre^2‡^, Kerilyn N. Godbe^1‡^, Maryna Khromava^1‡^, Daniel M. Konstantinovsky^1‡^, Alexander E. Lafrance^2‡^, Alexandra J. Lamacki^1‡^, Robert C. Parry^1‡^, Jeanne M. Quinn^2‡^, Alana M. Thurston^1‡^, Kathleen J. S. Tsai^1‡^, Aurelio Mollo^1‡^, Max J. Cryle^3,4^, Robert Fairman^2*^, Louise K. Charkoudian^1*^

^1^Department of Chemistry, Haverford College, Haverford PA 19041, USA

^2^Department of Biology, Haverford College, Haverford PA 19041, USA

^3^The Monash Biomedical Discovery Institute, EMBL Australia, Monash University, Clayton, Victoria 3800, Australia

^4^The Department of Biochemistry and Molecular Biology and ARC Centre of Excellence in Advanced Molecular Imaging, Monash University, Clayton, Victoria 3800, Australia

^5^Undergraduate student enrolled in 2015 Biochemistry 390 (“Biochemistry Superlab”)

**Assessment**

Haverford College requires faculty to assess a subset of their courses every year, and in the sciences, we typically aim to evaluate, both quantitatively and qualitatively, the student perception of their experience. We often aim to get student feedback that focuses largely on improving the course in future iterations, rather than on an absolute measure of faculty competence. The first section below provides the questions that were asked of the students, along with representative quotes that highlight successes as well as suggestions for improvement. A brief summary of the overarching conclusions is provided at the end of this section.

The second section provides a glimpse at how our students fair against a larger cohort of students at other institutions in similar types of interdisciplinary (integrated) teaching laboratory settings. This is a long-standing project funded for many years by a generous grant from the Howard Hughes Medical Institute (HHMI) provided to researchers at Grinnell College. We have participated sporadically in this survey, and plan to participate more regularly as we move towards a more systematic institutional commitment to measuring student learning outcomes.

**Course Evaluation select quotes (3 from each question)**

1. **What were your expectations for this course? Were they fulfilled? If yes, how? In what ways were your expectations not fulfilled?**

- I was expecting to have a rigorous lab experience where we tackled a somewhat independent project in biochemistry. These were completely fulfilled, and we actually had more independence than I expected, which was not at all a bad thing. I was expecting to learn new and relevant lab techniques, which we did do.
- I expected to learn biochemical techniques and to carry out an independent research project. My expectations were fulfilled in that I learned many biochemical techniques. I did not expect to make such little progress on the independent project, though.
- I expected that this course would give me an understanding of techniques that are used in the biochemistry field along with a better understanding of what biochemistry encompasses. I feel like most of my Haverford career has been spent either thinking I was going to be a biology major or a chemistry major, and before this semester I wasn't exactly sure the extent of the overlap between the two subjects. I think that both of my expectations were fulfilled. My project allowed me to focus on both synthesis of compounds and binding studies and so I felt like I was able to learn and become familiar with many new techniques. I also thought that the amount of background we gained in the biochemistry was really important for me because it allowed me to bridge concepts together.

1. **What were the most accessible/manageable aspects of Superlab for you? Why?**

- The most accessible aspects were learning about and executing new techniques and protocols in lab. I really enjoy being able to work with new methods, and found we had ample preparation to execute new methods we learned on our own.
- It became manageable at the end. After we had repeated things, it became less stressful and time consuming to start an experiment. We were able to come in when we wanted, but we didn't have a lot of direction or guidance after the start up time. It was hard to know if we were expected to keep going with something that wasn't working or when to try something else.
- I really enjoyed the journal clubs. For the first time, I feel like I can pick up a scientific paper about anything in biochemistry and feel confident that I will really understand what's going on without spending a day looking up terms. I am really grateful for this. I also really enjoyed the class discussions of papers, because for the first time, my classmates and I seemed to feel that we can discuss papers on a high level.

1. **What were the most difficult aspects of Superlab for you? Why? How did you meet those challenges?**

- The time was often a lot, and then in weeks where we had less to do I was concerned that we were underperforming. I think talking to the professors and determining expectations was key here. As for the material itself, being in the cold room for four hours purifying protein was taxing, but kind of fun.
- Most difficult aspects was the large amounts of additional lab time. I didn't realize how much time experiments would take, and it was hard to plan accordingly to try and stay on schedule. The professors informed us we overloaded work and could relax. So I guess these challenges were met with realizing that schedules in lab don't always work out the way you plan and learning how to improvise around that.
- I would say the most challenging thing was desigining and planning out our own research project. Additionally, many of the lab techniques we used were relatively new to me, so I had to go through some troubleshooting as we were doing things. I found that this challenge was much easier to meet with a partner, especially with respect to planning out the research project.

1. **What were the most engaging aspects of Superlab for you? Why?**

- Wet lab work in general. I love getting my hands dirty in lab and putting new experimental techniques I learned to use. And troubleshooting is so gratifying when you figure out the problem.
- I highly enjoyed the troubleshooting process, in an odd way. The professors were very open to discussion of problems we ran into in our projects, and the process of creative problem solving and succesfully surpassing experimental obstacles was fulfilling.
- I loved the independent nature of the projects. I liked that everybody was doing something different, but that we were all working with the same system so we could collaborate. I also liked how the guest lecturers complemented our studies. It was really neat to be able to read an article and then get to speak to the authors themselves just a few days later!

1. **How did the instructors help you achieve your goals in this course? Comment on approachability, enthusiasm, availability, willingness to help, etc.**

- They were great helping to plan both short term (what are we doing today) and long term (where do we want this experiment to be three weeks from now). They were also great with trouble-shooting and willing to help us work around our otherwise busy schedules, which was great.

1. **How useful were the journal articles and journal clubs for helping you understand the goals of the Superlab project and experimental protocols?**

- It was an interesting loop. The articles were really hard to understand before we had some experience with the system and experimental techniques, but they also provided a framework to structure what we learned about.
- I thought that the initial journal clubs in which we read were really important because it allowed us to gain a thorough understanding of the Skyllamycin system and NRPSs. I didn't think that the subsequent articles necessarily helped with my understanding of the superlab project, though. They were interesting and I thought that they did a good job of showing different techniques or applications of biochemistry, but I didn't get as much out of them because they seemed a little tangential.
- One of my personal goals for junior year was to become better at navigating and understanding the primary literature (since you don't get that much practice with that during freshmen and sophomore years). I really feel like I succeeded in meeting that goal, largely thanks to superlab (especially since so many of the journal articles that we read were accompanied by a guest lecture by the authors themselves!). I also like journal club discussions as a method of learning how to understand primary literature (I've always been a bigger fan of discussion based classes).

1. **What other advice would you offer to instructors about how to teach this course in the future?**

- Be more open about the time commitment - frontloading the coursework paid off in the end. Had I known ahead of time how much work this class would be, I'd have adjusted my schedule accordingly and probably adjusted my extracurriculars too to accommodate this class, because it was well worth the effort.
- Make sure students know that they most likely won't get as far as they think -- and that a majority of what you learn is problem solving for when things go wrong!
- I wish I had a more concrete idea of what the other teams were up to throughout the second quarter. I feel like I had a good idea of what the other lab teams were up to when we were brainstorming ideas for our hypotheses presentations, but once we started our independent projects I feel like I only knew what me and my lab partner were doing and I didn't know what the other groups were up to. It would have been helpful to have a better idea of where the other groups were in their projects in order to foster greater collaboration between groups.

It seems that expectations were largely met, bridging both chemistry and biology effectively. Some were a bit disappointed that more time was spent on trouble-shooting than on learning techniques. There may have been a split amongst the students in wanting the experience to mirror a research approach versus a broader exposure to various biochemical techniques.

**RISC analysis (18 students)**

Description of the survey is taken from the Spring 2015 report:

The RISC survey (Research on the Integrated Science Curriculum carried out by David Lopatto and Leslie Jaworski from Grinnel College, funded by HHMI) offers a comparison of learning benefits between interdisciplinary course experiences and other experiences, particularly in science education. The pre-course survey collects student data based upon demographic questions, reasons for taking the course, level of experience on various course elements, science attitudes, and learning style. The post-course survey parallels the pre-course survey and includes additional questions that focus on student estimates of learning gains in specified course elements, estimates of learning benefits that parallel questions in other surveys, such as CURE, overall evaluation of the experience, and science attitudes. More information can be found at:

<https://www.grinnell.edu/academics/areas/psychology/assessments/risc-survey>

The RISC survey used for this study may be found at:

<http://ww3.haverford.edu/chemistry/Charkoudian/in_the_classroom/>

**Summary**

1. Number of students participating: 18 students filled out the pre-course survey and 16 students filled out the post-course survey. Typically, students fill out the post-course survey immediately after completing a course. We were interested in a longitudinal review, asking how students felt about their learning gains 1.5 years after graduation, in conjunction with preparation of this work.
2. Demographic information
   1. 7 male, 11 female
   2. 7 have graduate school plans, 11 have medical school plans
3. Highlights of survey information comparing Haverford College student assessment against that for the entire cohort of surveyed students
   1. Course element gains
      1. Our students reported gains above that reported for all students surveyed in areas tied to independent research experiences, such as:
         1. Problems where no one knows the outcomes;
         2. A problem where students have input into process or topic;
         3. Reading of scientific literature from multiple fields;
         4. Presentation of intellectual work;
         5. Taking responsibility for part of a project.
      2. Our students reported gains below that reported for all students surveyed for:
         1. Use of textbooks;
         2. Integration of science and non-science disciplines;
         3. Using personal values to motivate the study of problems.
   2. Learning gains (reported in August 2017)
      1. Our students reported learning gains above that reported for all students surveyed for a large number of areas tied to independent research (although the differences here are largely within one standard deviation). Some notable areas include:
         1. Tolerance for obstacles faced in the research process
         2. Readiness for more demanding research
         3. Learning laboratory techniques
         4. Ability to read and understand primary literature
         5. Understanding of how scientists think
   3. Learning style (restructure vs. no structure) – our students are “convergers”, indicating that they particularly value active experimentation with abstract conceptualization (these students fell well outside the norm of the larger cohort) – we postulate that this is a reflection of our emphasis on molecular and chemical biology

**Graphical representation of course and learning gains, comparing pre-course and post-course surveys, and against the larger cohort of participating institutions**

1. Course element gains
   1. Elements surveyed

- Scripted lab or problem where students know outcome
- Lab or problem where only instructor knows outcome
- Problems where no one knows the outcome
- A least one problem assigned and structured by instructor
- A problem where students have input into process or topic
- A project or problem entirely of student design
- Work individually
- Connect personal experience to the course problem or problems
- Work in small groups or teams
- Disciplinary knowledge needs to be accurate and fair
- Read primary scientific literature within one field or discipline
- Coursework from multiple disciplines or areas of study
- Collect data
- Analyze data
- Approach problems in different and conflicting ways
- Present intellectual work in written papers or reports
- Present intellectual work in posters
- Use instruments/materials from other field of study
- Critique work of other students
- Listen to lectures
- Work with peers from other disciplines or field of study
- Find similarities and differences between disciplines
- Work on problem sets
- Take tests in class
- Define a problem and refine definition while solving problem
- Engage in class discussion
- Maintain lab notebook
- Integrate ideas from two or more sciences in problem solving
- Study an interdisciplinary problem
- Spend entire course on one or a few problems
- Read a textbook
- Integrate ideas from both science and non-science disciplines
- Attempt complete understanding of a complex problem
- Ask questions that implicate more than one discipline in answer
- Talk with faculty members from other disciplines
- Read primary literature from multiple fields of study
- Present intellectual work orally
- Become responsible for a part of a project
- Translate specialized language of a discipline into the language of other disciplines
- Write a research proposal
- New insights emerge from considering multiple disciplines
- Work as a whole class
- Judge relative contribution of disciplines to problem solution
- Create new metaphors, analogies or models to understand
- Computer modeling of complex systems
- Study problems with simultaneous, interactive multiple causes
- Engage in experiential learning
- Use personal values to motivate the study of a problem


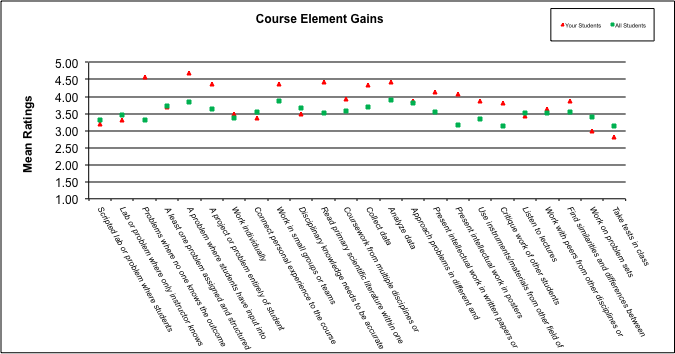


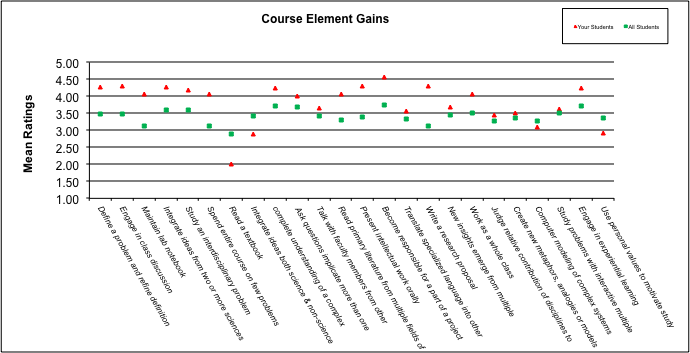


1. Learning gains (post-course survey only)
   1. Learning surveyed

- Clarification of a career path
- Skill in the interpretation of results
- Tolerance for obstacles faced in the research process
- Readiness for more demanding research
- Understanding how knowledge is constructed
- Understanding the research process in your field
- Ability to integrate theory and practice
- Understanding of how scientists work on real problems
- Understanding that scientific assertions require supporting evidence
- Ability to analyze data and other information
- Understanding science
- Learning ethical conduct in your field
- Learning laboratory techniques
- Ability to read and understand primary literature
- Skill in how to give an effective oral presentation
- Skill in science writing
- Self-confidence
- Understanding of how scientists think
- Learning to work independently
- Becoming part of a learning community
- Confidence in my potential as a teacher of science
  1. Learning gains results


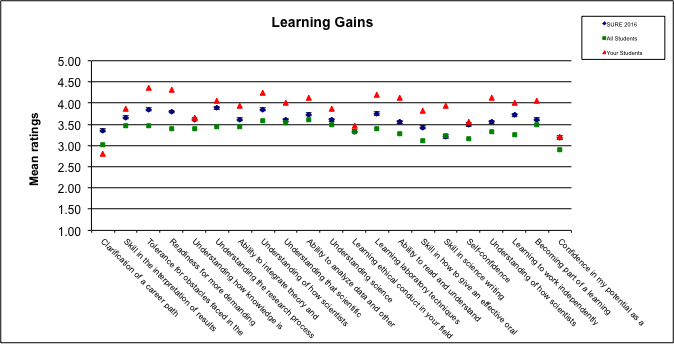

Supplement: S1 Assessment — RISC, Research on the Integrated Science Curriculum. (DOCX) [file pbio.2003145.s015.docx]
